# Rapid Eye Movement Sleep, Sleep Continuity and Slow Wave Sleep as Predictors of Cognition, Mood, and Subjective Sleep Quality in Healthy Men and Women, Aged 20–84 Years

**DOI:** 10.3389/fpsyt.2018.00255

**Published:** 2018-06-22

**Authors:** Ciro della Monica, Sigurd Johnsen, Giuseppe Atzori, John A. Groeger, Derk-Jan Dijk

**Affiliations:** ^1^Surrey Clinical Research Centre, Faculty of Health and Medical Sciences, University of Surrey, Guildford, United Kingdom; ^2^Division of Psychology, Nottingham Trent University, Nottingham, United Kingdom; ^3^Surrey Sleep Research Centre, Faculty of Health and Medical Sciences, University of Surrey, Guildford, United Kingdom

**Keywords:** sleep, cognition, aging, executive function, neurodegeneration, slow waves, REM sleep, sex differences

## Abstract

Sleep and its sub-states are assumed to be important for brain function across the lifespan but which aspects of sleep associate with various aspects of cognition, mood and self-reported sleep quality has not yet been established in detail. Sleep was quantified by polysomnography, quantitative Electroencephalogram (EEG) analysis and self-report in 206 healthy men and women, aged 20–84 years, without sleep complaints. Waking brain function was quantified by five assessments scheduled across the day covering objectively assessed performance across cognitive domains including sustained attention and arousal, decision and response time, motor and sequence control, working memory, and executive function as well as self-reports of alertness, mood and affect. Controlled for age and sex, self-reported sleep quality was negatively associated with number of awakenings and positively associated with the duration of Rapid Eye Movement (REM) sleep, but no significant associations with Slow Wave Sleep (SWS) measures were observed. Controlling only for age showed that associations between objective and subjective sleep quality were much stronger in women than in men. Analysis of 51 performance measures demonstrated that, after controlling for age and sex, fewer awakenings and more REM sleep were associated significantly with better performance on the Goal Neglect task, which is a test of executive function. Factor analysis of the individual performance measures identified four latent variables labeled Mood/Arousal, Response Time, Accuracy, and Visual Perceptual Sensitivity. Whereas Mood/Arousal improved with age, Response Times became slower, while Accuracy and Visual perceptual sensitivity showed little change with age. After controlling for sex and age, nominally significant association between sleep and factor scores were observed such that Response Times were faster with more SWS, and Accuracy was reduced where individuals woke more often or had less REM sleep. These data identify a positive contribution of SWS to processing speed and in particular highlight the importance of sleep continuity and REM sleep for subjective sleep quality and performance accuracy across the adult lifespan. These findings warrant further investigation of the contribution of sleep continuity and REM sleep to brain function.

## Introduction

Sleep of good quality and sufficient duration is assumed to benefit health and brain function (1). Epidemiological studies indicate that both extreme short and extreme long self-reported sleep durations are negatively associated with cognitive function and mood (2–5). In interventional studies, acute and chronic sleep deprivation (6, 7), and disruption of both Slow Wave Sleep (SWS) and sleep continuity (8, 9) all lead to an increase in daytime sleepiness, a reduction in subjective sleep quality as well as a reduction in sustained attention and processing speed. Few recent interventional studies on the effects of Rapid Eye Movement (REM) sleep deprivation on next day functioning are available. The effects of sleep on brain function may be mediated by total sleep time (TST), sleep continuity or specific aspects of sleep structure such as slow waves, sleep spindles or REM sleep (10). Hypotheses about the mechanisms by which sleep contributes to waking function have emphasized the role of sleep in plasticity and synaptic homeostasis (11, 12), brain system reorganization including the possible transfer of recently acquired memories from hippocampus to neocortex (13), biochemical-physiological hypotheses such as sleep mediated clearance of metabolites from the brain (14) or reversal of inflammation (15, 16). Although experiments demonstrating an impact of insufficient sleep on brain function have been primarily conducted in healthy young participants, studies of sleep in older people indicate that sleep also contributes to brain functioning in the later stages of the life span (10, 17). Some studies have reported correlations between sleep and cognition in particular age groups [e.g., (18)] and patient groups such as those with mild cognitive impairment (19). In these studies age has rarely been treated as a continuous variable and in very few studies has sleep been assessed by polysomnography (PSG) (20, 21). In fact, whether inter-individual variation in aspects of EEG assessed sleep may explain inter-individual variation in subjective sleep quality and cognition across the healthy life span, beyond the explanatory power of age itself, has not been firmly established (10). Addressing this question is of relevance because changes in sleep are prominent in mental disorders across the life span (22) and are also prominent in neurodegenerative conditions such as Parkinson's disease, Alzheimer's disease and other forms of dementia (23, 24), and may be predictive of cognitive decline and neurodegeneration (25).

Here we document the co-variation in cognition and sleep in a cross-sectional study of carefully screened healthy men and women aged 20–84 years and investigate whether sex^1^ and age modulate some of the associations between sleep and waking performance. Sleep was quantified using standardized polysomnographical methods, including quantitative analysis of slow wave and sleep spindle activity, and self-report measures. Cognition was quantified using a test battery which was applied five times across the day following the nocturnal sleep recordings. The test battery assessed various aspects of waking performance including self-report of alertness, mood, and affect and objective measures of sustained attention & arousal, decision & response time, motor & sequence control, working memory, and executive function. This battery has previously been shown to be sensitive to SWS disruption, partial sleep deprivation, and age-related cognitive decline (6, 9).

## Materials and methods

### Ethics, conduct, and data sources

The protocols were approved by external independent ethics committees (Quorn Research Review Committee and Ravenscourt Ethics Committee) and all participants provided written informed consent before any study specific procedures were initiated. Analyses presented in this report are based on the baseline data collected in two clinical trials conducted in accordance with the principles of Good Clinical Practice and the Declaration of Helsinki. The experiments were designed to determine the functional significance of SWS disruption and pharmacological enhancement of SWS. Whereas the effects of SWS disruption and enhancement have been in part reported elsewhere (9, 26, 27) the combined baseline data and the association analyses are presented here for the first time.

### Participants, inclusion, exclusion criteria

In both trials care was taken to only include physically, neurologically and mentally healthy participants, with a BMI between 19 and 33 kg/m^2^, and without clinically significant sleep disorders or cognitive deficits. Inclusion/exclusion criteria have been described in detail elsewhere (9, 26, 27). In brief, physical health was ascertained through a general health questionnaire, medical history, physical exam, blood biochemistry, hematology, etc. Potential participants who were 65 and older completed the Mini Mental State Examination and those with a score less than or equal 25 were excluded. All participants completed a psychiatric assessment (the Mini International Neuropsychiatric Interview MINI) (28) and the National Adult Reading Test (NART) (29) as an assessment of verbal IQ. Participants completed the Pittsburgh Sleep Quality Index (PSQI) (30) and were excluded if their score was greater than five. Chronic sleep complaints according to DSM-IV, were exclusionary and a self-reported typical sleep duration between 6.5 and 8.5 h with a typical bedtime between 22:00 and 00:00 h were required. Habitual sleep duration and timing was verified by 7 days of actigraphy prior to a full clinical polysomnographic sleep screen. Participants with an apnoea-hypopnoea index > 15/h, or periodic leg movements (arousal index > 15/h) and those with a history of other sleep disorders such as narcolepsy, circadian rhythm sleep disorder, parasomnias, or current shift work were excluded. Smoking more than 5 cigarettes per day, drinking more than 3 alcohol containing beverages per day, taking hypnotics, central nervous system (CNS) depressants, stimulants, diet pills, antihistamines, herbal preparations, systemic glucocorticoids were all exclusionary. Participants were instructed to maintain a regular sleep schedule (with bedtimes at 23:00 and wake times at 07:00) prior to the admission to the Clinical Research Centre (CRC).

### Sleep assessments: polysomnographic (PSG) recordings and analyses

Sleep recordings were made in sound attenuated, temperature controlled, windowless individual bedrooms with lights-off at 23:00 and lights-on at 07:00 h, using a Compumedics Siesta digital EEG machine. Signals were digitized at 256 (EEG, EMG) or 128 Hz (EOG/ECG) and stored at 128 Hz for the EEG and EOG/EMG. The low and high frequency filters were set at 0.3 and 30 or 70 Hz for the EEG and EOG, and at 10 and 70 or 100 Hz for the EMG. PSG recordings were scored to the criteria of (31) by Registered Polysomnographic Technologists. For the present analyses the following PSG variables were considered: Latency to Persistent Sleep (LPS), Total Sleep Time (TST), sleep efficiency (SE, i.e., Total sleep time/Time in bed), Stage 1, Stage 2, Stage 4 sleep, Slow Wave Sleep (SWS, i.e., Stage 3 + Stage 4), REM sleep, and Number of Awakenings (NAW). In addition, spectral analysis was conducted on a Central EEG derivation, after exclusion of visually identified segments that could lead to spurious power spectra (e.g., fast- or slow-frequency artifacts such as that which occurs with body movement or excessive sweating). Power spectra were computed for 4 s epochs by applying a Fast Fourier Transform routine implemented in VitaScore (Temec, The Netherlands). Sleep stage specific power spectra with a resolution of 1 Hz were computed by combining four 0.25 Hz power density values and averaging of the 4-s power spectra per 30 s for a specific sleep stage. For the current analyses we considered power in the Slow Wave Activity band (SWA; 0.75–4.5 Hz) and sigma band 12.25–15.0 Hz (which correlates well with sleep spindle activity and will be referred to a Spindle Frequency Activity (SFA) (32) during NREM sleep. SWA and SFA are expressed either in absolute values or as percentages of total power (0.25–32 Hz) in NREM sleep (SWA%, SFA%). The latter measure assesses the contribution of a specific band after controlling for individual differences in total power.

### Sleep assessments: subjective sleep quality

Upon awakening participants reported on both their Quality of Sleep Last Night (sQoS) and How Refreshed they felt upon Awakening (sRuA), using a Visual Analogue Scale (VAS). They also reported Sleep Latency (sSleep-Lat), Number of Awakenings (sNAW) and Total Duration of Night Awakenings. The latter question was apparently misinterpreted by several participants and these data were excluded from the data presented here. This instrument which is aimed at measuring perceived sleep quality, has previously been shown to be sensitive to changes in sleep quality induced by SWS disruption (26) and Traffic Noise (27).

### Assessment of daytime functioning/ cognition

In both protocols, daytime functioning was assessed five times per day with the start of computer controlled tests at 8:00, 10:00, 12:00, 14:00, and 16:00 h. Except where bespoke equipment was required (e.g., Critical Flicker Fusion), tests ran on identical computers with screen refresh rates of 60 Hz, running Active X, C#, and Exactics code to control stimulus presentation, response detection, and related timing. The computerized tests assessed performance across a variety of cognitive domains: Mood & Affect, Sustained Attention & Arousal, Decision & Response Time, Motor & Sequence Control, Working Memory, and Executive Function [see (9) for details]. Here we only report those measures which were obtained in both protocols.

### Statistical analyses and data presentation

Our general approach was to identify age-related changes in sleep and waking function, their association and the extent to which variation in sleep parameters may explain variation in waking function, beyond the extent to which age or sex predicts waking function.

We used ANOVA (PROC MIXED) and non-parametric (Kendall-tau) correlation (PROC CORR) implemented in SAS (version 9.2 or later). We selected Kendall correlation/mediation analyses because it does not make assumptions regarding the distribution of variables or the dependency of these variables on age. We opted for a bivariate correlation, rather than a multiple regression, approach because our main objective was to identify the relative strength of PSG predictors, where the PSG predictors are standard sleep measures. Due to the interdependence of many sleep variables a multivariate approach may not have allowed the identification of the “predictive” value of individual sleep variables.

For those waking function measures for which assessments were scheduled for five times per day, a simple average value was computed when 3 or more observations were available. Otherwise the data were considered missing. Among the performance measures used in these analyses there were 252 missing values out of 9,430 values (i.e., 3%). Among the sleep variables, for the subjective sleep variables there were no missing observations from the dataset of 824 compared to the objective sleep variables where there were 162 missing values out of a total dataset of 2,516 observations (i.e., 6%). Range of number of observations contributing to the various analyses have been indicated in the tables. We conducted analyses on single performance measures as well as on factor scores derived from factor analyses (PROC FACTOR) of the performance data. This factor analysis identified four latent “performance” variables (negMood/Arousal, Response Time, Accuracy, and Visual Perceptual Sensitivity); these are inferred variables, from the directly observed performance variables, which represent fundamental aspects of task performance. Psychometric variables were factor analyzed with retention of four factors and Varimax rotation as indicated by the “broken stick” approach. Derived variables (e.g., difference between N-back difficulty levels) were not included in the factor analyses, but were included in other analyses. Overall Goal Neglect and overall Simple Reaction Time were also excluded in favor of their component measures. The total number of variables used in the factor analysis was 41. For the factor analyses missing data in the performance variables were mean imputed.

Although in most Kendall correlation analyses we conserved all data as one sample, some analyses were conducted separately for men and women or separately for the three age groups (20–30, 31–64, 65–84 years labeled as young, middle-aged, and older, see Supplemental Table 1 for summary demographics) or for each of six age groups (defined below). Correlation coefficients were compared between groups by transforming Kendall's tau to a standard normal according to Siegel (33).

For ANOVA the cohort was subdivided in six sets defined as young-1 (20–29 years; *n* = 59, 24F); young-2 (30–39 years; *n* = 20; 9F), middle-aged-1(40–49 years; *n* = 31; 17F), middle-aged-2 (50–59 years; *n* = 25; 17F); older-1 (60–69 years; *n* = 44; 30F), and older-2 (70–84 years *n* = 27; 17F).

In several analyses, age and sex were entered as co-variates. In light of the large number of comparisons and correlations we present both nominal significances as well as multiplicity adjusted significance. Multiplicity correction was based on the False-Discovery Rate procedure (*p* < 0.05, in most cases but *p* < 0.1 are reported in some cases) as proposed by Benjamini and Hochberg (34) and Benjamini and Yekutieli (35).

Effect sizes were reported as small, medium and large with associated Cohen's *d*-values of 0.2, 0.5, and 0.8. Kendall's τ values were converted into Cohen's *d* according to Gilpin (36).

Participants only contributed one “observation;” some participants took part in both clinical trials from which this data is derived and so for these individuals only data from the first trial were included in the analysis.

## Results

### Polysomnographically assessed sleep structure: effects of age and sex

**Figure 1:**
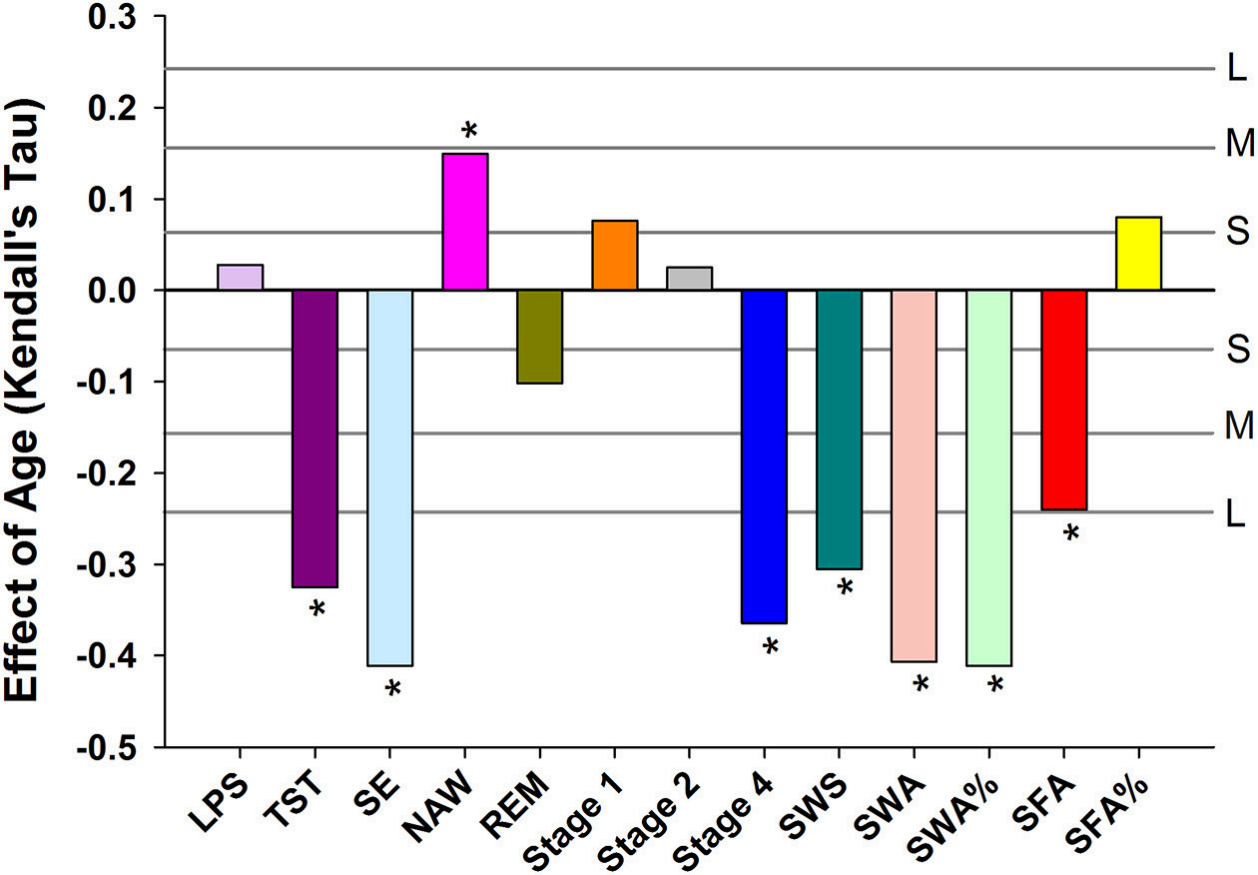
Age related changes in sleep EEG parameters when controlling for sex. For reference, the horizontal lines indicate the corresponding Cohen's *d* effect size: S, small, *d* = 0.2; M, medium, *d* = 0.5; H, high, *d* = 0.8. ^*^ indicate significant effects following FDR (False-Discovery Rate) correction (*p* < 0.05). LPS, latency to persistent sleep (min); TST, total sleep time (min); SE, sleep efficiency (%); NAW, number of awakenings; REM, rapid eye movement; Stage 1, duration of stage 1 sleep (min); Stage 2, duration of stage 2 sleep (min); Stage 4, duration of stage 4 sleep (min); SWS, slow wave sleep; SWA, slow wave activity (μV^2^); SWA%, slow wave activity in percentage of total power; SFA, sigma activity (μV^2^); SFA%, sigma activity in percentage of total power.

Objective sleep measures are summarized separately for three age categories and men and women in Supplemental Table 2. Analyzed per six age groups, ANOVA identified significant age-related reductions in Total Sleep Time (TST), Stage 4, SWS, Slow Wave Activity (SWA), SWA%, Spindle Frequency Activity (SFA), REM duration and Sleep Efficiency (SE) and an increase in Number of Awakenings (NAW). Significant effects of sex were observed such that women had more TST, Stage 4, SWS, SWA, SWA%, SFA, and SE but reduced SFA% and Stage 1. Age affected women and men differently, i.e., the interaction between age and sex was significant for TST, Stage 1, Stage 4, SWS, SWA, and SE such that sleep was better preserved with aging in women (Supplemental Table 3). The effect of age on the objective sleep variables as quantified by Kendall's tau, controlled for effects of sex, were significant for SE, TST, Stage 4, SWS, SFA, and NAW. Largest effects were observed for SE and Slow Wave Sleep related measures (Figure 1).

### Subjective sleep measures: effects of age and sex

Subjective Sleep Latency, Quality of Sleep, Number of Night Awakenings and Refreshed upon awakening were not significantly affected by either age or sex (Supplemental Table 4).

### Association between EEG sleep and subjective sleep measures controlled for age and sex

**Figure 2:**
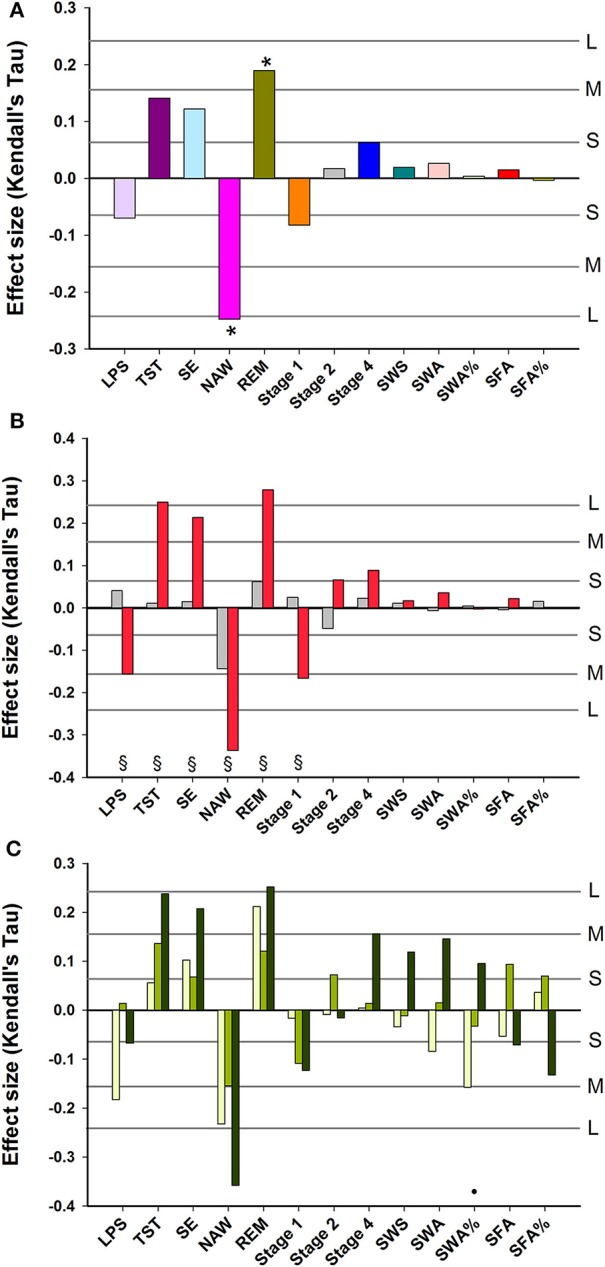
Association between EEG sleep parameters and subjective quality of sleep: **(A)** data controlled for sex and age; **(B)** data controlled for age and shown separately for men (gray bars) and women (red bars); **(C)** data controlled for sex and age and shown separately for three age groups: Young, 20–30 years (yellow bars); Middle-aged, 31–64 years (light green bars); and Older, 65–84 years (dark green bars). For reference, the horizontal lines indicate the corresponding Cohen's *d* effect size: S, small, *d* = 0.2; M, medium, *d* = 0.5; H, high, *d* = 0.8. ^*^ indicate significant effects following FDR (False-Discovery Rate) correction (*p* < 0.05). § indicates a significant difference (*p* < 0.05) between men and women Kendall's Tau-values. • indicates a significant difference (*p* < 0.05) between younger and older Kendall's Tau-values. LPS, latency to persistent sleep (min); TST, total sleep time (min); SE, sleep efficiency (%); NAW, number of awakenings; REM, rapid eye movement; Stage 1, duration of stage 1 sleep (min); Stage 2, duration of stage 2 sleep (min); Stage 4, duration of stage 4 sleep (min); SWS, slow wave sleep; SWA, slow wave activity (μV^2^); SWA%, slow wave activity in percentage of total power; SFA, sigma activity (μV^2^); SFA%, sigma activity in percentage of total power.

After controlling for age and sex and multiplicity, Subjective Sleep Quality was negatively and significantly associated with NAW, and positively with REM sleep. No significant associations between Subjective Sleep Quality and Slow Wave Sleep related measures were observed (Figure 2A). Associations between objective sleep parameters (LPS, TST, SE, NAW, Stage 1) and Subjective Sleep Quality were significantly stronger in women than men (Figure 2B). Associations between objective sleep parameters and Subjective Sleep Quality were similar in young, middle-aged and older participants, although SWA% contributed negatively to sleep quality in the young and positively in older participants and this difference in the association was significant (Figure 2C).

Associations between objective sleep parameters and self-reported feelings of being “Refreshed upon Awakening” and their modulation by sex and age (see Supplemental Figure 1) were similar to those for Subjective Sleep Quality (associations between objective sleep parameters and all subjective sleep parameters after controlling for age and sex are summarized in Supplemental Table 5). Of note are the significant associations between subjective and objective NAW and subjective and objective sleep latency.

### Waking performance: effects of age and sex

**Figure 3:**
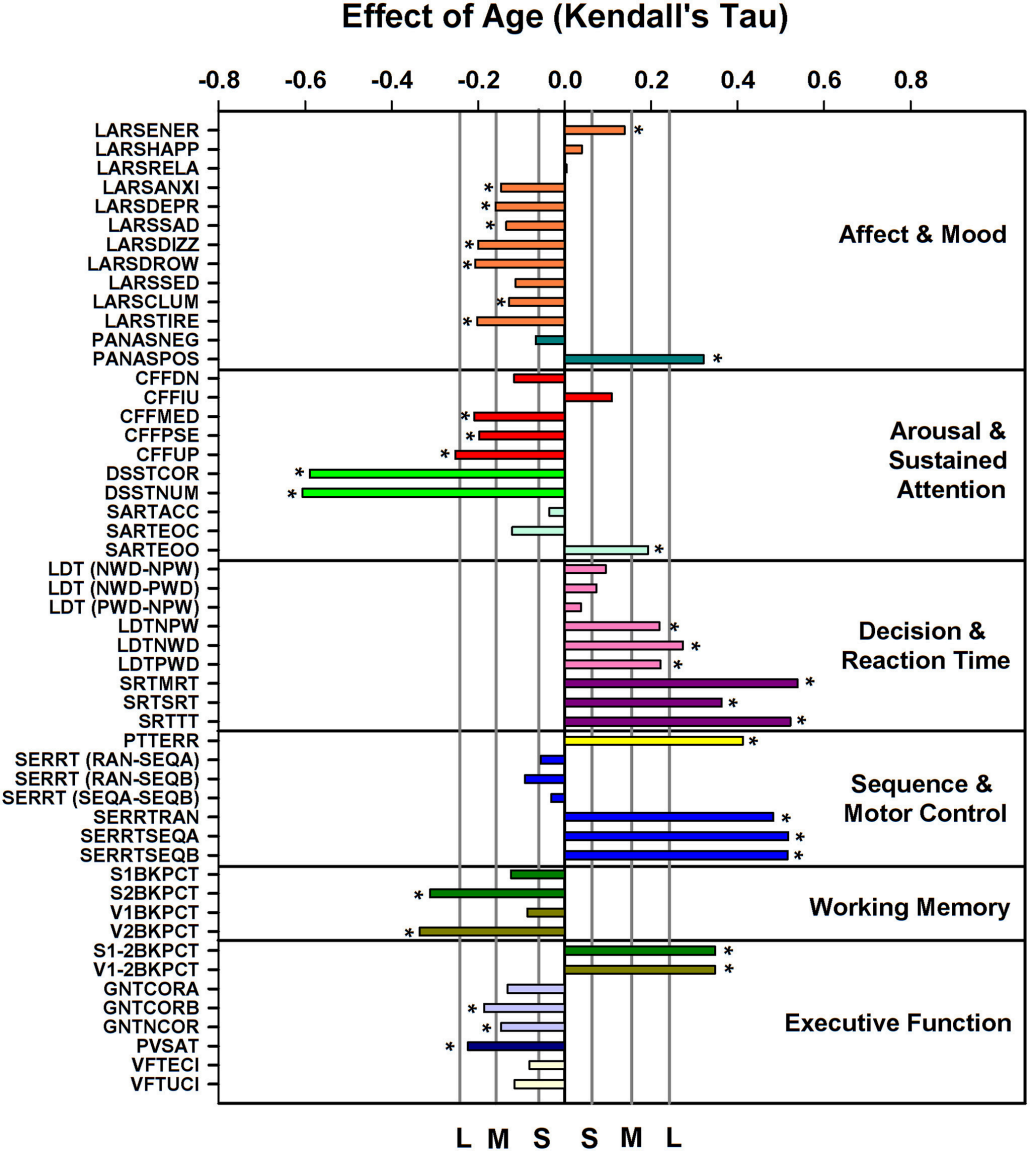
Effect of age on cognition/performance variables, plotted according to cognitive domain, when controlling for sex. For reference, vertical lines indicate the corresponding Cohen's *d* effect size: S, small, *d* = 0.2; M, medium, *d* = 0.5; H, high, *d* = 0.8. ^*^ indicate significant effects following FDR (False-Discovery Rate) correction (*p* < 0.05). Performance variables are described in Supplemental Table 6.

ANOVA applied to the data subdivided in six age groups and men and women indicated that 34 out of 51 performance measures (see Supplemental Table 6 for description of variables) were affected by age (see Supplemental Table 7); sex explained a significant proportion of the variance for 10 measures. Men performed better on the Critical Flicker Fusion Test, The Simple Reaction Time Task, The Serial Reaction Time Task, The Pursuit Tracking Task, and the Goal Neglect Task. A significant interaction between sex and age was observed for five performance measures derived from the Serial Reaction Time Task (including both the reaction time and “switching” components of the task), and the Paced Visual Serial Addition Task such that performance was better preserved with aging in men than in women. After partialling out sex the nominal *p*-value of Kendall's tau for the association between age and performance measures was <0.05 for 40 out of 51 measures. After correcting for multiplicity these associations remained significant for 32 measures (Figure 3). Largest associations were observed for throughput, speed and error measures of the Digit-Symbol Substitution, the Pursuit Tracking task, and the Serial Reaction time task. Deterioration with age was also observed for measures of working memory (Verbal and Spatial N-back performance), decision and reaction time measures of the Lexical Decision and Simple Reaction Time task and executive function measures [e.g., difference between N2-N1 back performance (with an increase in the difference indication a deterioration of executive function), performance on the Goal Neglect Task and on the Paced Visual Serial Addition Task]. Linear Analogue Rating Scales of affect and mood improved with age such that older people wore more energetic, less anxious, etc. and felt more positive according to the Positive and Negative Affect Scale (PANAS).

### Association between EEG sleep and waking performance

**Figure 4:**
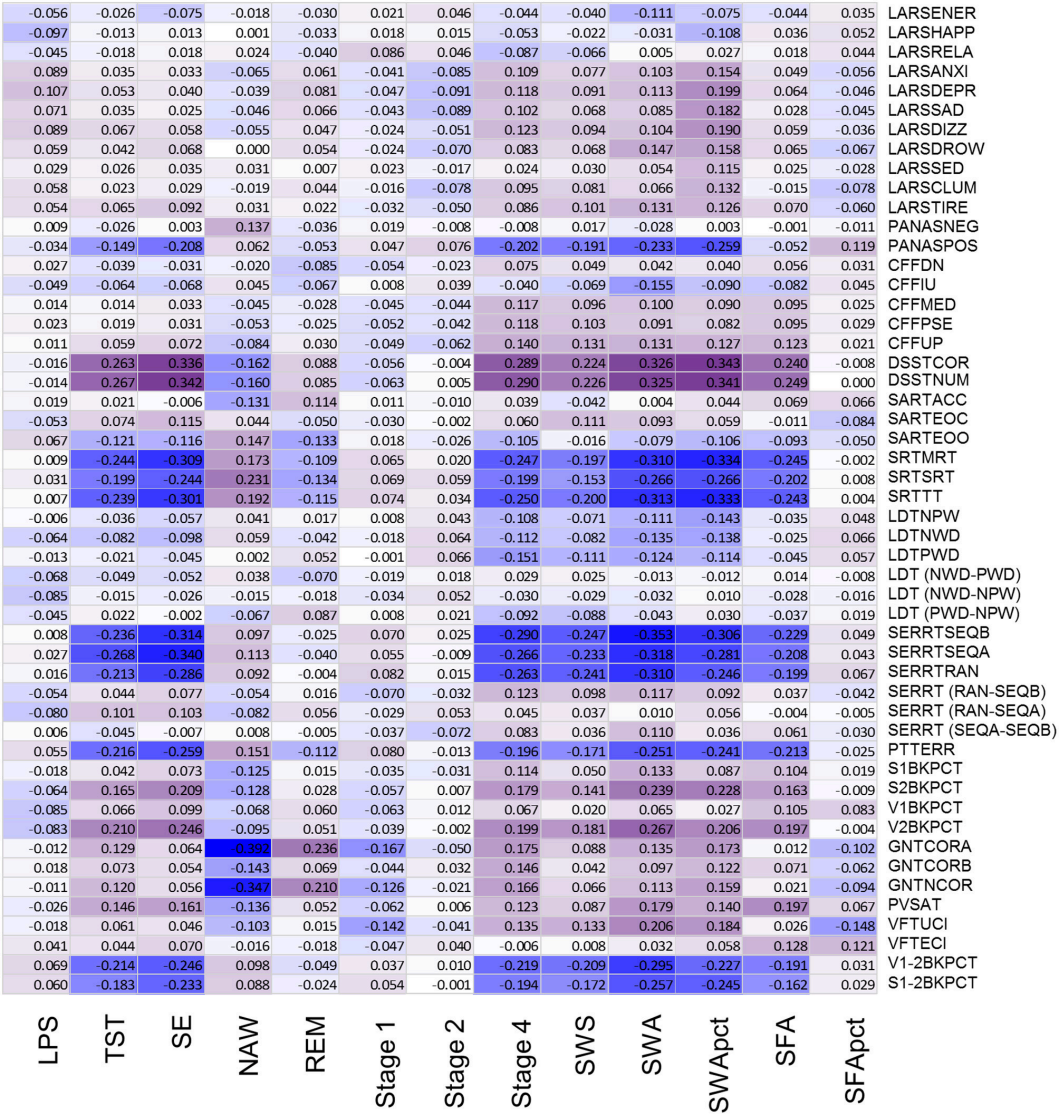
Association between objective sleep parameters and performance variables, controlled for sex, through color coded Kendall's tau values. LPS, latency to persistent sleep (min); TST, total sleep time (min); SE, sleep efficiency (%); NAW, number of awakenings; REM, rapid eye movement; Stage 1, duration of stage 1 sleep (min); Stage 2, duration of stage 2 sleep (min); Stage 4, duration of stage 4 sleep (min); SWS, slow wave sleep; SWA, slow wave activity (μV^2^); SWApct, slow wave activity in percentage of total power; SFA, sigma activity (μV^2^); SFApct, sigma activity in percentage of total power. Performance variables are described in Supplemental Table 6.

**Figure 5:**
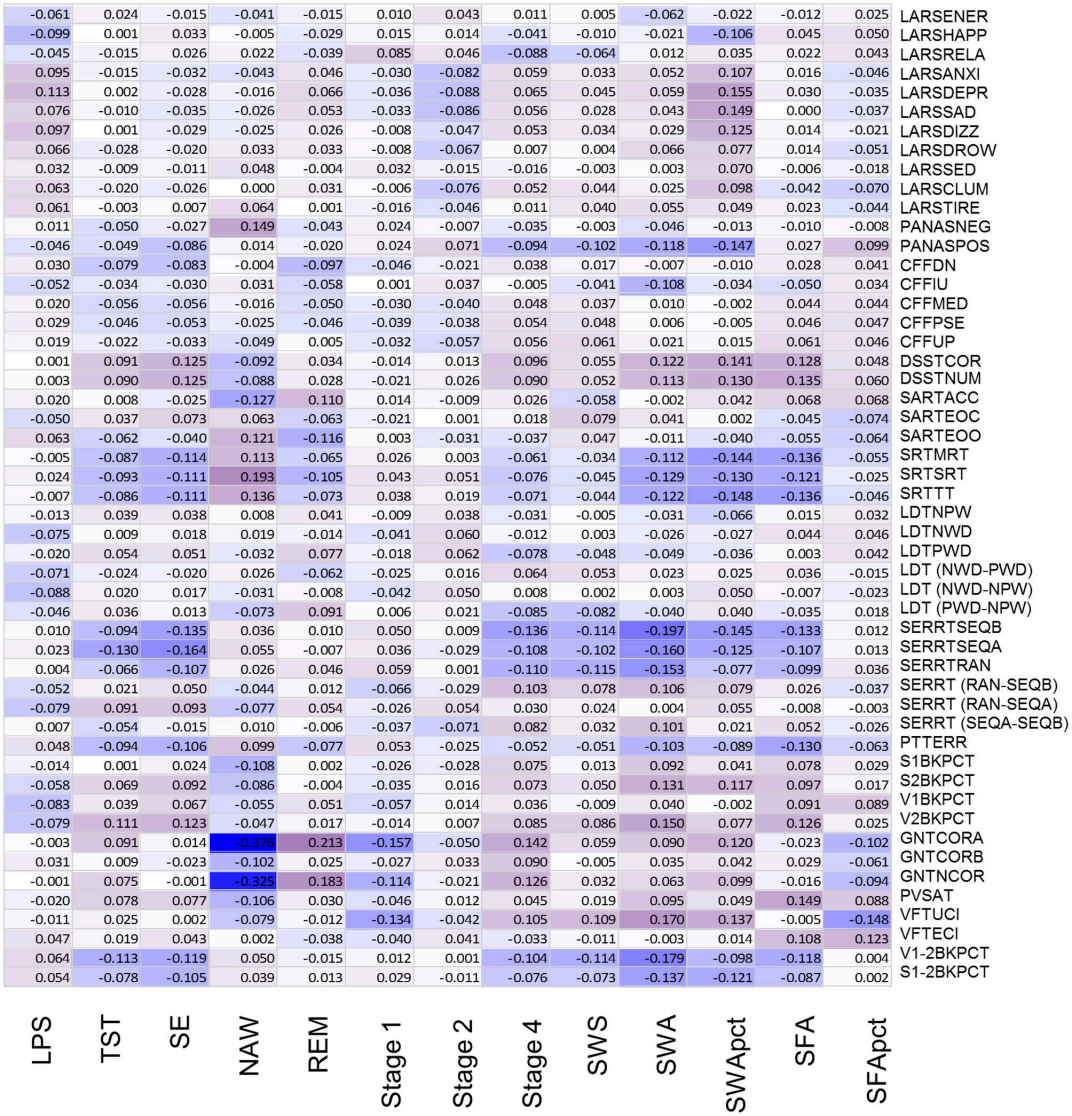
Association between objective sleep parameters and performance variables, controlled for sex and age, through color coded Kendall's tau values. LPS, latency to persistent sleep (min); TST, total sleep time (min); SE, sleep efficiency (%); NAW, number of awakenings; REM, rapid eye movement; Stage 1, duration of stage 1 sleep (min); Stage 2, duration of stage 2 sleep (min); Stage 4, duration of stage 4 sleep (min); SWS, slow wave sleep; SWA, slow wave activity (μV^2^); SWApct, slow wave activity in percentage of total power; SFA, sigma activity (μV^2^); SFApct, sigma activity in percentage of total power. Performance variables are described in Supplemental Table 6.

After controlling for sex and multiplicity, 111 out of 663 correlations between PSG sleep parameters and individual performance measures were significant. Strongest associations were observed for the EEG sleep measures, NAW and SE and performance on the Goal Neglect Task after switching of the instruction (GNTCORA) (Figure 4, Supplemental Table 8) and throughput measures of the DSST. Several Slow Wave Sleep related measures also correlated strongly with reaction time measures and throughput measures. REM sleep correlated positively with GNTCORA. After controlling for both sex and age as well as multiplicity the magnitude of the associations between EEG sleep and performance as well as the number of significant associations was reduced (Figure 5, Supplemental Table 9). Correlations between NAW and pre- and post-switch measures of the Goal Neglect Task survived a FDR of 0.05 (nominal *p*-value 1.1^*^10^−12^ and 7.7^*^10^−10^). NAW also correlated positively with Total Response Time on a reaction time task (SRTSRT; nominal *p*-value 5.8^*^10^−5^, FDR *p* < 0.1). Duration of REM sleep correlated positively with post-switch Goal Neglect (GNTCORA, nominal *p*-value 5.62^*^10^−5^, FDR *p* < 0.1), indicating better switching performance with more REM sleep. For Slow Wave Sleep measures, smallest nominal *p*-values were observed for SWS and stable serial reaction performance (SERRTSEQB, *p* < 0.00021) and SWA and the difference in performance between the Verbal 1-back and Verbal 2-back test (*p* < 0.000366), with more SWA being associated with better performance on these tasks. However, none of the associations between Slow Wave Sleep related measures and performance measures were significant at the FDR *p* < 0.05 or *p* < 0.1 level (Figure 5).

### Waking performance measures: data reduction by factor analysis and association with age

**Figure 6:**
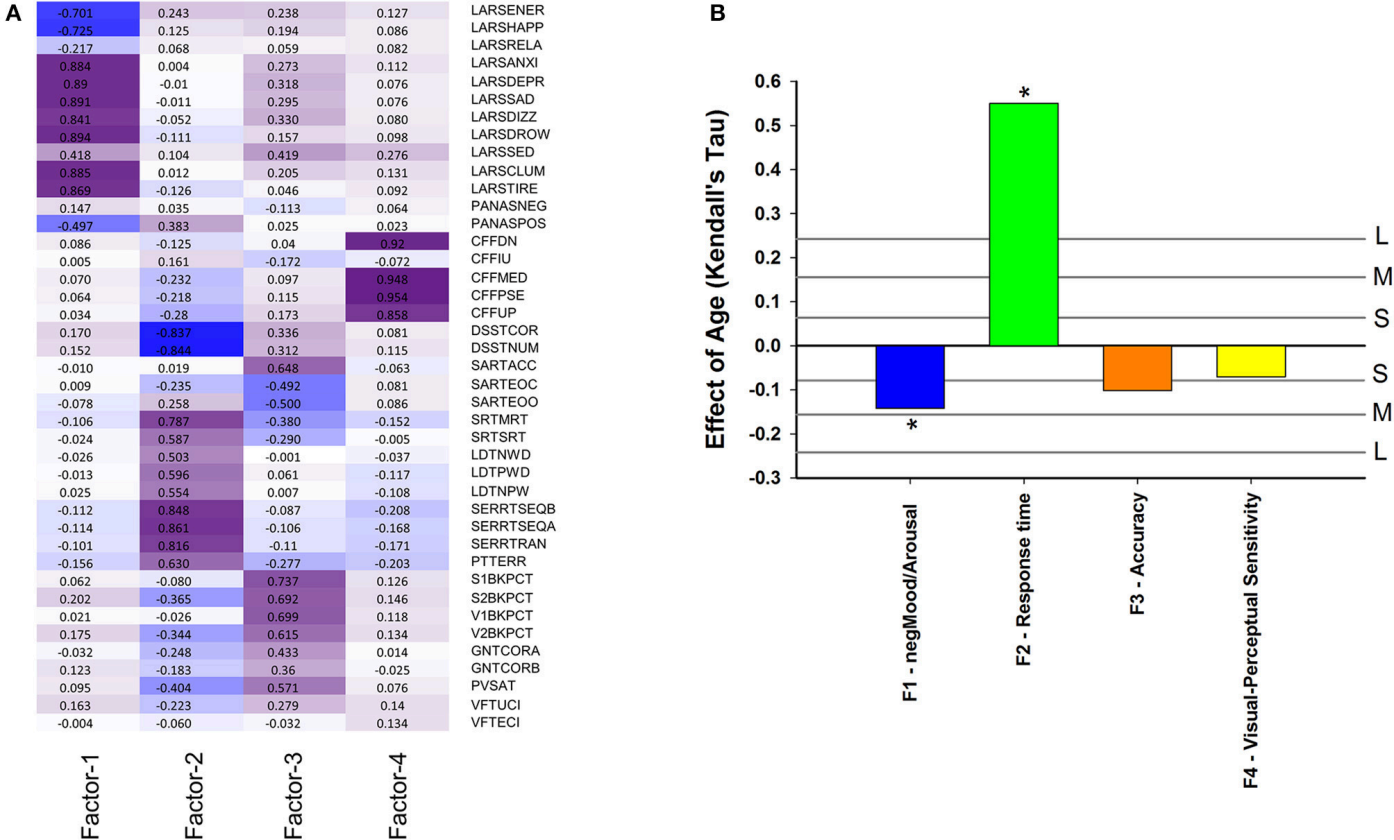
**(A)** Factor loading after Varimax rotation: contribution of performance variables to four factors based on these performance variables: Factor 1 (negMood/Arousal), Factor 2 (Response time), Factor 3 (Accuracy), Factor 4 (Visual-Perceptual Sensitivity). Performance variables are described in Supplemental Table 6; **(B)** Effect of age on cognition/performance factors when controlling for sex. For reference, vertical lines indicate the corresponding Cohen's *d* effect size: S, small, *d* = 0.2; M, medium, *d* = 0.5; H, high, *d* = 0.8. ^*^ indicate significant effects following FDR (False-Discovery Rate) correction (*p* < 0.05).

Performance variables, excluding derived measures, were subjected to factor analysis to identify latent variables and investigate how they change with age and associate with EEG sleep. The eigenvalues of four latent variables (factors) exceeded estimated broken stick eigenvalues. The observed eigenvalues were 11.34, 5.57, 3.11, and 2.97: while the corresponding broken stick eigenvalues computed as 1/*k* + 1/(*k*+1) + - - - + 1/41 produced 4.30 (*k* = 1), 3.30 (*k* = 2), 2.80 (*k* = 3), and 2.47 (*k* = 4). After Varimax rotation these four factors explained 7.2, 7.0, 4.9, and 3.9% of the variance. Factor 1 was labeled negMood/Arousal because it has most negative loadings on ratings on linear analogue scales for alertness, happiness and energy and most positive weightings on drowsiness, tiredness, clumsiness, sadness (Figure 6A). Thus an increase in this factor score indicates lower alertness, happiness and energy, and more drowsiness, tiredness, clumsiness, and sadness. This factor score decreased with age, i.e., older participants were more alert and happy, and less tired and sad (Figure 6B) (*p*_nom_ = 0.0025, *p*_FDR_ < 0.05). Factor 2 was labeled “Response Time” because it had the largest negative weighting on Digit Symbol Substitution Test measures and largest positive weightings on reaction time in the Serial Reaction Time Task. It should be noted that because derived measured were excluded, this is purely a response speed effect. Response Time was positively associated with age (Figure 6B), i.e., older participants were slower (*p*_nom_ < 10^−16^). Factor 3 was labeled “Accuracy” because it had largest negative weightings on errors of omissions and commissions on the sustained attention to response time and largest positive weightings on accuracy measures of the verbal and spatial n-backs and negatively on error measures from Sustained Attention to Response Task. This factor decreased with age (nominal *p*_nom_ = 0.030, *p*_FDR_ < 0.1). Factor 4 was labeled Visual-Perceptual Sensitivity because it had largest weighting on measures of the Critical Flicker Fusion test. This factor did not change significantly with age.

### EEG sleep as predictor of mood, response time, accuracy, and visual-perceptual sensitivity

**Figure 7:**
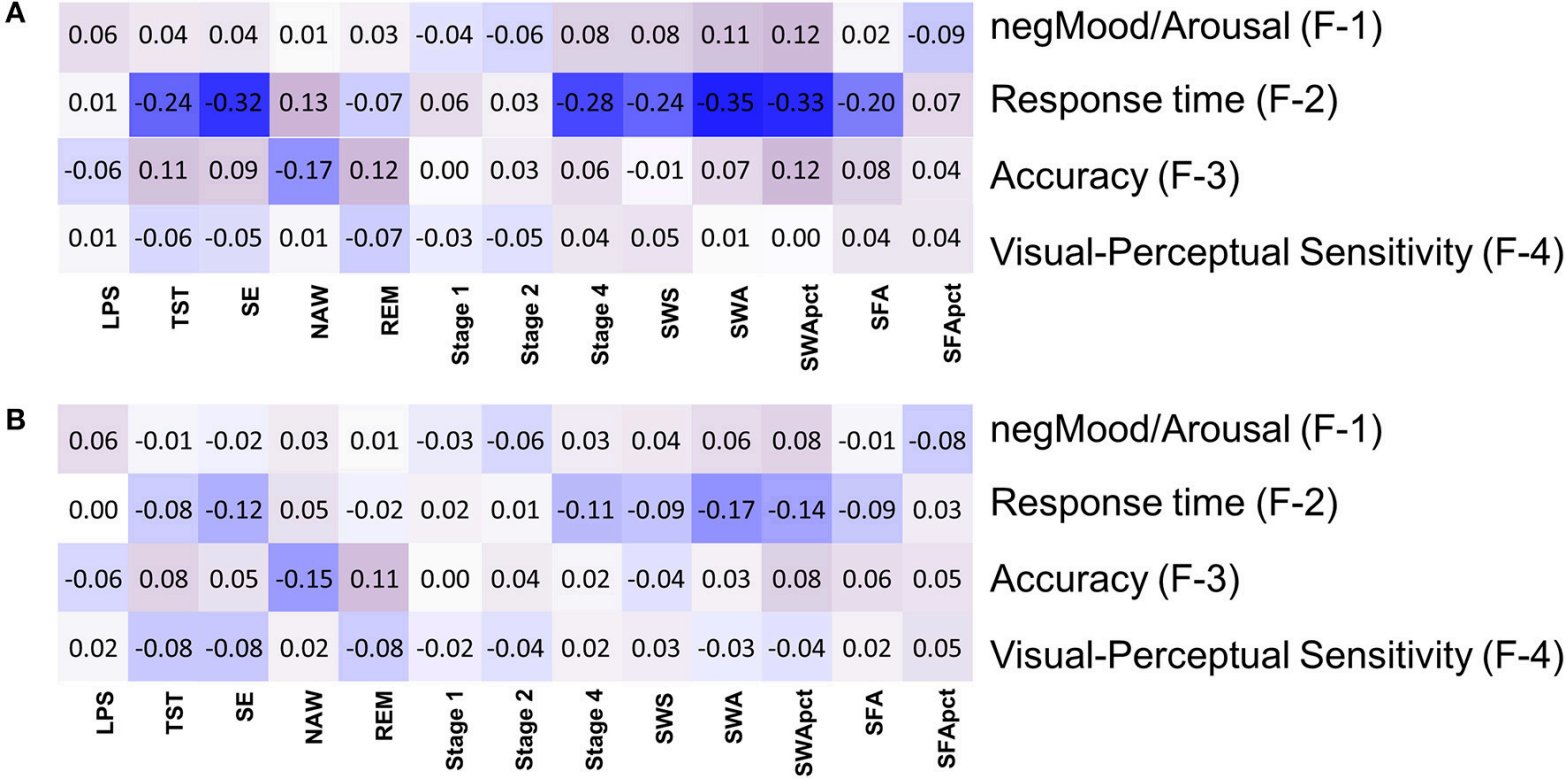
Association between objective sleep parameters and four factors based on performance variables: **(A)** controlled for sex, **(B)** controlled for sex and age. LPS, latency to persistent sleep (min); TST, total sleep time (min); SE, sleep efficiency (%); NAW, number of awakenings; REM, rapid eye movement; Stage 1, duration of stage 1 sleep (min); Stage 2, duration of stage 2 sleep (min); Stage 4, duration of stage 4 sleep (min); SWS, slow wave sleep; SWA, slow wave activity (μV^2^); SWApct, slow wave activity in percentage of total power; SFA, sigma activity (μV^2^); SFApct, sigma activity in percentage of total power.

After controlling for sex, but not for age, 14 out of 52 correlations between EEG sleep parameters and Factor scores had a nominal *p* < 0.05. Of these, 8 remained significant after multiplicity correction (*p*_FDR_ < 0.05) (Figure 7A, Supplemental Table 10). Seven of these correlations were with Response Time, such that SWA (tau = −0.35; *p*_nom_ ≤ 4.4^*^10^−12^); SE (tau = −0.32; *p*_nom_ ≤ 2.2^*^10^−11^); SWA% (tau = −0.33; *p*_nom_ ≤ 4.2^*^10^−11^); Stage 4 (tau = −0.28; *p*_nom_ ≤ 2.7^*^10^−9^); SWS (tau = −0.24; *p*_nom_ ≤ 5.9^*^10^−7^); SFA (tau = −0.20; *p*_nom_ ≤ 7.1^*^10^−5^), and TST (tau = −0.24; *p*_nom_ ≤ 3.9^*^10^−7^) all correlated negatively with Response Time i.e., positively with speed. With respect to Accuracy, the only other association significant after correction for multiplicity (*p*_FDR_ < 0.05) was with NAW (tau = −0.17; *p*_nom_ ≤ 0.0005), i.e., more awakenings were associated with lower Accuracy. REM sleep duration was correlated positively with Accuracy (tau = 0.123; *p*_nom_ ≤ 0.01) but this was not significant at the FDR *p* = 0.05 level.

After controlling for sex and age, six out of 52 correlations between EEG sleep parameters and factors scores had significant nominal *p*-values but none survived multiplicity adjustment at the p < 0.1 level. For Self-reported Mood and Arousal no nominally significant associations with EEG sleep parameters were observed. For Response Time, SWA (tau = −0.17; *p*_nom_ < 0.001), SWA% (tau = −0.14; *p* < 0.005), SE (tau = −0.12, *p*_nom_ < 0.012), and Stage 4 (tau = −0.11; *p*_nom_ < 0.027) all contributed positively to speed (i.e., negatively to Response Time). For Accuracy a nominally significant contribution was identified for NAW (tau = −0.15; *p*_nom_ < 0.0013) and REM (tau = 0.11; *p*_nom_ < 0.017), i.e., worse accuracy with more awakenings and better accuracy with longer REM sleep (Figure 7B, Supplemental Figure 2, Supplemental Table 11).

When analyzed separately for men controlling for age, 10 correlations between PSG variables and factor scores were nominally significant, but none survived FDR correction. Nominal significant associations were observed for Response Time and SE, Stage 4, TST, SFA, SWA and SWS, for Accuracy and NAW and SWA% and for self-reported mood and arousal state and NAW and SWA% (Supplemental Figure 3).

When analyzed separately for women controlling for age, five correlations between PSG variables and factor scores were nominally significant, but none survived FDR correction. Response Time correlated significantly with SWA and SWA%; Accuracy with REM duration and Visual Perceptual sensitivity correlated with Total Sleep Time and Sleep Efficiency. For the associations between PSG variables and Response Time and Accuracy, no substantial differences between men and women were observed (Supplemental Figure 3, Supplemental Table 12). For negMood/Arousal, NAW had a larger effect in men than in women. The direction of the association between LPS and Visual Perceptual sensitivity was significantly different in men and women.

When analyzed separately for the group of young participants, while controlling for age and sex, no nominally significant correlations were observed. For the middle-aged group five nominally significant correlations were observed. These were between Response Time and SWS, SWA, and Stage 4, negMood/Arousal and SFA% and SWA%. In the older participants, two nominally significant correlations were observed between Accuracy and NAW and TST. Comparison of the associations between PSG variables and factor scores across the three age groups revealed no substantial differences for negMood/Arousal (Supplemental Figure 4, Supplemental Table 13). For Response Time, Stage 4 and SWS contributed more to speed in the middle-aged participants than in the young and older participants. The contribution of PSG variables to Accuracy was not markedly different across the age groups (except for one difference for stage 1). Visual Perceptual Sensitivity was more strongly associated with stage 4 and SWS in the older participants than in the middle-aged participants (see Supplemental Figure 5).

### Self-reported sleep measures and performance factors

Out of a total of 16 correlations between self-reported sleep measures and performance factors, six were nominally significant and all survived FDR correction. After controlling for sex but not age, Quality of Sleep (sQoS) correlated negatively with negMood/Arousal (tau = −0.135; *p*_nom_ < 0.004; *p*_FDR_ < 0.05) and positively with Accuracy (tau = 0.135; *p*_nom_ < 0.0041; *p*_FDR_ < 0.05). Feeling Refreshed upon Awakening correlated negatively with negMood/Arousal (tau = −0.165; *p*_nom_ < 0.00043; *p*_FDR_ < 0.05), positively with Accuracy (tau = 0.162; *p*_nom_ < 0.00056; *p*_FDR_ < 0.05) and with Response Time (tau = 0.136; *p*_nom_ < 0.0038; *p*_FDR_ < 0.05). Self-reported sleep latency correlated positively with negMood/Arousal (tau = 0.1515; *p*_nom_ < 0.0012; *p*_FDR_ < 0.05) (Supplemental Figure 5). Self-reported Number of Awakenings did not correlate significantly with any of the performance factors. That is, participants who felt they had fallen asleep more quickly, slept better and woke feeling refreshed, and also performed and felt better the next day.

When both age and sex were statistically controlled, six of a total of 16 correlations between self-reported sleep measures and performance factors were nominally significant, five of which survived FDR correction (Supplemental Figure 6). sQoS correlated negatively with negMood/Arousal (tau = −0.140; *p*_nom_ < 0.0029; *p*_FDR_ < 0.05) and positively with Accuracy (tau = 0.133; *p*_nom_ < 0.0046; *p*_FDR_ < 0.05). In other words, when people felt they had had a good night's sleep they reported less negative mood and were more accurate throughout the subsequent day. Refreshed upon awakening correlated negatively with negMood/Arousal (tau = −0.157; *p*_nom_ < 0.0008; *p*_FDR_ < 0.05); and positively with Accuracy (tau = 0.170; *p*_nom_ < 0.0003; *p*_FDR_ < 0.05). Self-reported sleep latency only correlated positively with negMood/Arousal (tau = 0.144; *p*_nom_ < 0.0022; *p*_FDR_ < 0.05). Self-reported NAW was not significantly correlated with any of the factor scores (Supplemental Figure 6).

When analyzed separately for men, and controlling for age, six correlations between subjective sleep measures and factor scores were nominally significant, of which 2 survived FDR correction (Supplemental Table 14). These were the correlations between Waking Refreshed and negMood/Arousal (tau = −0.240; *p*_nom_ < 0.001 and between sQoS and negMood/Arousal (tau = −0.239; *p*_nom_ < 0.001). Nominally significant associations were observed for Refreshed with Accuracy and Perceptual Sensitivity and for Subjective Latency to Sleep onset with negMood/Arousal and Perceptual Sensitivity.

In the equivalent analyses for women, controlling for age, four correlations between self-reported sleep variables and factor scores were nominally significant of which one, the correlation between Waking Refreshed and Accuracy, survived FDR correction at p < 0.1 but not at FDR correction at *p* < 0.05. The other three correlations were between Quality of Sleep and Speed and Accuracy, and between Refreshed and Speed.

When analyzed separately for young participants, while controlling for age and sex, no nominally significant correlations between subjective sleep measures and factor scores were observed (Supplemental Table 15). For the middle-aged participants three nominally significant correlations were observed of which the correlation between Refreshed and Response Time survived FDR correction at *p* < 0.05 (i.e., middle aged people who woke feeling refreshed had longer response times). The other two correlations were between Waking Refreshed and Accuracy and Subjective Sleep Latency and negMood/Arousal. In the older participants, four nominally significant correlations were observed but none survived FDR correction. Refreshed correlated with negMood/Arousal and Accuracy, Quality of Sleep with negMood/Arousal and Subjective latency to sleep with Perceptual sensitivity.

Together these analyses demonstrate that subjective aspects of sleep have consistent associations with objectively measured daytime functioning, albeit moderated by age and sex.

## Discussion

### General comments

In this study, associations between polysomnographic measures of sleep and measures of subjective sleep quality and many measures of mood, performance, and cognition were quantified in men and women across a wide age range. This simultaneous evaluation of many variables in conjunction with the statistical control for age and sex effects allowed for a quantitative comparison of the age and sex independent contribution of various sleep parameters to a variety of “dependent” variables. Application of factor analysis to the many performance measures identified four latent “performance” variables (negMood/Arousal, Response Time, Accuracy, and Visual Perceptual Sensitivity) which are likely to represent fundamental aspects of brain function and its association with EEG sleep. This approach contrasts with many previous studies in which often only one aspect of sleep (e.g., SWS, subjective sleep duration, or actigraphically assessed sleep fragmentation) or only few aspects of waking function were considered and statistical control for age and sex effects was not always applied. Nevertheless, the current data replicate several aspects of previous studies but also highlight new associations between EEG sleep and waking function. Most strikingly, the current data highlight the positive contribution of sleep continuity and REM sleep to both Subjective Sleep Quality and performance Accuracy, whereas the role of SWS is limited to Response Time. The role of SWS is not as dominant as may have been expected based on the common notion that SWS is “the deepest” or most “restorative” stage of sleep, but they are consistent with studies which have experimentally increased [e.g., (37)] or decreased (9, 26) SWS which resulted in relatively slight effects on daytime functioning. The important roles of REM sleep and sleep continuity are broadly in line with previous and recent reports (see below for references). The current data provide new insights into the associations of specific aspects of sleep with specific aspects of waking performance across the healthy adult life span and imply that attempts to improve sleep may also focus on sleep continuity and REM sleep.

### Aging and the objective and subjective quality of sleep

The current data confirm that reductions in sleep efficiency, and Slow Wave Sleep measures, including slow wave activity expressed as a percentage of total EEG power, are among the largest objective EEG based changes across the adult healthy life span. These data and the observed smaller changes in REM sleep and sleep continuity are in accordance with and extend previous polysomnographic studies [e.g., (38–40)].

The observed contribution of sleep continuity and REM sleep, but not of Slow Wave Sleep related measures including SWA and SWA%, to self-reported sleep quality and refreshed upon awakening are broadly in accordance with recent large studies in older individuals (41–43) and earlier studies [e.g., (44)]. Sleep continuity as a determinant of perceived sleep quality has also been identified in actigraphy studies [e.g., (45)] although obviously this methodology does not allow for the identification of the contribution of SWS or REM sleep. Thus it appears that from a subjective, i.e., self-reported perspective, sleep continuity and REM sleep are significant contributors to a good night's sleep.

### Aging and waking performance

The reduced drowsiness and tiredness presented here is in accordance with the previously reported longer daytime sleep latencies with aging in a sub-set of the current data (26). Indeed, when analyzed over the entire data set, objective daytime sleepiness, as assessed by the Multiple Sleep Latency Test (MSLT), and subjective sleepiness, as assessed by the KSS, across the day decrease with age (Supplemental Figure 7). Thus despite an age-related worsening of objective sleep, the daytime subjective measures of mood, alertness and affect as measured by the LARS and PANAS do not decline but rather improve in healthy aging, as reported by others (46–49).

Our waking performance data confirm large age-related changes in particular performance domains such as processing speed/reaction times on tasks such as the DSST, Lexical Decision Time and Simple Reaction Time Task as well as Sequence and Motor Control but much smaller changes in tests assessing accuracy (50, 51) and verbal fluency (52–54). Working memory and executive function as assessed from the difference between 2 and 1 N-back, the GNTCORB and PVSAT also deteriorated with age although these effects were smaller than the effects on measures of speed. Age related reductions in verbal fluency were even smaller. This may be somewhat surprising since Verbal Fluency is often regarded as an executive task. However, the task clearly depends on verbal competence and verbal/crystallized intelligence. Typically such measures favor or are associated with relatively age-resilient characteristics of performance. Verbal Fluency, as we implemented it, requires generating members of a single category for 30 s, rather than generating members of several categories. The later, but not our version, requires inhibition, which is part of the Executive domain.

In general these results support the notion that age-related changes in cognition are cognitive domain dependent (55).

The analyses of the latent variables negMood/Arousal, Response Time, Accuracy are in accordance with the patterns observed for the individual performance tests. While we recognize that the labeling of these factors is somewhat arbitrary, their age-related changes confirm the notion that with age Speed is reduced, while Accuracy is maintained even though aspects of Working Memory and Executive Function also decline with age.

### EEG sleep and waking performance

What constitutes a good night of sleep from a waking performance perspective is rarely investigated in a larger data set. The significant associations between speed related performance measures, as well as measures of executive function, with Slow Wave Sleep measures in the current analyses (not controlling for age) are not surprising in view of the strong age-related changes in all of these measures. Since after controlling for age and sex, significant association between SWS measures and speed measures persisted, whereas those with executive function became insignificant, it may be concluded that SWS and Speed do not just decline in parallel with age but that SWS actually contributes significantly to speed but not as much to executive function measures. For both sleep continuity and REM sleep the age-related change was large but the age-related change in goal-neglect was only small to medium. The significant associations between measures of sleep continuity and REM sleep with performance on the goal neglect task which emerged in the age and sex controlled analyses therefore point to an age independent contribution of these sleep parameters to executive function.

The identification of latent factors through factor analysis confirmed the large age-related increases in Response Time, i.e., a reduction of “Speed,” and this factor, after correction for sex and age, correlated with Slow Wave Sleep measures such that with more SWS speed was better preserved in aging. The factor Accuracy, which consisted primarily of accuracy measures of working memory and executive function tasks, did not change much with age. Accuracy correlated negatively with sleep continuity and positively with REM sleep such that in older people who maintained sleep continuity and REM sleep, Accuracy was better. One interesting aspect of these correlations is that although SWS correlates positively with sleep continuity, SWS and sleep continuity appear to contribute to different aspects of brain function. The negative correlation between SE (which in essence measures Wake after sleep onset) and Response Time, combined with the absence of a strong correlation between response time and NAW, further indicates that SE and sleep continuity contribute differentially to brain function.

This contribution of sleep continuity and sleep efficiency to performance measures is in accordance with studies in which sleep was assessed by actigraphy [e.g., (56)] and EEG sleep studies [e.g., (21)].

Likewise a positive contribution of REM sleep to cognition has been previously reported [for a comprehensive review see (10)]. In fact, in their pioneering study Feinberg and colleagues reported a positive association between REM sleep and REM density and cognitive performance assessed by the Wechsler Adult intelligence Scale and the Wechsler Memory Scale and did not observe a contribution of stage 4 of NREM sleep in the normal aged participants (57). More recently more REM sleep was shown to be associated with less cognitive decline as assessed by the Trails B Test and a Modified Mini-Mental State Examination in a longitudinal study in healthy older individuals (58). Most recently, a longitudinal study of cognitive decline showed that more REM sleep, but not SWS, was protective against the emergence of dementia (59). It should be noted that in several of these studies measures of sleep fragmentation also correlated with cognitive decline.

It is well established that sleep continuity declines with age and it has been reported in a separate sample that this decline occurs almost exclusively in NREM sleep, and not in REM sleep (60). This suggests that the contributions of sleep continuity and REM sleep to waking performance are to some extent independent. However, few analyses of the contribution of REM continuity to waking functions are available. Furthermore, REM sleep is usually, as in this analysis, treated as a uniform state. Further analyses of the contribution of tonic vs. phasic REM and REM sleep specific events, such as Rapid Eye Movements, to the association between REM sleep and cognition are warranted.

It is also of note that in the current analyses the contribution of sigma activity, which to a large extent reflects sleep spindles (32), to performance was very limited. This is somewhat surprising in view of the reported associations between spindle activity and declarative and procedural memory consolidation and intelligence (61) as well as verbal learning, visual attention and verbal fluency (18). However, in the current analyses, overnight memory consolidation was not considered, and it remains unclear how effects of sleep on memory consolidation relate to other aspects of brain function. In addition, in parts of the sleep spindle literature [e.g., (62)], a distinction is made between fast and slow spindles, a distinction not made in the current analyses.

### Subjective sleep measures as predictors of performance

Some of the subjective sleep measures, and in particular quality of sleep and refreshed upon awakening, were also associated with some of the performance measure as quantified by the factor scores. However, self-reported number of awakenings was not. This may indicate that sQoS and sRuA are to some extent a report of how well participants feel in the morning, rather than reflecting the “trait” quality of their sleep, because objective NAW was one of the best objective sleep quality predictors of performance. It is also possible that some objective arousals are not remembered, if “noticed,” and thus sRuA is at best, a noisy measure of disrupted sleep.

### Differences between men and women, young, middle-aged, and older participants

In accordance with previous reports SWA was higher in women than in men and in terms of sleep continuity, women slept “better” than men (63). The observed interaction between sex and age indicate that from an EEG perspective sleep may be better conserved in women, particularly after middle age. The observation that the associations between objective and subjective sleep were much stronger in women than in men is of interest because women in general have more sleep complaints than men, although from an objective sleep perspective women's sleep may be better. The stronger associations between objective and subjective sleep quality in women implies that women's perception of sleep is more in tune with sleep physiology. Nevertheless, no major sex differences in the association between objective sleep and performance as quantified by the factor analysis were identified.

Across age groups the associations between objective sleep parameters and subjective sleep quality was fairly stable except that a higher SWA% was a negative indicator of sleep quality for younger participants, and a positive indicator in older participants. Whether this phenomenon is related to changes in sleep inertia, which is thought to be positively related to SWS and may have a negative impact of how people feel, shortly after awakening, remains subject of speculation.

Associations between PSG parameters and performance factors did not change considerably across the age groups except for Slow Wave Sleep related measures and Response time for which the sign of the association was not consistent across age groups.

### Associations between sleep and cognition: comparison with sleep deprivation studies

Effects of aging on cognition have been considered to be similar to the effects of acute sleep deprivation (64) and it has been posited that sleep deprivation and aging have both large effects on executive function. In view of the highly reproducible increase in SWS following extended duration of wakefulness (65, 66) and the marked age-related decline in SWS (26), it is not surprising that sleep deprivation related effects and age-related effects on brain function were either implicitly or explicitly attributed to loss of SWS. Meta analyses and larger individual studies have shown that total sleep deprivation, SWS disruption as well as sleep restriction (which does not induce a loss of SWS) all have larger effect sizes for sustained attention than executive function (6). The current analyses and previous association studies which most likely reflect long term effects of sleep on brain function, challenge the notion that age-related changes in SWS are a main driver of decline in executive function and instead highlight a potential role for sleep continuity and REM sleep. Unfortunately studies aimed at assessing the impact of improving sleep continuity or increasing REM sleep on brain function are not available.

### Limitations of the study

This was a cross-sectional study in carefully screened healthy individuals. Although the data are relevant to our understanding of healthy aging they do not identify individual trajectories and may be subject to cohort effects. Furthermore, how these data relate to the associations between sleep disturbances and cognitive decline in mild cognitive impairment and the various dementias remains debatable. Nevertheless, the importance of both sleep continuity and REM sleep has been emphasized in studies in these patient groups. Although we used a wide range of performance measures, measures of procedural or declarative memory and their putative sleep dependent consolidation were not included. A more prominent role for SWS and sleep spindles might have been detected had we not only included a procedural memory task (Serial Reaction Time) but also included measures of episodic memory or motor learning including overnight consolidation of motor skills.

We used Factor Analyses to provide a description of changes in cognition beyond those captured by particular tasks, which may often describe effects on the task rather than effects on cognitive domains. The factor structure described here is similar to the factor structure we obtained using a similar test battery in an experiment assessing the effects of circadian rhythmicity on cognition (67). Thus while we can be confident about the factor structure, we cannot determine to what extent the observed associations between sleep and factor scores are dependent on the specific sample of participants and the current experimental setting.

In the current analysis we focussed on average performance during the day, which we consider to be a relevant estimate of waking function in general. By doing so, however, we are not seeking to deny the possibility that there are time of day specific associations between performance and nocturnal sleep.

We have only analyzed one EEG derivation and it may be that more associations between for example SWA or SFA and brain function would have emerged had we analyzed a frontal rather than a central derivation or applied a topographical sleep EEG analysis using high density EEG. However, some of the association, such as those relating to sleep continuity and REM sleep are likely to be independent of topography and age-related changes in EEG amplitude are observed in all EEG derivations (68).

Our main conclusions are based on cross sectional associations between sleep and cognition that persisted after controlling for age and sex. This approach robustly identifies age and sex independent contributions of sleep to brain function but may underestimate the contribution of sleep. We have assiduously corrected for false discovery and can be therefore confident that the number of false positive results are limited. However, this conservative approach may have led to dismissing associations with a considerable effect size as being unimportant. In addition, we only have analyzed sleep variables separately. We did so because our primary aim was to identify robust predictors and directly compare the relative strength of associations between sleep and performance across sleep parameters. In the current analyses we did not aim to understand the interrelationship between the various sleep parameters. It may be possible that combinations of sleep parameters yield better predictors and better capture the aspects of the sleep process contribution to specific aspects of waking performance but we considered this to be beyond the scope of the current analyses.

Likewise, we have treated the sleep period as a single whole entity. Perhaps some aspects of sleep, early SWS, late REM, the dynamics of the sleep dependent decline of SWS or increase of REM sleep may be more predictive than a whole night summary measure. Similarly, we average performance across the whole day. Even though this undoubtedly provides a good measure of average waking performance (although the performance battery was not administered in the evening hours) it may be that that some objective sleep measure predict particular performance impairments in the morning, afternoon or whenever.

Finally we tacitly assume that it is sleep that influences brain/function and cognition but we cannot exclude that the direction of causality is reversed, i.e., that better accuracy leads to more REM sleep, etc.

### Implications

Using conservative statistical approaches we identify a contribution of sleep continuity and REM sleep to subjective sleep quality which is much stronger in women than in men. This implies that improving REM sleep and sleep continuity should be targets for improving the subjective experience of sleep, in particular in women. We also identify a contribution of SWS to response time and of sleep continuity and REM sleep to Accuracy implying that particular cognitive deficits may be countered by specific sleep interventions.

## Author contributions

CdM curated the data, prepared figures and tables and contributed to drafting the manuscript. SJ was responsible for coding and running of all statistical analyses programs. GA assisted with preparation of data for analysis. JG constructed the test battery, conceived some of the analyses, interpreted the cognitive results, and contributed to the writing of the manuscript. D-JD conceived the main analyses, drafted the manuscript, and oversaw the study.

### Conflict of interest statement

The authors declare that the research was conducted in the absence of any commercial or financial relationships that could be construed as a potential conflict of interest.

## Footnotes

^1^Throughout we refer to contrasts between biologically male and female research participants by the term sex difference, rather than the term gender difference, which connotes the assignment of, or preference for, different social roles.

## Supplementary material

Supplemental Table 1 Sample distribution by age groups and sex.

Supplemental Table 2 EEG sleep variables by age groups and sex, mean and standard error.

Supplemental Table 3 Polysomnographical sleep variables by age and sex in the total sample.

Supplemental Table 4 Self-reported sleep variables by age and sex in the total sample.

Supplemental Table 5 Kendall correlations between polysomnographical and self-reported sleep variables controlled for age and sex.

Supplemental Table 6 Computer-based task, measures details.

Supplemental Table 7 Cognitive performance variables by age and sex in the total sample.

Supplemental Table 8 Correlation (Kendall's tau) between PSG variables and cognition variables controlling for sex.

Supplemental Table 9 Correlation (Kendall's tau) between PSG variables and cognition variables controlling for age and sex.

Supplemental Table 10 Correlation between PSG variables and cognition factors controlling for sex.

Supplemental Table 11 Correlation between PSG variables and cognition factors controlling for age and sex.

Supplemental Table 12 Correlation between PSG variables and cognition factors by sex controlling for age.

Supplemental Table 13 Correlation between PSG variables and cognition factors by age groups controlling for sex and age.

Supplemental Table 14 Correlation between self-reported sleep variables and cognition factors by sex controlling for age.

Supplemental Table 15 Correlation between self-reported sleep variables and cognition factors by age groups controlling for sex and age.

Supplemental Figure 1 Association between EEG sleep parameters and subjective assessment of refreshed upon awakening: **(A)** data controlled for sex and age; **(B)** data controlled for age and shown separately for men (gray bars) and women (red bars); **(C)** data controlled for age and sex and shown separately for three age groups: Young, 20–30 years (yellow bars); Middle-aged, 31–64 years (light green bars); and Older, 65–84 years (dark green bars). For reference, horizontal lines indicate the corresponding Cohen's *d* effect size: S, small, *d* = 0.2; M, medium, *d* = 0.5; H, high, *d* = 0.8. ^*^ indicate significant effects following FDR (False-Discovery Rate) correction (*p* < 0.05). § indicates a significant (*p* < 0.05) difference between men and women Kendall's Tau-values. ○ indicates a significant (*p* < 0.05) difference between middle-aged and older Kendall's Tau-values. LPS, latency to persistent sleep (min); TST, total sleep time (min); SE, sleep efficiency (%); NAW, number of awakenings; REM, rapid eye movement; Stage 1, duration of stage 1 sleep (min); Stage 2, duration of stage 2 sleep (min); Stage 4, duration of stage 4 sleep (min); SWS, slow wave sleep; SWA, slow wave activity (μV^2^); SWA%, slow wave activity in percentage of total power; SFA, sigma activity (μV^2^); SFA%, sigma activity in percentage of total power.

Supplemental Figure 2 Association between EEG sleep parameters and performance factors controlled for age and sex: **(A)** negMood/Arousal (Factor 1); **(B)** Response time (Factor 2); **(C)** Accuracy (Factor 3); **(D)** Visual-Perceptual Sensitivity (Factor 4). For reference, horizontal lines indicate the corresponding Cohen's *d* effect size: S, small, *d* = 0.2; M, medium, *d* = 0.5; H, high, *d* = 0.8. ^*^ indicate significant effects (*p* < 0.05). LPS, latency to persistent sleep (min); TST, total sleep time (min); SE, sleep efficiency (%); NAW, number of awakenings; REM, rapid eye movement; Stage 1, duration of stage 1 sleep (min); Stage 2, duration of stage 2 sleep (min); Stage 4, duration of stage 4 sleep (min); SWS, slow wave sleep; SWA, slow wave activity (μV^2^); SWA%, slow wave activity in percentage of total power; SFA, sigma activity (μV^2^); SFA%, sigma activity in percentage of total power.

Supplemental Figure 3 Association between EEG sleep parameters and performance factors shown separately for men (gray bars) and women (red bars): **(A)** negMood/Arousal (Factor 1); **(B)** Response time (Factor 2); **(C)** Accuracy (Factor 3); **(D)** Visual-Perceptual Sensitivity (Factor 4). For reference, horizontal lines indicate the corresponding Cohen's *d* effect size: S, small, *d* = 0.2; M, medium, *d* = 0.5; H, high, *d* = 0.8. ^*^ indicate significant effects (*p* < 0.05). § indicates a significant (*p* < 0.05) difference between men and women Kendall's Tau-values. LPS, latency to persistent sleep (min); TST, total sleep time (min); SE, sleep efficiency (%); NAW, number of awakenings; REM, rapid eye movement; Stage 1, duration of stage 1 sleep (min); Stage 2, duration of stage 2 sleep (min); Stage 4, duration of stage 4 sleep (min); SWS, slow wave sleep; SWA, slow wave activity (μV^2^); SWA%, slow wave activity in percentage of total power; SFA, sigma activity (μV^2^); SFA%, sigma activity in percentage of total power.

Supplemental Figure 4 Association between EEG sleep parameters and performance factors controlled for age shown separately for three age groups: Young, 20–30 years (yellow bars); Middle-aged, 31–64 years (light green bars); and Older, 65–84 years (dark green bars): **(A)** negMood/Arousal (Factor 1); **(B)** Response time (Factor 2); **(C)** Accuracy (Factor 3); **(D)** Visual-Perceptual Sensitivity (Factor 4). For reference, horizontal lines indicate the corresponding Cohen's *d* effect size: S, small, *d* = 0.2; M, medium, *d* = 0.5; H, high, *d* = 0.8. ^*^ indicate significant effects (*p* < 0.05). § indicates significant (*p* < 0.05) differences between men vs. women Kendall's Tau-values. ∙ indicates significant (*p* < 0.05) differences between young vs. older Kendall's Tau-values. Indicates significant (*p* < 0.05) differences between middle-aged vs. older Kendall's Tau-values. Φ indicates significant (*p* < 0.05) differences between middle-aged and young Kendall's Tau-values. LPS, latency to persistent sleep (min); TST, total sleep time (min); SE, sleep efficiency (%); NAW, number of awakenings; REM, rapid eye movement; Stage 1, duration of stage 1 sleep (min); Stage 2, duration of stage 2 sleep (min); Stage 4, duration of stage 4 sleep (min); SWS, slow wave sleep; SWA, slow wave activity (μV^2^); SWA%, slow wave activity in percentage of total power; SFA, sigma activity (μV^2^); SFA%, sigma activity in percentage of total power.

Supplemental Figure 5 Association between self-reported sleep parameters and performance factors controlled for sex: **(A)** negMood/Arousal (Factor 1); **(B)** Response time (Factor 2); **(C)** Accuracy (Factor 3); **(D)** Visual-Perceptual Sensitivity (Factor 4). For reference, horizontal lines indicate the corresponding Cohen's *d* effect size: S, small, *d* = 0.2; M, medium, *d* = 0.5; H, high, *d* = 0.8. ^*^ indicate significant effects following FDR (False-Discovery Rate) correction (*p* < 0.05). sRuA, subjective assessment of refreshed upon awakening; sSleep-Lat, subjective sleep latency; sNAW, subjective number of awakenings; sQoS, subjective quality of sleep.

Supplemental Figure 6 Association between self-reported sleep parameters and performance factors controlled for age and sex: **(A)** negMood/Arousal (Factor 1); **(B)** Response time (Factor 2); **(C)** Accuracy (Factor 3); **(D)** Visual-Perceptual Sensitivity (Factor 4). For reference, horizontal lines indicate the corresponding Cohen's *d* effect size: S, small, *d* = 0.2; M, medium, *d* = 0.5; H, high, *d* = 0.8. ^*^ indicate significant effects following FDR (False-Discovery Rate) correction (*p* < 0.05). sRuA, subjective assessment of refreshed upon awakening; sSleep-Lat, subjective sleep latency; sNAW, subjective number of awakenings; sQoS, subjective quality of sleep.

Supplemental Figure 7 KSS & MSLT. Age-related changes combined for males and females (LSmean ± Std Error) in: **(A)** daytime subjective sleepiness [Karolinska Sleepiness Score (KSS)]; **(B)** daytime sleep latency [Multiple Sleep Latency Test (MSLT)]. For the KSS there were no statistically significant effects. For MSLT, there was a significant effect of age [*F* = 9.54, (5, 194), *p* < 0.0001]; *post-hoc* tests revealed: 20–29 < 40–49 (*p* = 0.001), 50–59 (*p* = 0.0004), 60–69 (*p* < 0.0001), 70–84 (*p* < 0.0001), 30–39 < 40–49 (*p* = 0.0346), 50–59 (*p* = 0.0142), 60–69 (*p* = 0.0016), 70–84 (*p* < 0.0001), 40–49 < 70–84 (*p* = 0.0309).
