# Supplementary material for: Rapid Eye Movement Sleep, Sleep Continuity and Slow Wave Sleep as Predictors of Cognition, Mood, and Subjective Sleep Quality in Healthy Men and Women, Aged 20–84 Years
Source: Front Psychiatry. 2018 Jun 22;9:255. doi: 10.3389/fpsyt.2018.00255 (PMC6024010; doi:10.3389/fpsyt.2018.00255)
Supplement: Supplemental Table 1 — Sample distribution by age groups and sex. [file Table_1.DOCX]

**Supplemental Table 1.** Sample distribution by age groups and sex.

|  | **Age group** | | | |
| --- | --- | --- | --- | --- |
| **Sex** | *Young (%)* | *Middle-aged (%)* | *Older (%)* | *Total sample (%)* |
| *M* | 37 (18.0) | 35 (17.0) | 20 (9.7) | 92 (44.7) |
| *F* | 29 (14.1) | 41 (19.9) | 44 (21.4) | 114 (55.3) |
| *Tot* | 66 (32.0) | 76 (36.9) | 64 (31.1) | 206 (100.0) |

**Note.** Age groups: young (20–30 years), middle-aged (31–64 years), older (65–84 years). F, females; M, males; Tot., total.
