# Supplementary material for: Rapid Eye Movement Sleep, Sleep Continuity and Slow Wave Sleep as Predictors of Cognition, Mood, and Subjective Sleep Quality in Healthy Men and Women, Aged 20–84 Years
Source: Front Psychiatry. 2018 Jun 22;9:255. doi: 10.3389/fpsyt.2018.00255 (PMC6024010; doi:10.3389/fpsyt.2018.00255)
Supplement: Supplemental Table 2 — EEG sleep variables by age groups and sex, mean and standard error. [file Table_2.DOCX]

**Supplemental Table 2.** EEG sleep variables by age groups and sex, mean and standard error.

|  |  |  | **Age group** | | | | | | | | | | |
| --- | --- | --- | --- | --- | --- | --- | --- | --- | --- | --- | --- | --- | --- |
| **Sleep** |  |  | Young | |  | Middle-aged | |  | Older | |  | Total sample | |
| **Variable** | **Sex** |  | *Mean* | *SEM* |  | *Mean* | *SEM* |  | *Mean* | *SEM* |  | *Mean* | *SEM* |
| LPS | *M* |  | 17.529 | 2.553 |  | 18.273 | 2.068 |  | 17.605 | 2.666 |  | 17.828 | 1.402 |
| (min) | *F* |  | 20.966 | 3.360 |  | 15.737 | 2.268 |  | 18.830 | 3.382 |  | 18.283 | 1.762 |
|  | *Tot* |  | 19.086 | 2.060 |  | 16.884 | 1.552 |  | 18.460 | 2.483 |  | 18.085 | 1.165 |
| TST | *M* |  | 429.273 | 7.282 |  | 402.307 | 7.030 |  | 384.741 | 9.044 |  | 409.319 | 4.771 |
| (min) | *F* |  | 427.806 | 8.559 |  | 420.748 | 5.669 |  | 400.209 | 5.910 |  | 414.562 | 3.889 |
|  | *Tot* |  | 428.608 | 5.515 |  | 412.412 | 4.543 |  | 395.544 | 4.989 |  | 412.281 | 3.020 |
| SE | *M* |  | 91.261 | 1.091 |  | 84.732 | 1.337 |  | 80.209 | 1.881 |  | 86.371 | 0.907 |
| (%) | *F* |  | 92.553 | 0.748 |  | 88.440 | 1.097 |  | 83.844 | 1.227 |  | 87.706 | 0.719 |
|  | *Tot* |  | 91.846 | 0.686 |  | 86.764 | 0.874 |  | 82.748 | 1.041 |  | 87.125 | 0.567 |
| NAW | *M* |  | 18.600 | 1.414 |  | 16.850 | 1.603 |  | 26.530 | 3.133 |  | 19.670 | 1.135 |
| (n) | *F* |  | 17.660 | 1.567 |  | 18.200 | 1.111 |  | 20.930 | 1.418 |  | 19.120 | 0.793 |
|  | *Tot* |  | 18.170 | 1.043 |  | 17.590 | 0.943 |  | 22.620 | 1.393 |  | 19.360 | 0.666 |
| REM | *M* |  | 90.669 | 3.884 |  | 91.559 | 4.124 |  | 78.122 | 5.984 |  | 88.267 | 2.602 |
| (min) | *F* |  | 85.499 | 3.964 |  | 98.853 | 4.716 |  | 81.754 | 3.431 |  | 88.768 | 2.455 |
|  | *Tot* |  | 88.327 | 2.779 |  | 95.556 | 3.194 |  | 80.659 | 2.980 |  | 88.550 | 1.786 |
| Stage 1 | *M* |  | 28.179 | 2.266 |  | 27.955 | 1.904 |  | 40.954 | 3.560 |  | 30.884 | 1.496 |
| (min) | *F* |  | 27.398 | 3.144 |  | 27.690 | 2.567 |  | 22.680 | 1.341 |  | 25.664 | 1.329 |
|  | *Tot* |  | 27.825 | 1.873 |  | 27.810 | 1.639 |  | 28.191 | 1.765 |  | 27.935 | 1.008 |
| Stage 2 | *M* |  | 196.457 | 5.757 |  | 202.421 | 5.265 |  | 214.223 | 8.644 |  | 202.599 | 3.622 |
| (min) | *F* |  | 200.186 | 5.883 |  | 202.241 | 5.838 |  | 200.097 | 5.945 |  | 200.879 | 3.423 |
|  | *Tot* |  | 198.147 | 4.100 |  | 202.322 | 3.961 |  | 204.357 | 4.934 |  | 201.627 | 2.489 |
| Stage 4 | *M* |  | 74.926 | 3.426 |  | 49.316 | 4.451 |  | 17.501 | 5.284 |  | 52.671 | 3.383 |
| (min) | *F* |  | 75.201 | 3.971 |  | 58.987 | 4.146 |  | 50.912 | 4.064 |  | 60.004 | 2.534 |
|  | *Tot* |  | 75.051 | 2.577 |  | 54.615 | 3.067 |  | 40.836 | 3.773 |  | 56.814 | 2.064 |
| SWS | *M* |  | 113.967 | 4.359 |  | 80.372 | 4.214 |  | 51.442 | 6.318 |  | 87.569 | 3.762 |
| (min) | *F* |  | 114.722 | 6.574 |  | 91.964 | 4.270 |  | 95.678 | 4.761 |  | 99.251 | 3.028 |
|  | *Tot* |  | 114.309 | 3.783 |  | 86.724 | 3.073 |  | 82.337 | 4.596 |  | 94.169 | 2.397 |
| SWA | *M* |  | 2934.376 | 227.625 |  | 1742.552 | 134.734 |  | 1049.370 | 125.947 |  | 2147.733 | 141.748 |
| (µV^2^) | *F* |  | 3574.056 | 302.117 |  | 2470.100 | 142.428 |  | 2115.385 | 126.215 |  | 2625.884 | 119.546 |
|  | *Tot* |  | 3207.026 | 186.344 |  | 2144.332 | 108.005 |  | 1822.754 | 118.236 |  | 2414.856 | 92.968 |
| SWA% | *M* |  | 65.045 | 0.964 |  | 63.204 | 0.661 |  | 56.332 | 1.500 |  | 62.802 | 0.657 |
|  | *F* |  | 67.422 | 0.664 |  | 63.842 | 0.804 |  | 60.359 | 0.835 |  | 63.484 | 0.536 |
|  | *Tot* |  | 66.058 | 0.635 |  | 63.556 | 0.531 |  | 59.254 | 0.767 |  | 63.183 | 0.417 |
| SFA | *M* |  | 64.623 | 5.341 |  | 54.601 | 7.088 |  | 37.004 | 3.583 |  | 55.923 | 3.773 |
| (µV^2^) | *F* |  | 80.872 | 8.568 |  | 63.341 | 5.171 |  | 51.883 | 3.248 |  | 63.660 | 3.337 |
|  | *Tot* |  | 71.549 | 4.836 |  | 59.428 | 4.269 |  | 47.798 | 2.704 |  | 60.245 | 2.509 |
| SFA% | *M* |  | 1.585 | 0.146 |  | 1.981 | 0.187 |  | 2.229 | 0.256 |  | 1.850 | 0.109 |
|  | *F* |  | 1.676 | 0.198 |  | 1.759 | 0.152 |  | 1.556 | 0.089 |  | 1.662 | 0.083 |
|  | *Tot* |  | 1.624 | 0.118 |  | 1.858 | 0.118 |  | 1.741 | 0.103 |  | 1.745 | 0.067 |

**Note.** LPS, latency to persistent sleep (min); TST, total sleep time (min); SE, sleep efficiency (%); NAW, number of awakenings; REM, rapid eye movement; Stage 1, duration of stage 1 sleep (min); Stage 2, duration of stage 2 sleep (min); Stage 4, duration of stage 4 sleep (min); SWS, slow wave sleep; SWA, slow wave activity (µV^2^); SWA%, slow wave activity in percentage of total power; SFA, sigma activity (µV^2^); SFA%, sigma activity in percentage of total power. For SWA, SWA%, SFA and SFA% n = 179 observations were included in the analysis. For all remaining variables, n = 200 observations were included.
