# Supplementary material for: Rapid Eye Movement Sleep, Sleep Continuity and Slow Wave Sleep as Predictors of Cognition, Mood, and Subjective Sleep Quality in Healthy Men and Women, Aged 20–84 Years
Source: Front Psychiatry. 2018 Jun 22;9:255. doi: 10.3389/fpsyt.2018.00255 (PMC6024010; doi:10.3389/fpsyt.2018.00255)
Supplement: Supplemental Table 3 — Polysomnographical sleep variables by age and sex in the total sample. [file Table_3.DOCX]

**Supplemental Table 3.** Polysomnographical sleep variables by age and sex in the total sample.

|  | **Age** | |  | **Sex** | |  | **Age x Sex** | |  | **Post-hoc contrasts** | | | | | |
| --- | --- | --- | --- | --- | --- | --- | --- | --- | --- | --- | --- | --- | --- | --- | --- |
| **Variable** | *F (df = 5)* | *P-value* |  | *F (df = 1)* | *P-value* |  | *F (df = 5)* | *P-value* |  | Age |  | | Sex |  | Age x Sex |
| LPS (min) | 0.3 | 0.9115 |  | 0.92 | 0.34 |  | 0.54 | 0.746 |  |  | |  |  |  |  |
| TST (min) | 6.79 | **<0.0001*** |  | 5.57 | **0.019** |  | 2.47 | **0.034** |  | A1 > A3, A4, A5, A6; A2 > A3, A5, A6 | |  | M < F |  | A1M > A3M, A4M, A5M, A6M; A1F > A3F, A6F; A2M > A4M, A5M, A6M; A2F > A6F; A4F > A3F, A6F, A4M; A5F > A6F |
| SE (%) | 15.3 | **<0.0001*** |  | 10.15 | **0.0017** |  | 2.65 | **0.0241** |  | A1 > A3, A4, A5, A6; A2 > A3, A4, A5, A6; A6 < A3, A4 | |  | M < F |  | A1M > A3M, A4M, A5M, A6M; A1F > A3F, A5F, A6F; A2M > A4M, A5M, A6M; A2F > A3F, A5F, A6F; A3M > A4M, A6M; A4F > A3F, A4M, A5F, A6F; A5F > A5M, A6F |
| NAW (n) | 3.35 | **0.006** |  | 0.57 | 0.4515 |  | 1.16 | 0.3318 |  | A6 < A1, A2, A3; A2 < A4 | |  |  |  |  |
| REM (min) | 3.07 | **0.011** |  | 0.72 | 0.3968 |  | 1.62 | 0.1561 |  | A1 < A2; A6 < A1, A2, A3, A5 | |  |  |  |  |
| Stage 1 (min) | 0.91 | 0.479 |  | 7.98 | **0.005** |  | 3.36 | **0.006** |  |  | |  | M > F |  | A6M > A1M, A2M, A3M, A4M, A5M, A6F |
| Stage 2 (min) | 0.54 | 0.7435 |  | 0.72 | 0.3977 |  | 1.24 | 0.2912 |  |  | |  |  |  |  |
| Stage 4 (min) | 16.61 | **<0.0001*** |  | 19.59 | **<0.0001*** |  | 2.7 | **0.022** |  | A1 > A3, A4, A5, A6; A2 > A4, A5, A6; A3 > A4, A5, A6 | |  | M < F |  | A1M > A3M, A4M, A5M, A6M; A1F > A4F, A5F, A6F; A2M > A4M, A5M, A6M; A3F > A4F, A5F, A3M; A4M < A4F; A5M < A5F; A6M < A6F, A3M |
| SWS (min) | 12.37 | **<0.0001*** |  | 23.38 | **<0.0001*** |  | 3.23 | **0.008** |  | A1 > A2, A3, A4, A5, A6; A2 > A4, A5, A6; A3 > A6 | |  | M < F |  | A1M > A2M, A3M, A4M, A5M, A6M; A1F > A4F, A5F; A2M > A4M, A5M, A6M; A5M < A5F; A6M < A3M, A6F |
| SWA (µV^2^) | 22.16 | **<0.0001*** |  | 46.42 | **<0.0001*** |  | 3.48 | **0.005** |  | A1 > A3, A4, A5, A6; A2 > A4, A5, A6; A3 > A6; A5 > A6 | |  | M < F |  | A1M < A1F; A1M > A3M, A4M, A5M, A6M; A1F > A3F, A4,F, A5F, A6F; A2M > A3M, A4M, A5M, A6M; A3F > A3M, A5F; A4M < A4F; A5M < A5F; A6M < A6F; A6M < A3M, A4M, A5M |
| SWA% | 14.56 | **<0.0001*** |  | 8.45 | **0.004** |  | 1.54 | 0.181 |  | A1 > A3, A4, A5, A6; A2 > A5, A6; A3 > A5, A6; A6 < A4, A5 | |  | M < F |  |  |
| SFA (µV^2^) | 5.02 | **0.0003*** |  | 6.78 | **0.0101** |  | 1.42 | 0.2204 |  | A1 > A4, A5, A6; A2 > A4, A5, A6; A3 > A6 | |  | M < F |  |  |
| SFA% | 0.96 | 0.446 |  | 4.62 | **0.033** |  | 1.08 | 0.373 |  |  | |  | M > F |  |  |

**Note.** LPS, latency to persistent sleep (min); TST, total sleep time (min); SE, sleep efficiency (%); NAW, number of awakenings; REM, rapid eye movement; Stage 1, duration of stage 1 sleep (min); Stage 2, duration of stage 2 sleep (min); Stage 4, duration of stage 4 sleep (min); SWS, slow wave sleep; SWA, slow wave activity (µV^2^); SWA%, slow wave activity in percentage of total power; SFA, sigma activity (µV^2^); SFA%, sigma activity in percentage of total power. Bold values indicate significant correlations p < 0.05; * indicates significance levels of 0.05 that remain following FDR (False-Discovery Rate procedure as proposed by Benjamini–Hochberg–Yekutieli) correction. Post-hoc contrasts < 0.05 are reported and the direction of the contrast is indicated. M, male; F, female; Age groups: A1 18 - 29 years; A2 30 – 39 years; A3 40 – 49 years; A4 50 – 59 years; A5 60 – 69 years; A6 > 69 years. For SWA, SWA%, SFA and SFA% n = 179 observations were included in the analysis. For all remaining variables, n = 200 observations were included.
