# Supplementary material for: Rapid Eye Movement Sleep, Sleep Continuity and Slow Wave Sleep as Predictors of Cognition, Mood, and Subjective Sleep Quality in Healthy Men and Women, Aged 20–84 Years
Source: Front Psychiatry. 2018 Jun 22;9:255. doi: 10.3389/fpsyt.2018.00255 (PMC6024010; doi:10.3389/fpsyt.2018.00255)
Supplement: Supplemental Table 4 — Self-reported sleep variables by age and sex in the total sample. [file Table_4.DOCX]

**Supplemental Table 4.** Self-reported sleep variable by age and sex in the total sample.

|  | Age | |  | Sex | |  | Age x Sex | |
| --- | --- | --- | --- | --- | --- | --- | --- | --- |
| Variable | *F (df = 5)* | *P-value* |  | *F (df = 1)* | *P-value* |  | *F (df = 5)* | *P-value* |
| sRuA | 1.21 | 0.3068 |  | 0.11 | 0.7459 |  | 0.24 | 0.9464 |
| sSleep-Lat | 0.34 | 0.8896 |  | 0.49 | 0.4859 |  | 1.23 | 0.295 |
| sNAW | 2.13 | 0.0633 |  | 0.02 | 0.8778 |  | 0.55 | 0.7397 |
| sQoS | 1.51 | 0.1878 |  | 0.22 | 0.6364 |  | 0.21 | 0.9561 |

**Note.** sRuA, refreshed upon awakening; sSleep-Lat, sleep onset latency (sec); sNAW, number of awakenings; sQoS, quality of sleep. N = 206 observations were included in the analysis.
