# Supplementary material for: Rapid Eye Movement Sleep, Sleep Continuity and Slow Wave Sleep as Predictors of Cognition, Mood, and Subjective Sleep Quality in Healthy Men and Women, Aged 20–84 Years
Source: Front Psychiatry. 2018 Jun 22;9:255. doi: 10.3389/fpsyt.2018.00255 (PMC6024010; doi:10.3389/fpsyt.2018.00255)
Supplement: Supplemental Table 5 — Kendall correlations between polysomnographical and self-reported sleep variables controlled for age and sex. [file Table_5.DOCX]

**Supplemental Table 5.** Kendall correlations between polysomnographical and self-reported sleep variables controlled for age and sex.

|  |  |  |  |  | | **Self-reported sleep variable** | | | | | | | |
| --- | --- | --- | --- | --- | --- | --- | --- | --- | --- | --- | --- | --- | --- |
|  |  | sRuA | |  | sSleep-Lat | | |  | sNAW | |  | sQoS | |
| **PSG variable** |  | τ | *p-value* |  | τ | | *p-value* |  | τ | *p-value* |  | τ | *p-value* |
| LPS (min) |  | -0.105 | **0.027** |  | 0.288 | | **0.000*** |  | 0.023 | 0.630 |  | -0.070 | 0.143 |
| TST (min) |  | 0.047 | 0.322 |  | -0.189 | | **0.000*** |  | -0.074 | 0.120 |  | 0.141 | **0.003** |
| SE (%) |  | 0.048 | 0.308 |  | -0.159 | | **0.001*** |  | -0.048 | 0.314 |  | 0.122 | **0.010** |
| NAW (n) |  | -0.196 | **0.000*** |  | 0.180 | | **0.000*** |  | 0.200 | **0.000*** |  | -0.248 | **0.000*** |
| REM (min) |  | 0.076 | 0.111 |  | -0.071 | | 0.139 |  | -0.116 | **0.014** |  | 0.190 | **0.000*** |
| Stage 1 (min) |  | -0.070 | 0.143 |  | 0.142 | | **0.003** |  | 0.086 | 0.070 |  | -0.082 | 0.084 |
| Stage 2 (min) |  | -0.031 | 0.511 |  | -0.098 | | **0.042** |  | 0.048 | 0.316 |  | 0.017 | 0.717 |
| Stage 4 (min) |  | 0.025 | 0.600 |  | -0.067 | | 0.163 |  | -0.097 | **0.040** |  | 0.063 | 0.182 |
| SWS (min) |  | 0.005 | 0.921 |  | -0.063 | | 0.193 |  | -0.043 | 0.368 |  | 0.019 | 0.682 |
| SWA (µV^2^) |  | 0.019 | 0.703 |  | 0.035 | | 0.487 |  | -0.040 | 0.431 |  | 0.026 | 0.600 |
| SWA% |  | 0.012 | 0.813 |  | 0.032 | | 0.524 |  | -0.006 | 0.903 |  | 0.004 | 0.936 |
| SFA (µV^2^) |  | 0.019 | 0.703 |  | 0.103 | | **0.043** |  | -0.042 | 0.409 |  | 0.015 | 0.765 |
| SFA% |  | 0.015 | 0.761 |  | 0.054 | | 0.284 |  | 0.021 | 0.680 |  | -0.003 | 0.951 |

**Note.** LPS, latency to persistent sleep (min); TST, total sleep time (min); SE, sleep efficiency (%); NAW, number of awakenings; REM, rapid eye movement; Stage 1, duration of stage 1 sleep (min); Stage 2, duration of stage 2 sleep (min); Stage 4, duration of stage 4 sleep (min); SWS, slow wave sleep; SWA, slow wave activity (µV^2^); SWA%, slow wave activity in percentage of total power; SFA, sigma activity (µV^2^); SFA%, sigma activity in percentage of total power; sRuA, refreshed upon awakening; sSleep-Lat, sleep onset latency (sec); sNAW, number of awakenings; sQoS, quality of sleep. Bold values indicate significant correlations; * indicates significance levels of 0.05 that remain following FDR (False-Discovery Rate procedure as proposed by Benjamini–Hochberg–Yekutieli) correction. Number of observations is as follows: 1) sSleep-Lat: n = 176 for SWA, SWA%, SFA and SFA%, n = 196 for all remaining variables; 2) sRuA, sNAW & sQoS: n = 179 for SWA, SWA%, SFA and SFA%, n = 200 for all remaining variables.
