# Supplementary material for: Rapid Eye Movement Sleep, Sleep Continuity and Slow Wave Sleep as Predictors of Cognition, Mood, and Subjective Sleep Quality in Healthy Men and Women, Aged 20–84 Years
Source: Front Psychiatry. 2018 Jun 22;9:255. doi: 10.3389/fpsyt.2018.00255 (PMC6024010; doi:10.3389/fpsyt.2018.00255)
Supplement: Supplemental Table 6 — Computer-based task, measures details. [file Table_6.DOCX]

**Supplemental Table 6.** Computer-based task, measures details.

| **Task name (acronym)** | **Definition of Measures** |
| --- | --- |
| **AFFECT AND MOOD** | |
| *Positive and Negative Affect Scales (PANAS)* | PANASPOS: mean positive affect  PANASNEG: mean negative affect |
| *Leeds Analogue Rating Scales (LARS)* | LARSSED: mean of ratings of Tired, Drowsy, Alert (reversed), Energetic (reversed)  LARSANXI: mean component Anxious  LARSCLUM: mean component Clumsy  LARSDEPR: mean component Depressed  LARSDIZZ: mean component Dizzy  LARSDROW: mean component Drowsy  LARSENER: mean component Energetic  LARSHAPP: mean component Happy  LARSRELA: mean component Relaxed  LARSSAD: mean component Sad  LARSTIRE: mean component Tired |
| **WORKING MEMORY** | |
| *Spatial N-Back (S1BK, S2BK)* | S1BKPCT: percentage of correct responses in spatial 1-back  S2BKPCT: percentage of correct responses in spatial 2-back  Derided Measure:  S1-2BKPCT: Cost of increased “executive” demand (updating,  switching, load) |
| *Verbal N-Back (V1BK; V2BK)* | V1BKPCT: percentage of correct responses in verbal 1-back  V2BKPCT: percentage of correct responses in verbal 2-back  Derived measure:  V1-2BKPCT: Cost of increased “executive” demand (updating,  switching, load) |
| **AROUSAL AND SUSTAINED ATTENTION** | |
| *Critical Flicker Fusion (CFF)* | CFFDN: descending threshold (ms)  CFFUP: ascending threshold (ms)  CFFIU: interval of uncertainty (ms)  CFFMED: median of Ascending and Descending thresholds (ms)  CFFPSE: point of subjective equality (ms) |
| *Sustained Attention to Response Task (SART)* | SARTEOC: errors of commission  SARTEOO: errors of omission  SARTACC: accuracy (d-prime) |
| *Digit Symbol Substitution Task (DSST)* | DSSTNUM: total number attempted in 40s  DSSTCOR: total number correct in 40s |
| **EXECUTIVE FUNCTION** | |
| *Goal Neglect Task (GNT)* | GNTNCOR: number of correctly reported letters  GNTCORB: percentage correct before switch  GNTCORA: percentage correct after switch |
| *Paced Visual Serial Addition (PVSAT)* | PVSAT: percentage correct of 25 consecutive additions |
| *Verbal Fluency Task (VFT)* | VFTUCI:: average number of unique correct category items  VFTECI: average number of incorrect category items |
| **SEQUENCE AND MOTOR CONTROL** | |
| *Serial Reaction Task (SERRT)* | SERRTSEQB: average time for correct responses in sequence block immediately before mid-test random block (ms)  SERRTSEQA: average time for correct responses in sequence block immediately after mid-test random block (ms)  SERRTRAN: average time for correct responses in mid-test random block (ms)  Derived measures:  SERRT (RAN-SEQB): Difference between SERRTRAN and  SERRTSEQB, providing a measure of sequence learning  SERRT (RAN-SEQA): Difference between SERRTSEQA and  SERRTRAN, providing a measure of detecting and recommencing  learned pattern  SERRT (SEQA-SEQB): Difference between SERRTSEQA and  SERRTSEQB, providing a measure of disruption |
| *Pursuit Tracking Task (PTT)* | PTTERR: the root mean-squared Euclidean error |
| **DECISION AND REACTION TIME** | |
| *Lexical Decision Time (LDT)* | LDTNPW: average latency for correct detection of non-primed word (ms; N=8)  LDTNWD: average latency for correct detection of non-word (ms)  LDTPWD: average latency for correct detection of primed word (ms; N=8)  Derived Measures:  LDT (NWD-PWD): Subtraction of NWD and PWD decision time,  reflecting lexical access time (ms)  LDT (NWD-NPW): Subtraction of NWD and NPW decision time,  reflecting lexical access time (ms)  LDT (PWD-NPW): Subtraction of NPW and PWD decision time,  reflecting semantic priming (ms) |
| *Simple Reaction Time (SRT)* | SRTSRT: mean stimulus recognition time  SRTMRT: mean motor transport time  SRTTT: mean total time (ms) |
| **E-DIARIES: Self-reported sleep parameters** | |
| *Visual Analogue Scale (VAS)* | sQoS: quality of sleep (0-100 VAS, 100 is worse)  sRuA: refreshed upon awakening (0-100 VAS, 100 is best)  sSleep-Lat: sleep onset latency (sec)  sNAW: number of awakenings |

**Note.** sQoS scale has been reversed for figures and tables.
