# Supplementary material for: Rapid Eye Movement Sleep, Sleep Continuity and Slow Wave Sleep as Predictors of Cognition, Mood, and Subjective Sleep Quality in Healthy Men and Women, Aged 20–84 Years
Source: Front Psychiatry. 2018 Jun 22;9:255. doi: 10.3389/fpsyt.2018.00255 (PMC6024010; doi:10.3389/fpsyt.2018.00255)
Supplement: Supplemental Table 7 — Cognitive performance variables by age and sex in the total sample. [file Table_7.DOCX]

**Supplemental Table 7.** Cognitive performance variables by age and sex in the total sample.

|  | **Age** | |  | **Sex** | |  | **Age x Sex** | |  | **Post-hoc contrasts** | | |
| --- | --- | --- | --- | --- | --- | --- | --- | --- | --- | --- | --- | --- |
| **Cognitive Variable** | F (df = 5) | P-value |  | F (df = 1) | P-value |  | F (df = 5) | P-value |  | **Age** | **Sex** | **Age x Sex** |
| **Affect and Mood** |  |  |  |  |  |  |  |  |  |  |  |  |
| PANASPOS | 13.72 | **<.0001*** |  | 1.57 | 0.211 |  | 0.92 | 0.467 |  | A1 < A3, A4, A5, A6; A2 < A3, A4, A5, A6; A3 < A5 |  |  |
| PANASNEG | 0.09 | 0.993 |  | 0 | 0.977 |  | 0.86 | 0.512 |  |  |  |  |
| LARSSED | 1.25 | 0.289 |  | 1.4 | 0.239 |  | 0.32 | 0.903 |  |  |  |  |
| LARSANXI | 2.3 | **0.046** |  | 0.04 | 0.836 |  | 0.93 | 0.46 |  | A6 < A1, A2, A3 |  |  |
| LARSCLUM | 1.68 | 0.141 |  | 0.06 | 0.803 |  | 1.25 | 0.286 |  |  |  |  |
| LARSDEPR | 3.05 | **0.011** |  | 0.14 | 0.711 |  | 0.84 | 0.521 |  | A6 < A1, A2, A3, A5 |  |  |
| LARSDIZZ | 4.63 | **0.0005*** |  | 0.15 | 0.702 |  | 0.79 | 0.56 |  | A6 < A1, A2, A3, A4, A5 |  |  |
| LARSDROW | 3.35 | **0.006** |  | 0.08 | 0.773 |  | 0.65 | 0.659 |  | A1 > A5, A6; A3 > A6 |  |  |
| LARSENER | 3 | **0.012** |  | 2.61 | 0.108 |  | 1.04 | 0.393 |  | A1 < A4, A5, A6 |  |  |
| LARSHAPP | 1.85 | 0.105 |  | 2.6 | 0.108 |  | 2.13 | 0.064 |  |  |  |  |
| LARSRELA | 2.3 | **0.046** |  | 2.16 | 0.143 |  | 1.12 | 0.352 |  | A4 > A1, A2, A3, A5, A6 |  |  |
| LARSSAD | 3 | **0.013** |  | 0.43 | 0.514 |  | 1.1 | 0.363 |  | A6 < A1, A2, A3, A5 |  |  |
| LARSTIRE | 3.93 | **0.002** |  | 1.14 | 0.287 |  | 0.58 | 0.714 |  | A1 > A4, A5, A6; A3 > A5, A6 |  |  |
| **Working Memory** |  |  |  |  |  |  |  |  |  |  |  |  |
| S1BKPCT | 0.81 | 0.54 |  | 2.18 | 0.141 |  | 1.75 | 0.126 |  |  |  |  |
| S2BKPCT | 6.43 | **<.0001*** |  | 1.41 | 0.236 |  | 1.08 | 0.373 |  | A1 > A3, A5, A6; A2 > A5, A6; A3 > A6; A4 > A6 |  |  |
| S1-2BKPCT | 9.23 | **<.0001*** |  | 0.08 | 0.784 |  | 1.57 | 0.171 |  | A1 < A3, A4, A5, A6; A2 < A5, A6; A3 < A6; A4 < A6 |  |  |
| V1BKPCT | 0.79 | 0.561 |  | 0.71 | 0.4 |  | 0.73 | 0.605 |  |  |  |  |
| V2BKPCT | 6.17 | **<.0001*** |  | 0.22 | 0.643 |  | 1.03 | 0.401 |  | A1 > A3, A4, A5, A6; A2 > A5, A6 |  |  |
| V1-2BKPCT | 8.27 | **<.0001*** |  | 0 | 0.958 |  | 1.34 | 0.251 |  | A1 < A3, A4, A5, A6; A2 < A4, A5, A6; A3 < A5 |  |  |
| **Arousal and Sustained Attention** |  |  |  |  |  |  |  |  |  |  |  |  |
| CFFDN | 2.74 | **0.021** |  | 1.92 | 0.168 |  | 0.72 | 0.608 |  | A1 > A5; A1 > A6; A3 > A5 |  |  |
| CFFUP | 10.18 | **<.0001*** |  | 5.64 | **0.019** |  | 0.8 | 0.547 |  | A1 > A4, A5, A6; A2 > A5, A6; A3 > A4, A5, A6; A4 > A5, A6 | M > F |  |
| CFFIU | 1.27 | 0.277 |  | 0.26 | 0.609 |  | 0.56 | 0.729 |  |  |  |  |
| CFFMED | 6.94 | **<.0001*** |  | 4.4 | **0.037** |  | 0.73 | 0.599 |  | A1 > A5, A6; A2 > A5, A6; A3 > A5, A6; A4 > A5 | M > F |  |
| CFFPSE | 6.43 | **<.0001*** |  | 4 | **0.047** |  | 0.82 | 0.534 |  | A1 > A5, A6; A2 > A5, A6; A3 > A5, A6 | M > F |  |
| SARTEOC | 3.32 | **0.007** |  | 1.59 | 0.208 |  | 0.71 | 0.617 |  | A1 > A3, A4, A5; A6 > A3, A4, A5 |  |  |
| SARTEOO | 2.03 | 0.076 |  | 0.09 | 0.763 |  | 1.21 | 0.307 |  |  |  |  |
| SARTACC | 2.43 | **0.037** |  | 1.99 | 0.16 |  | 0.98 | 0.431 |  | A6 < A1, A2, A3, A4, A5 |  |  |
| DSSTNUM | 59.36 | **<.0001*** |  | 0.03 | 0.861 |  | 0.99 | 0.428 |  | A1 > A2, A3, A4, A5, A6; A2 > A4, A5, A6; A3 > A4, A5, A6; A4 > A5, A6 |  |  |
| DSSTCOR | 58.6 | **<.0001*** |  | 0.24 | 0.626 |  | 1.53 | 0.182 |  | A1 > A2, A3, A4, A5, A6; A2 > A4, A5, A6; A3 > A4, A5, A6; A4 > A5, A6 |  |  |
| **Executive Function** |  |  |  |  |  |  |  |  |  |  |  |  |
| GNTNCOR | 0.94 | 0.457 |  | 3.59 | 0.06 |  | 1.26 | 0.282 |  |  |  |  |
| GNTCORB | 0.91 | 0.477 |  | 1.15 | 0.285 |  | 1.24 | 0.292 |  |  |  |  |
| GNTCORA | 1.78 | 0.121 |  | 5.27 | **0.023** |  | 0.91 | 0.477 |  |  | M > F |  |
| PVSAT | 4.71 | **0.0004*** |  | 3.32 | 0.07 |  | 4.3 | **0.001*** |  | A1 > A5, A6; A2 > A6; A3 > A5, A6; A4 > A6 |  | A1M > A4M; A1F > A5F; A1F > A6F; A2M > A2F; A2M > A4M; A2F < A3F; A2F > A6F; A3M < A3F; A3F > A5F; A3F > A6F; A4F > A5F; A4F > A6F; A5M > A5F; A6M > A6F |
| VFTUCI | 1.16 | 0.33 |  | 2.75 | 0.099 |  | 0.25 | 0.938 |  |  |  |  |
| VFTECI | 1 | 0.419 |  | 0.02 | 0.902 |  | 0.16 | 0.978 |  |  |  |  |
| **Sequence and Motor Control** |  |  |  |  |  |  |  |  |  |  |  |  |
| SERRTSEQB | 36.9 | **<.0001*** |  | 5.59 | **0.019** |  | 3.72 | **0.003** |  | A1 < A2, A3, A4, A5, A6; A2 < A3, A4, A5, A6; A3 < A5, A6; A4 < A5, A6 | M < F | A1M < A3M, A4M, A5M, A6M; A1F < A2F, A3F, A4F, A5F, A6F; A2M < A2F, A3M, A4M, A5M, A6M; A2F < A6F; A3M < A5M, A6M; A3F < A5F, A6F; A4F < A6F |
| SERRTSEQA | 39.07 | **<.0001*** |  | 7.38 | **0.007** |  | 4.45 | **0.0008*** |  | A1 < A2, A3, A4, A5, A6; A2 < A3, A4, A5, A6; A3 < A5, A6; A4 < A5, A6 | M < F | A1M < A3M, A4M, A5M, A6M; A1F < A2F, A3F, A4F, A5F, A6F; A2M < A2F, A3M, A4M, A5M, A6M; A3M < A5M, A6M; A3F < A4F, A5F, A6F; A4M < A6M; A4F < A6F |
| SERRTRAN | 32.98 | **<.0001*** |  | 6.95 | **0.009** |  | 1.41 | 0.224 |  | A1 < A2, A3, A4, A5, A6; A2 < A4, A5, A6; A3 < A5, A6 | M < F |  |
| SERRT (RAN-SEQB) | 2.96 | **0.014** |  | 0.02 | 0.901 |  | 3.39 | **0.006** |  | A1 > A6; A2 > A4, A5, A6; A3 > A6 |  | A1M > A4M, A5M, A6M; A2M > A5M, A6M; A3M > A6M |
| SERRT (RAN-SEQA) | 2.78 | **0.02** |  | 0.72 | 0.399 |  | 3.05 | **0.012** |  | A1 > A6; A2 > A4, A5, A6; A3 > A6 |  | A1M > A1F; A1M > A5M, A6M; A2M > A2F; A2M > A4M, A5M, A6M; A3M > A5M, A6M; A5M < A5F |
| SERRT (SEQA-SEQB) | 0.25 | 0.939 |  | 0 | 0.97 |  | 0.44 | 0.819 |  |  |  |  |
| PTTERR | 21.8 | **<.0001*** |  | 16.94 | **<.0001*** |  | 1.09 | 0.367 |  | A1 < A3, A4, A5, A6; A2 < A5, A6; A3 < A5, A6; A4 < A5, A6 | M < F |  |
| **Decision and Reaction Time** |  |  |  |  |  |  |  |  |  |  |  |  |
| LDTNPW | 4.36 | **0.0009*** |  | 0.01 | 0.935 |  | 0.27 | 0.93 |  | A1 < A4, A5, A6; A3 < A4, A6 |  |  |
| LDTNWD | 5.32 | **0.0001*** |  | 0.64 | 0.423 |  | 0.84 | 0.525 |  | A1 < A4, A5, A6; A2 < A5, A6; A3 < A5, A6 |  |  |
| LDTPWD | 5.91 | **<.0001*** |  | 0.2 | 0.654 |  | 0.59 | 0.709 |  | A1 < A4, A5, A6; A2 < A4, A5, A6; A3 < A4, A5, A6 |  |  |
| LDT (NWD-PWD) | 0.6 | 0.698 |  | 0.18 | 0.675 |  | 1.17 | 0.323 |  |  |  |  |
| LDT (NWD-NPW) | 0.63 | 0.679 |  | 0.67 | 0.415 |  | 1.37 | 0.238 |  |  |  |  |
| LDT (PWD-NPW) | 0.61 | 0.694 |  | 0.78 | 0.378 |  | 1.03 | 0.4 |  |  |  |  |
| SRTSRT | 11.42 | **<.0001*** |  | 3.5 | 0.063 |  | 1.59 | 0.166 |  | A1 < A3, A4, A5, A6; A2 < A4, A5, A6; A3 < A5, A6 |  |  |
| SRTMRT | 50.23 | **<.0001*** |  | 7.28 | **0.008** |  | 1.88 | 0.099 |  | A1 < A2, A3, A4, A5, A6; A2 < A3, A4, A5, A6; A3 < A5, A6; A4 < A5, A6; A5 < A6 | M < F |  |
| SRTTT | 40.4 | **<.0001*** |  | 6.97 | **0.009** |  | 1.88 | 0.1 |  | A1 < A3, A4, A5, A6; A2 < A3, A4, A5, A6; A3 < A5, A6; A4 < A5, A6; A5 < A6 | M < F |  |

**Note.** Performance variables are described in Supplemental Table 6. Bold values indicate significant correlations p < 0.05; * indicates significance levels of 0.05 that remain following FDR (False-Discovery Rate procedure as proposed by Benjamini–Hochberg–Yekutieli) correction. Post-hoc contrasts < 0.05 are reported and the direction of the contrast is indicated. M, male; F, female; Age groups: A1 18 - 29 years; A2 30 – 39 years; A3 40 – 49 years; A4 50 – 59 years; A5 60 – 69 years; A6 > 69 years. The number of observations included in analyses were as follows: n = 169 for GNTCORA, GNTCORB, GNTNCOR, VFTUCI, VFTECI; n = 179 for SERRT (RAN-SEQA); n = 180 for SERRT (RAN-SEQB); n = 182 for SERRT (SEQA-SEQB); n = 184 for SERRTSEQA; n = 185 for SERRTSEQB; n = 191 for SERRTRAN; n = 203 for SRTTT, SRTSRT, and SRTMRT, n = 206 for all remaining variables.
