# Supplementary material for: Rapid Eye Movement Sleep, Sleep Continuity and Slow Wave Sleep as Predictors of Cognition, Mood, and Subjective Sleep Quality in Healthy Men and Women, Aged 20–84 Years
Source: Front Psychiatry. 2018 Jun 22;9:255. doi: 10.3389/fpsyt.2018.00255 (PMC6024010; doi:10.3389/fpsyt.2018.00255)
Supplement: Supplemental Table 8 — Correlation (Kendall's tau) between PSG variables and cognition variables controlling for sex. [file Table_8.DOCX]

**Supplemental Table 8** .Correlation (Kendall’s tau) between PSG variables and cognition variables controlling for sex.

|  | **PSG variable, Kendall's Tau-values** | | | | | | | | | | | | |
| --- | --- | --- | --- | --- | --- | --- | --- | --- | --- | --- | --- | --- | --- |
| **Cognitive variable** | LPS | TST | SE | NAW | REM | Stage 1 | Stage 2 | Stage 4 | SWS | SWA | SWA% | SFA | SFA% |
| **Affect and Mood** |  |  |  |  |  |  |  |  |  |  |  |  |  |
| PANASPOS | -0.034 | -0.149 | **-0.208** | 0.062 | -0.053 | 0.047 | 0.076 | **-0.202** | **-0.191** | **-0.233** | **-0.259** | -0.052 | 0.119 |
| PANASNEG | 0.009 | -0.026 | 0.003 | 0.137 | -0.036 | 0.019 | -0.008 | -0.008 | 0.017 | -0.028 | 0.003 | -0.001 | -0.011 |
| LARSSED | 0.029 | 0.026 | 0.035 | 0.031 | 0.007 | 0.023 | -0.017 | 0.024 | 0.030 | 0.054 | 0.115 | 0.025 | -0.028 |
| LARSANXI | 0.089 | 0.035 | 0.033 | -0.065 | 0.061 | -0.041 | -0.085 | 0.109 | 0.077 | 0.103 | 0.154 | 0.049 | -0.056 |
| LARSCLUM | 0.058 | 0.023 | 0.029 | -0.019 | 0.044 | -0.016 | -0.078 | 0.095 | 0.081 | 0.066 | 0.132 | -0.015 | -0.078 |
| LARSDEPR | 0.107 | 0.053 | 0.040 | -0.039 | 0.081 | -0.047 | -0.091 | 0.118 | 0.091 | 0.113 | **0.199** | 0.064 | -0.046 |
| LARSDIZZ | 0.089 | 0.067 | 0.058 | -0.055 | 0.047 | -0.024 | -0.051 | 0.123 | 0.094 | 0.104 | **0.190** | 0.059 | -0.036 |
| LARSDROW | 0.059 | 0.042 | 0.068 | 0.000 | 0.054 | -0.024 | -0.070 | 0.083 | 0.068 | 0.147 | 0.158 | 0.065 | -0.067 |
| LARSENER | -0.056 | -0.026 | -0.075 | -0.018 | -0.030 | 0.021 | 0.046 | -0.044 | -0.040 | -0.111 | -0.075 | -0.044 | 0.035 |
| LARSHAPP | -0.097 | -0.013 | 0.013 | 0.001 | -0.033 | 0.018 | 0.015 | -0.053 | -0.022 | -0.031 | -0.108 | 0.036 | 0.052 |
| LARSRELA | -0.045 | -0.018 | 0.018 | 0.024 | -0.040 | 0.086 | 0.046 | -0.087 | -0.066 | 0.005 | 0.027 | 0.018 | 0.044 |
| LARSSAD | 0.071 | 0.035 | 0.025 | -0.046 | 0.066 | -0.043 | -0.089 | 0.102 | 0.068 | 0.085 | **0.182** | 0.028 | -0.045 |
| LARSTIRE | 0.054 | 0.065 | 0.092 | 0.031 | 0.022 | -0.032 | -0.050 | 0.086 | 0.101 | 0.131 | 0.126 | 0.070 | -0.060 |
| **Working Memory** |  |  |  |  |  |  |  |  |  |  |  |  |  |
| S1BKPCT | -0.018 | 0.042 | 0.073 | -0.125 | 0.015 | -0.035 | -0.031 | 0.114 | 0.050 | 0.133 | 0.087 | 0.104 | 0.019 |
| S2BKPCT | -0.064 | **0.165** | **0.209** | -0.128 | 0.028 | -0.057 | 0.007 | **0.179** | 0.141 | **0.239** | **0.228** | **0.163** | -0.009 |
| S1-2BKPCT | 0.060 | **-0.183** | **-0.233** | 0.088 | -0.024 | 0.054 | -0.001 | **-0.194** | **-0.172** | **-0.257** | **-0.245** | -0.162 | 0.029 |
| V1BKPCT | -0.085 | 0.066 | 0.099 | -0.068 | 0.060 | -0.063 | 0.012 | 0.067 | 0.020 | 0.065 | 0.027 | 0.105 | 0.083 |
| V2BKPCT | -0.083 | **0.210** | **0.246** | -0.095 | 0.051 | -0.039 | -0.002 | **0.199** | **0.181** | **0.267** | **0.206** | **0.197** | -0.004 |
| V1-2BKPCT | 0.069 | **-0.214** | **-0.246** | 0.098 | -0.049 | 0.037 | 0.010 | **-0.219** | **-0.209** | **-0.295** | **-0.227** | **-0.191** | 0.031 |
| **Arousal and Sustained Attention** |  |  |  |  |  |  |  |  |  |  |  |  |  |
| CFFDN | 0.027 | -0.039 | -0.031 | -0.020 | -0.085 | -0.054 | -0.023 | 0.075 | 0.049 | 0.042 | 0.040 | 0.056 | 0.031 |
| CFFUP | 0.011 | 0.059 | 0.072 | -0.084 | 0.030 | -0.049 | -0.062 | 0.140 | 0.131 | 0.131 | 0.127 | 0.123 | 0.021 |
| CFFIU | -0.049 | -0.064 | -0.068 | 0.045 | -0.067 | 0.008 | 0.039 | -0.040 | -0.069 | -0.155 | -0.090 | -0.082 | 0.045 |
| CFFMED | 0.014 | 0.014 | 0.033 | -0.045 | -0.028 | -0.045 | -0.044 | 0.117 | 0.096 | 0.100 | 0.090 | 0.095 | 0.025 |
| CFFPSE | 0.023 | 0.019 | 0.031 | -0.053 | -0.025 | -0.052 | -0.042 | 0.118 | 0.103 | 0.091 | 0.082 | 0.095 | 0.029 |
| SARTEOC | -0.053 | 0.074 | 0.115 | 0.044 | -0.050 | -0.030 | -0.002 | 0.060 | 0.111 | 0.093 | 0.059 | -0.011 | -0.084 |
| SARTEOO | 0.067 | -0.121 | -0.116 | 0.147 | -0.133 | 0.018 | -0.026 | -0.105 | -0.016 | -0.079 | -0.106 | -0.093 | -0.050 |
| SARTACC | 0.019 | 0.021 | -0.006 | -0.131 | 0.114 | 0.011 | -0.010 | 0.039 | -0.042 | 0.004 | 0.044 | 0.069 | 0.066 |
| DSSTNUM | -0.014 | **0.267** | **0.342** | **-0.160** | 0.085 | -0.063 | 0.005 | **0.290** | **0.226** | **0.325** | **0.341** | **0.249** | 0.000 |
| DSSTCOR | -0.016 | **0.263** | **0.336** | **-0.162** | 0.088 | -0.056 | -0.004 | **0.289** | **0.224** | **0.326** | **0.343** | **0.240** | -0.008 |
| **Executive Function** |  |  |  |  |  |  |  |  |  |  |  |  |  |
| GNTNCOR | -0.011 | 0.120 | 0.056 | **-0.347** | **0.210** | -0.126 | -0.021 | 0.166 | 0.066 | 0.113 | 0.159 | 0.021 | -0.094 |
| GNTCORB | 0.018 | 0.073 | 0.054 | -0.143 | 0.069 | -0.044 | 0.032 | 0.146 | 0.042 | 0.097 | 0.122 | 0.071 | -0.062 |
| GNTCORA | -0.012 | 0.129 | 0.064 | **-0.392** | **0.236** | -0.167 | -0.050 | **0.175** | 0.088 | 0.135 | 0.173 | 0.012 | -0.102 |
| PVSAT | -0.026 | 0.146 | **0.161** | -0.136 | 0.052 | -0.062 | 0.006 | 0.123 | 0.087 | **0.179** | 0.140 | **0.197** | 0.067 |
| VFTUCI | -0.018 | 0.061 | 0.046 | -0.103 | 0.015 | -0.142 | -0.041 | 0.135 | 0.133 | **0.206** | **0.184** | 0.026 | -0.148 |
| VFTECI | 0.041 | 0.044 | 0.070 | -0.016 | -0.018 | -0.047 | 0.040 | -0.006 | 0.008 | 0.032 | 0.058 | 0.128 | 0.121 |
| **Sequence and Motor Control** |  |  |  |  |  |  |  |  |  |  |  |  |  |
| SERRTSEQB | 0.008 | **-0.236** | **-0.314** | 0.097 | -0.025 | 0.070 | 0.025 | **-0.290** | **-0.247** | **-0.353** | **-0.306** | **-0.229** | 0.049 |
| SERRTSEQA | 0.027 | **-0.268** | **-0.340** | 0.113 | -0.040 | 0.055 | -0.009 | **-0.266** | **-0.233** | **-0.318** | **-0.281** | **-0.208** | 0.043 |
| SERRTRAN | 0.016 | **-0.213** | **-0.286** | 0.092 | -0.004 | 0.082 | 0.015 | **-0.263** | **-0.241** | **-0.310** | **-0.246** | **-0.199** | 0.067 |
| SERRT (RAN-SEQB) | -0.054 | 0.044 | 0.077 | -0.054 | 0.016 | -0.070 | -0.032 | 0.123 | 0.098 | 0.117 | 0.092 | 0.037 | -0.042 |
| SERRT (RAN-SEQA) | -0.080 | 0.101 | 0.103 | -0.082 | 0.056 | -0.029 | 0.053 | 0.045 | 0.037 | 0.010 | 0.056 | -0.004 | -0.005 |
| SERRT (SEQA-SEQB) | 0.006 | -0.045 | -0.007 | 0.008 | -0.005 | -0.037 | -0.072 | 0.083 | 0.036 | 0.110 | 0.036 | 0.061 | -0.030 |
| PTTERR | 0.055 | **-0.216** | **-0.259** | 0.151 | -0.112 | 0.080 | -0.013 | **-0.196** | **-0.171** | **-0.251** | **-0.241** | **-0.213** | -0.025 |
| **Decision and Reaction Time** |  |  |  |  |  |  |  |  |  |  |  |  |  |
| LDTNPW | -0.006 | -0.036 | -0.057 | 0.041 | 0.017 | 0.008 | 0.043 | -0.108 | -0.071 | -0.111 | -0.143 | -0.035 | 0.048 |
| LDTNWD | -0.064 | -0.082 | -0.098 | 0.059 | -0.042 | -0.018 | 0.064 | -0.112 | -0.082 | -0.135 | -0.138 | -0.025 | 0.066 |
| LDTPWD | -0.013 | -0.021 | -0.045 | 0.002 | 0.052 | -0.001 | 0.066 | -0.151 | -0.111 | -0.124 | -0.114 | -0.045 | 0.057 |
| LDT (NWD-PWD) | -0.068 | -0.049 | -0.052 | 0.038 | -0.070 | -0.019 | 0.018 | 0.029 | 0.025 | -0.013 | -0.012 | 0.014 | -0.008 |
| LDT (NWD-NPW) | -0.085 | -0.015 | -0.026 | -0.015 | -0.018 | -0.034 | 0.052 | -0.030 | -0.029 | -0.032 | 0.010 | -0.028 | -0.016 |
| LDT (PWD-NPW) | -0.045 | 0.022 | -0.002 | -0.067 | 0.087 | 0.008 | 0.021 | -0.092 | -0.088 | -0.043 | 0.030 | -0.037 | 0.019 |
| SRTSRT | 0.031 | **-0.199** | **-0.244** | **0.231** | -0.134 | 0.069 | 0.059 | **-0.199** | -0.153 | **-0.266** | **-0.266** | **-0.202** | 0.008 |
| SRTMRT | 0.009 | **-0.244** | **-0.309** | **0.173** | -0.109 | 0.065 | 0.020 | **-0.247** | **-0.197** | **-0.310** | **-0.334** | **-0.245** | -0.002 |
| SRTTT | 0.007 | **-0.239** | **-0.301** | **0.192** | -0.115 | 0.074 | 0.034 | **-0.250** | -0.200 | **-0.313** | **-0.333** | **-0.243** | 0.004 |

**Note**. Bold values indicate significance levels of 0.05 that remain following FDR (False-Discovery Rate procedure as proposed by Benjamini–Hochberg–Yekutieli) correction. PSG variables: LPS, latency to persistent sleep (min); TST, total sleep time (min); SE, sleep efficiency (%); NAW, number of awakenings; REM, rapid eye movement; Stage 1, duration of stage 1 sleep (min); Stage 2, duration of stage 2 sleep (min); Stage 4, duration of stage 4 sleep (min); SWS, slow wave sleep; SWA, slow wave activity (µV^2^); SWA%, slow wave activity in percentage of total power; SFA, sigma activity (µV^2^); SFA%, sigma activity in percentage of total power. Cognition variables are described in full in Supplemental Table 6. Number of observations is as follows: 1) SWA, SWA%, SFA, SFA%: n = 145 for GNTCORA, GNTCORB, GNTNCOR, VFTUCI, VFTECI, n = 155 for SERRT (RAN-SEQB), SERRT (RAN-SEQA), n = 158 for SERRT (SEQA-SEQB), n = 160 for SERRTSEQB, SERRTSEQA, n = 165 for SERRTRAN, n = 177 for SRTMRT, SRTSRT, SRTTT, n = 179 for all remaining variables; 2) LPS, TST, SE, NAW, REM, Stage 1, Stage 2, Stage 4, SWS: n = 163 for GNTCORA, GNTCORB, GNTNCOR, VFTUCI, VFTECI, n = 173 for SERRT (RAN-SEQA), n = 174 for SERRT (RAN-SEQB), n = 176 for SERRT (SEQA-SEQB), n = 178 for SERRTSEQA, n = 179 for SERRTSEQB, n = 185 for SERRTRAN, n = 197 for SRTMRT, SRTSRT, SRTTT, n = 200 for all remaining variables.
