# Supplementary material for: Rapid Eye Movement Sleep, Sleep Continuity and Slow Wave Sleep as Predictors of Cognition, Mood, and Subjective Sleep Quality in Healthy Men and Women, Aged 20–84 Years
Source: Front Psychiatry. 2018 Jun 22;9:255. doi: 10.3389/fpsyt.2018.00255 (PMC6024010; doi:10.3389/fpsyt.2018.00255)
Supplement: Supplemental Table 9 — Correlation (Kendall's tau) between PSG variables and cognition variables controlling for age and sex. [file Table_9.DOCX]

**Supplemental Table 9.** Correlation (Kendall’s tau) between PSG variables and cognition variables controlling for age and sex.

|  | **PSG variable, Kendall's Tau-values** | | | | | | | | | | | | |
| --- | --- | --- | --- | --- | --- | --- | --- | --- | --- | --- | --- | --- | --- |
| **Cognitive variable** | LPS | TST | SE | NAW | REM | Stage 1 | Stage 2 | Stage 4 | SWS | SWA | SWA% | SFA | SFA% |
| **Affect and Mood** |  |  |  |  |  |  |  |  |  |  |  |  |  |
| PANASPOS | -0.046 | -0.049 | -0.086 | 0.014 | -0.020 | 0.024 | 0.071 | -0.094 | -0.102 | -0.118 | -0.147 | 0.027 | 0.099 |
| PANASNEG | 0.011 | -0.050 | -0.027 | 0.149 | -0.043 | 0.024 | -0.007 | -0.035 | -0.003 | -0.046 | -0.013 | -0.010 | -0.008 |
| LARSSED | 0.032 | -0.009 | -0.011 | 0.048 | -0.004 | 0.032 | -0.015 | -0.016 | -0.003 | 0.003 | 0.070 | -0.006 | -0.018 |
| LARSANXI | 0.095 | -0.015 | -0.032 | -0.043 | 0.046 | -0.030 | -0.082 | 0.059 | 0.033 | 0.052 | 0.107 | 0.016 | -0.046 |
| LARSCLUM | 0.063 | -0.020 | -0.026 | 0.000 | 0.031 | -0.006 | -0.076 | 0.052 | 0.044 | 0.025 | 0.098 | -0.042 | -0.070 |
| LARSDEPR | 0.113 | 0.002 | -0.028 | -0.016 | 0.066 | -0.036 | -0.088 | 0.065 | 0.045 | 0.059 | 0.155 | 0.030 | -0.035 |
| LARSDIZZ | 0.097 | 0.001 | -0.029 | -0.025 | 0.026 | -0.008 | -0.047 | 0.053 | 0.034 | 0.029 | 0.125 | 0.014 | -0.021 |
| LARSDROW | 0.066 | -0.028 | -0.020 | 0.033 | 0.033 | -0.008 | -0.067 | 0.007 | 0.004 | 0.066 | 0.077 | 0.014 | -0.051 |
| LARSENER | -0.061 | 0.024 | -0.015 | -0.041 | -0.015 | 0.010 | 0.043 | 0.011 | 0.005 | -0.062 | -0.022 | -0.012 | 0.025 |
| LARSHAPP | -0.099 | 0.001 | 0.033 | -0.005 | -0.029 | 0.015 | 0.014 | -0.041 | -0.010 | -0.021 | -0.106 | 0.045 | 0.050 |
| LARSRELA | -0.045 | -0.015 | 0.026 | 0.022 | -0.039 | 0.085 | 0.046 | -0.088 | -0.064 | 0.012 | 0.035 | 0.022 | 0.043 |
| LARSSAD | 0.076 | -0.010 | -0.035 | -0.026 | 0.053 | -0.033 | -0.086 | 0.056 | 0.028 | 0.043 | 0.149 | 0.000 | -0.037 |
| LARSTIRE | 0.061 | -0.003 | 0.007 | 0.064 | 0.001 | -0.016 | -0.046 | 0.011 | 0.040 | 0.055 | 0.049 | 0.023 | -0.044 |
| **Working Memory** |  |  |  |  |  |  |  |  |  |  |  |  |  |
| S1BKPCT | -0.014 | 0.001 | 0.024 | -0.108 | 0.002 | -0.026 | -0.028 | 0.075 | 0.013 | 0.092 | 0.041 | 0.078 | 0.029 |
| S2BKPCT | -0.058 | 0.069 | 0.092 | -0.086 | -0.004 | -0.035 | 0.016 | 0.073 | 0.050 | 0.131 | 0.117 | 0.097 | 0.017 |
| S1-2BKPCT | 0.054 | -0.078 | -0.105 | 0.039 | 0.013 | 0.029 | -0.011 | -0.076 | -0.073 | -0.137 | -0.121 | -0.087 | 0.002 |
| V1BKPCT | -0.083 | 0.039 | 0.067 | -0.055 | 0.051 | -0.057 | 0.014 | 0.036 | -0.009 | 0.040 | -0.002 | 0.091 | 0.089 |
| V2BKPCT | -0.079 | 0.111 | 0.123 | -0.047 | 0.017 | -0.014 | 0.007 | 0.085 | 0.086 | 0.150 | 0.077 | 0.126 | 0.025 |
| V1-2BKPCT | 0.064 | -0.113 | -0.119 | 0.050 | -0.015 | 0.012 | 0.001 | -0.104 | -0.114 | -0.179 | -0.098 | -0.118 | 0.004 |
| **Arousal and Sustained Attention** |  |  |  |  |  |  |  |  |  |  |  |  |  |
| CFFDN | 0.030 | -0.079 | -0.083 | -0.004 | -0.097 | -0.046 | -0.021 | 0.038 | 0.017 | -0.007 | -0.010 | 0.028 | 0.041 |
| CFFUP | 0.019 | -0.022 | -0.033 | -0.049 | 0.005 | -0.032 | -0.057 | 0.056 | 0.061 | 0.021 | 0.015 | 0.061 | 0.046 |
| CFFIU | -0.052 | -0.034 | -0.030 | 0.031 | -0.058 | 0.001 | 0.037 | -0.005 | -0.041 | -0.108 | -0.034 | -0.050 | 0.034 |
| CFFMED | 0.020 | -0.056 | -0.056 | -0.016 | -0.050 | -0.030 | -0.040 | 0.048 | 0.037 | 0.010 | -0.002 | 0.044 | 0.044 |
| CFFPSE | 0.029 | -0.046 | -0.053 | -0.025 | -0.046 | -0.039 | -0.038 | 0.054 | 0.048 | 0.006 | -0.005 | 0.046 | 0.047 |
| SARTEOC | -0.050 | 0.037 | 0.073 | 0.063 | -0.063 | -0.021 | 0.001 | 0.018 | 0.079 | 0.041 | 0.002 | -0.045 | -0.074 |
| SARTEOO | 0.063 | -0.062 | -0.040 | 0.121 | -0.116 | 0.003 | -0.031 | -0.037 | 0.047 | -0.011 | -0.040 | -0.055 | -0.064 |
| SARTACC | 0.020 | 0.008 | -0.025 | -0.127 | 0.110 | 0.014 | -0.009 | 0.026 | -0.058 | -0.002 | 0.042 | 0.068 | 0.068 |
| DSSTNUM | 0.003 | 0.090 | 0.125 | -0.088 | 0.028 | -0.021 | 0.026 | 0.090 | 0.052 | 0.113 | 0.130 | 0.135 | 0.060 |
| DSSTCOR | 0.001 | 0.091 | 0.125 | -0.092 | 0.034 | -0.014 | 0.013 | 0.096 | 0.055 | 0.122 | 0.141 | 0.128 | 0.048 |
| **Executive Function** |  |  |  |  |  |  |  |  |  |  |  |  |  |
| GNTNCOR | -0.001 | 0.075 | -0.001 | **-0.325** | 0.183 | -0.114 | -0.021 | 0.126 | 0.032 | 0.063 | 0.099 | -0.016 | -0.094 |
| GNTCORB | 0.031 | 0.009 | -0.023 | -0.102 | 0.025 | -0.027 | 0.033 | 0.090 | -0.005 | 0.035 | 0.042 | 0.029 | -0.061 |
| GNTCORA | -0.003 | 0.091 | 0.014 | **-0.376** | 0.213 | -0.157 | -0.050 | 0.142 | 0.059 | 0.090 | 0.120 | -0.023 | -0.102 |
| PVSAT | -0.020 | 0.078 | 0.077 | -0.106 | 0.030 | -0.046 | 0.012 | 0.045 | 0.019 | 0.095 | 0.049 | 0.149 | 0.088 |
| VFTUCI | -0.011 | 0.025 | 0.002 | -0.079 | -0.012 | -0.134 | -0.042 | 0.105 | 0.109 | 0.170 | 0.137 | -0.005 | -0.148 |
| VFTECI | 0.047 | 0.019 | 0.043 | 0.002 | -0.038 | -0.040 | 0.041 | -0.033 | -0.011 | -0.003 | 0.014 | 0.108 | 0.123 |
| **Sequence and Motor Control** |  |  |  |  |  |  |  |  |  |  |  |  |  |
| SERRTSEQB | 0.010 | -0.094 | -0.135 | 0.036 | 0.010 | 0.050 | 0.009 | -0.136 | -0.114 | -0.197 | -0.145 | -0.133 | 0.012 |
| SERRTSEQA | 0.023 | -0.130 | -0.164 | 0.055 | -0.007 | 0.036 | -0.029 | -0.108 | -0.102 | -0.160 | -0.125 | -0.107 | 0.013 |
| SERRTRAN | 0.004 | -0.066 | -0.107 | 0.026 | 0.046 | 0.059 | 0.001 | -0.110 | -0.115 | -0.153 | -0.077 | -0.099 | 0.036 |
| SERRT (RAN-SEQB) | -0.052 | 0.021 | 0.050 | -0.044 | 0.012 | -0.066 | -0.029 | 0.103 | 0.078 | 0.106 | 0.079 | 0.026 | -0.037 |
| SERRT (RAN-SEQA) | -0.079 | 0.091 | 0.093 | -0.077 | 0.054 | -0.026 | 0.054 | 0.030 | 0.024 | 0.004 | 0.055 | -0.008 | -0.003 |
| SERRT (SEQA-SEQB) | 0.007 | -0.054 | -0.015 | 0.010 | -0.006 | -0.037 | -0.071 | 0.082 | 0.032 | 0.101 | 0.021 | 0.052 | -0.026 |
| PTTERR | 0.048 | -0.094 | -0.106 | 0.099 | -0.077 | 0.053 | -0.025 | -0.052 | -0.051 | -0.103 | -0.089 | -0.130 | -0.063 |
| **Decision and Reaction Time** |  |  |  |  |  |  |  |  |  |  |  |  |  |
| LDTNPW | -0.013 | 0.039 | 0.038 | 0.008 | 0.041 | -0.009 | 0.038 | -0.031 | -0.005 | -0.031 | -0.066 | 0.015 | 0.032 |
| LDTNWD | -0.075 | 0.009 | 0.018 | 0.019 | -0.014 | -0.041 | 0.060 | -0.012 | 0.003 | -0.026 | -0.027 | 0.044 | 0.046 |
| LDTPWD | -0.020 | 0.054 | 0.051 | -0.032 | 0.077 | -0.018 | 0.062 | -0.078 | -0.048 | -0.049 | -0.036 | 0.003 | 0.042 |
| LDT (NWD-PWD) | -0.071 | -0.024 | -0.020 | 0.026 | -0.062 | -0.025 | 0.016 | 0.064 | 0.053 | 0.023 | 0.025 | 0.036 | -0.015 |
| LDT (NWD-NPW) | -0.088 | 0.020 | 0.017 | -0.031 | -0.008 | -0.042 | 0.050 | 0.008 | 0.002 | 0.003 | 0.050 | -0.007 | -0.023 |
| LDT (PWD-NPW) | -0.046 | 0.036 | 0.013 | -0.073 | 0.091 | 0.006 | 0.021 | -0.085 | -0.082 | -0.040 | 0.040 | -0.035 | 0.018 |
| SRTSRT | 0.024 | -0.093 | -0.111 | 0.193 | -0.105 | 0.043 | 0.051 | -0.076 | -0.045 | -0.129 | -0.130 | -0.121 | -0.025 |
| SRTMRT | -0.005 | -0.087 | -0.114 | 0.113 | -0.065 | 0.026 | 0.003 | -0.061 | -0.034 | -0.112 | -0.144 | -0.136 | -0.055 |
| SRTTT | -0.007 | -0.086 | -0.111 | 0.136 | -0.073 | 0.038 | 0.019 | -0.071 | -0.044 | -0.122 | -0.148 | -0.136 | -0.046 |

**Note.** Bold values indicate significance levels of 0.05 that remain following FDR (False-Discovery Rate procedure as proposed by Benjamini–Hochberg–Yekutieli) correction. PSG variables: LPS, latency to persistent sleep (min); TST, total sleep time (min); SE, sleep efficiency (%); NAW, number of awakenings; REM, rapid eye movement; Stage 1, duration of stage 1 sleep (min); Stage 2, duration of stage 2 sleep (min); Stage 4, duration of stage 4 sleep (min)SWS, slow wave sleep; SWA, slow wave activity (µV^2^); SWA%, slow wave activity in percentage of total power; SFA, sigma activity (µV^2^); SFA%, sigma activity in percentage of total power. Cognition variables are described in full in Supplemental Table 6. Number of observations is as follows: 1) SWA, SWA%, SFA, SFA%: n = 145 for GNTCORA, GNTCORB, GNTNCOR, VFTUCI, VFTECI, n = 155 for SERRT (RAN-SEQB), SERRT (RAN-SEQA), n = 158 for SERRT (SEQA-SEQB), n = 160 for SERRTSEQB, SERRTSEQA, n = 165 for SERRTRAN, n = 177 for SRTMRT, SRTSRT, SRTTT, n = 179 for all remaining variables; 2) LPS, TST, SE, NAW, REM, Stage 1, Stage 2, Stage 4, SWS: n = 163 for GNTCORA, GNTCORB, GNTNCOR, VFTUCI, VFTECI, n = 173 for SERRT (RAN-SEQA), n = 174 for SERRT (RAN-SEQB), n = 176 for SERRT (SEQA-SEQB), n = 178 for SERRTSEQA, n = 179 for SERRTSEQB, n = 185 for SERRTRAN, n = 197 for SRTMRT, SRTSRT, SRTTT, n = 200 for all remaining variables.
