# Supplementary material for: Rapid Eye Movement Sleep, Sleep Continuity and Slow Wave Sleep as Predictors of Cognition, Mood, and Subjective Sleep Quality in Healthy Men and Women, Aged 20–84 Years
Source: Front Psychiatry. 2018 Jun 22;9:255. doi: 10.3389/fpsyt.2018.00255 (PMC6024010; doi:10.3389/fpsyt.2018.00255)
Supplement: Supplemental Table 10 — Correlation between PSG variables and cognition factors controlling for sex. [file Table_10.DOCX]

**Supplemental Table 10.** Correlation between PSG variables and cognition factors controlling for sex.

|  | **PSG Sleep variable, Kendall's Tau-values** | | | | | | | | | | | | |
| --- | --- | --- | --- | --- | --- | --- | --- | --- | --- | --- | --- | --- | --- |
| **Cognition Factor** | LPS | TST | SE | NAW | REM | Stage 1 | Stage 2 | Stage 4 | SWS | SWA | SWA% | SFA | SFA% |
| negMood/Arousal | 0.057 | 0.037 | 0.044 | 0.010 | 0.025 | -0.041 | -0.062 | 0.084 | 0.082 | 0.107 | 0.121 | 0.019 | -0.086 |
| Response time | 0.015 | **-0.241** | **-0.318** | 0.127 | -0.072 | 0.059 | 0.026 | **-0.283** | **-0.238** | **-0.348** | **-0.332** | **-0.200** | 0.068 |
| Accuracy | -0.062 | 0.107 | 0.091 | **-0.166** | 0.123 | -0.003 | 0.032 | 0.056 | -0.008 | 0.065 | 0.119 | 0.080 | 0.040 |
| Visual-Perceptual Sensitivity | 0.014 | -0.056 | -0.052 | 0.014 | -0.072 | -0.028 | -0.046 | 0.043 | 0.047 | 0.008 | -0.002 | 0.044 | 0.043 |

**Note.** Bold values indicate significance levels of 0.05 that remain following FDR (False-Discovery Rate procedure as proposed by Benjamini–Hochberg–Yekutieli) correction. PSG variables: LPS, latency to persistent sleep (min); TST, total sleep time (min); SE, sleep efficiency (%); NAW, number of awakenings; REM, rapid eye movement; Stage 1, duration of stage 1 sleep (min); Stage 2, duration of stage 2 sleep (min); Stage 4, duration of stage 4 sleep (min); SWS, slow wave sleep; SWA, slow wave activity (µV^2^); SWA%, slow wave activity in percentage of total power; SFA, sigma activity (µV^2^); SFA%, sigma activity in percentage of total power. Number of observations for all four factors is as follows: n = 179 for SWA, SWA%, SFA and SFA%, n = 200 for all remaining variables.
