# Supplementary material for: Rapid Eye Movement Sleep, Sleep Continuity and Slow Wave Sleep as Predictors of Cognition, Mood, and Subjective Sleep Quality in Healthy Men and Women, Aged 20–84 Years
Source: Front Psychiatry. 2018 Jun 22;9:255. doi: 10.3389/fpsyt.2018.00255 (PMC6024010; doi:10.3389/fpsyt.2018.00255)
Supplement: Supplemental Table 11 — Correlation between PSG variables and cognition factors controlling for age and sex. [file Table_11.DOCX]

**Supplemental Table 11.** Correlation between PSG variables and cognition factors controlling for age and sex.

|  | **PSG Sleep variable, Kendall's Tau-values** | | | | | | | | | | | | |
| --- | --- | --- | --- | --- | --- | --- | --- | --- | --- | --- | --- | --- | --- |
| **Cognition Factor** | LPS | TST | SE | NAW | REM | Stage 1 | Stage 2 | Stage 4 | SWS | SWA | SWA% | SFA | SFA% |
| negMood/Arousal | 0.062 | -0.013 | -0.020 | 0.034 | 0.010 | -0.030 | -0.059 | 0.031 | 0.038 | 0.061 | 0.076 | -0.012 | -0.077 |
| Response time | -0.001 | -0.078 | -0.120 | 0.054 | -0.018 | 0.021 | 0.015 | -0.105 | -0.087 | -0.166 | -0.142 | -0.085 | 0.029 |
| Accuracy | -0.059 | 0.076 | 0.052 | -0.153 | 0.114 | 0.005 | 0.035 | 0.019 | -0.043 | 0.025 | 0.084 | 0.057 | 0.049 |
| Visual-Perceptual Sensitivity | 0.016 | -0.080 | -0.084 | 0.023 | -0.079 | -0.024 | -0.045 | 0.023 | 0.030 | -0.028 | -0.040 | 0.024 | 0.050 |

**Note.** PSG variables: LPS, latency to persistent sleep (min); TST, total sleep time (min); SE, sleep efficiency (%); NAW, number of awakenings; REM, rapid eye movement; Stage 1, duration of stage 1 sleep (min); Stage 2, duration of stage 2 sleep (min); Stage 4, duration of stage 4 sleep (min); SWS, slow wave sleep; SWA, slow wave activity (µV^2^); SWA%, slow wave activity in percentage of total power; SFA, sigma activity (µV^2^); SFA%, sigma activity in percentage of total power. Number of observations for all four factors is as follows: n = 179 for SWA, SWA%, SFA and SFA%, n = 200 for all remaining variables.
