# Supplementary material for: Rapid Eye Movement Sleep, Sleep Continuity and Slow Wave Sleep as Predictors of Cognition, Mood, and Subjective Sleep Quality in Healthy Men and Women, Aged 20–84 Years
Source: Front Psychiatry. 2018 Jun 22;9:255. doi: 10.3389/fpsyt.2018.00255 (PMC6024010; doi:10.3389/fpsyt.2018.00255)
Supplement: Supplemental Table 12 — Correlation between PSG variables and cognition factors by sex controlling for age. [file Table_12.DOCX]

**Supplemental Table 12.** Correlation between PSG variables and cognition factors by sex controlling for age.

|  |  | **Cognition factor, Kendall's Tau and p-values** | | | | | | | | | | |
| --- | --- | --- | --- | --- | --- | --- | --- | --- | --- | --- | --- | --- |
|  |  | negMood/Arousal | |  | Response time | |  | Accuracy | |  | Visual-Perceptual Sensitivity | |
| **PSG Sleep variable** | **Sex** | *τ* | *p-value* |  | *τ* | *p-value* |  | *τ* | *p-value* |  | *τ* | *p-value* |
| LPS | *M* | 0.131 | 0.071 |  | 0.096 | 0.186 |  | -0.031 | 0.666 |  | -0.108 | 0.140 |
|  | *F* | 0.019 | 0.763 |  | -0.053 | 0.410 |  | -0.072 | 0.261 |  | 0.091 | 0.155 |
| TST | *M* | -0.097 | 0.185 |  | -0.159 | 0.030 |  | 0.033 | 0.650 |  | -0.010 | 0.896 |
|  | *F* | 0.052 | 0.414 |  | -0.032 | 0.621 |  | 0.105 | 0.099 |  | -0.125 | 0.050 |
| SE | *M* | -0.062 | 0.398 |  | -0.181 | 0.013 |  | 0.062 | 0.395 |  | -0.018 | 0.800 |
|  | *F* | 0.011 | 0.858 |  | -0.090 | 0.157 |  | 0.037 | 0.560 |  | -0.129 | 0.042 |
| NAW | *M* | 0.156 | 0.033 |  | 0.126 | 0.084 |  | -0.188 | 0.010 |  | -0.041 | 0.571 |
|  | *F* | -0.061 | 0.335 |  | 0.025 | 0.693 |  | -0.117 | 0.067 |  | 0.058 | 0.360 |
| REM | *M* | -0.053 | 0.469 |  | -0.045 | 0.539 |  | 0.087 | 0.232 |  | -0.015 | 0.833 |
|  | *F* | 0.046 | 0.473 |  | 0.000 | 0.997 |  | 0.143 | 0.025 |  | -0.106 | 0.096 |
| Stage 1 | *M* | -0.026 | 0.723 |  | 0.110 | 0.131 |  | -0.061 | 0.403 |  | -0.064 | 0.383 |
|  | *F* | -0.037 | 0.566 |  | -0.059 | 0.354 |  | 0.031 | 0.629 |  | -0.012 | 0.855 |
| Stage 2 | *M* | -0.054 | 0.455 |  | -0.034 | 0.645 |  | -0.007 | 0.928 |  | 0.022 | 0.764 |
|  | *F* | -0.070 | 0.274 |  | 0.028 | 0.661 |  | 0.060 | 0.347 |  | -0.076 | 0.233 |
| Stage 4 | *M* | -0.020 | 0.784 |  | -0.164 | 0.024 |  | 0.087 | 0.233 |  | -0.016 | 0.821 |
|  | *F* | 0.073 | 0.251 |  | -0.029 | 0.653 |  | -0.009 | 0.891 |  | 0.059 | 0.356 |
| SWS | *M* | -0.029 | 0.690 |  | -0.144 | 0.048 |  | 0.033 | 0.651 |  | 0.024 | 0.747 |
|  | *F* | 0.094 | 0.141 |  | -0.011 | 0.867 |  | -0.061 | 0.340 |  | 0.038 | 0.547 |
| SWA | *M* | 0.079 | 0.302 |  | -0.157 | 0.040 |  | 0.053 | 0.486 |  | -0.021 | 0.784 |
|  | *F* | 0.040 | 0.553 |  | -0.163 | 0.016 |  | 0.030 | 0.653 |  | -0.030 | 0.660 |
| SWA% | *M* | 0.153 | 0.045 |  | -0.112 | 0.143 |  | 0.168 | 0.028 |  | -0.065 | 0.397 |
|  | *F* | 0.020 | 0.766 |  | -0.156 | 0.021 |  | 0.020 | 0.769 |  | -0.014 | 0.842 |
| SFA | *M* | 0.014 | 0.850 |  | -0.163 | 0.034 |  | 0.052 | 0.502 |  | 0.038 | 0.622 |
|  | *F* | -0.041 | 0.550 |  | -0.029 | 0.667 |  | 0.058 | 0.392 |  | 0.019 | 0.781 |
| SFA% | *M* | -0.108 | 0.160 |  | -0.054 | 0.482 |  | 0.040 | 0.603 |  | 0.040 | 0.599 |
|  | *F* | -0.066 | 0.332 |  | 0.061 | 0.366 |  | 0.036 | 0.595 |  | 0.070 | 0.301 |

**Note.** PSG variables: LPS, latency to persistent sleep (min); TST, total sleep time (min); SE, sleep efficiency (%); NAW, number of awakenings; REM, rapid eye movement; Stage 1, duration of stage 1 sleep (min); Stage 2, duration of stage 2 sleep (min); Stage 4, duration of stage 4 sleep (min); SWS, slow wave sleep; SWA, slow wave activity (µV^2^); SWA%, slow wave activity in percentage of total power; SFA, sigma activity (µV^2^); SFA%, sigma activity in percentage of total power. Number of observations for all four factors for men is as follows: n = 79 for SWA, SWA%, SFA and SFA%, n = 87 for all remaining variables. Number of observations for all four factors for women is as follows: n = 100 for SWA, SWA%, SFA and SFA%, n = 113 for all remaining variables.
