# Supplementary material for: Rapid Eye Movement Sleep, Sleep Continuity and Slow Wave Sleep as Predictors of Cognition, Mood, and Subjective Sleep Quality in Healthy Men and Women, Aged 20–84 Years
Source: Front Psychiatry. 2018 Jun 22;9:255. doi: 10.3389/fpsyt.2018.00255 (PMC6024010; doi:10.3389/fpsyt.2018.00255)
Supplement: Supplemental Table 13 — Correlation between PSG variables and cognition factors by age groups controlling for sex and age. [file Table_13.DOCX]

**Supplemental Table 13.** Correlation between PSG variables and cognition factors by age groups controlling for sex and age.

|  |  | **Cognition factor, Kendall's Tau and p-values** | | | | | | | | | | |
| --- | --- | --- | --- | --- | --- | --- | --- | --- | --- | --- | --- | --- |
|  |  | negMood/Arousal | |  | Response time | |  | Accuracy | |  | Visual-Perceptual Sensitivity | |
| **PSG Sleep variable** | **Age group** | *τ* | *p-value* |  | *τ* | *p-value* |  | *τ* | *p-value* |  | *τ* | *p-value* |
| LPS | *Y* | 0.048 | 0.575 |  | 0.050 | 0.560 |  | 0.030 | 0.725 |  | 0.006 | 0.941 |
|  | *M* | 0.103 | 0.196 |  | -0.045 | 0.574 |  | -0.144 | 0.072 |  | -0.003 | 0.967 |
|  | *O* | 0.057 | 0.511 |  | 0.077 | 0.370 |  | -0.057 | 0.511 |  | 0.036 | 0.674 |
| TST | *Y* | -0.030 | 0.728 |  | -0.027 | 0.748 |  | 0.098 | 0.252 |  | -0.080 | 0.350 |
|  | *M* | -0.080 | 0.317 |  | -0.085 | 0.288 |  | -0.027 | 0.732 |  | -0.071 | 0.373 |
|  | *O* | 0.013 | 0.879 |  | 0.048 | 0.575 |  | 0.186 | 0.031 |  | -0.102 | 0.236 |
| SE | *Y* | 0.005 | 0.951 |  | -0.086 | 0.312 |  | 0.124 | 0.147 |  | -0.108 | 0.207 |
|  | *M* | -0.114 | 0.155 |  | -0.117 | 0.142 |  | -0.058 | 0.470 |  | -0.083 | 0.299 |
|  | *O* | 0.001 | 0.987 |  | 0.056 | 0.518 |  | 0.162 | 0.060 |  | -0.099 | 0.251 |
| NAW | *Y* | 0.044 | 0.604 |  | 0.038 | 0.654 |  | -0.099 | 0.250 |  | 0.071 | 0.410 |
|  | *M* | -0.022 | 0.787 |  | 0.066 | 0.409 |  | -0.135 | 0.092 |  | 0.100 | 0.212 |
|  | *O* | 0.036 | 0.677 |  | 0.049 | 0.572 |  | -0.218 | 0.012 |  | -0.089 | 0.305 |
| REM | *Y* | -0.008 | 0.921 |  | 0.031 | 0.717 |  | 0.102 | 0.234 |  | -0.067 | 0.434 |
|  | *M* | 0.071 | 0.372 |  | -0.053 | 0.508 |  | 0.031 | 0.695 |  | -0.104 | 0.193 |
|  | *O* | -0.062 | 0.472 |  | 0.033 | 0.706 |  | 0.034 | 0.694 |  | -0.102 | 0.236 |
| Stage 1 | *Y* | 0.016 | 0.848 |  | 0.039 | 0.649 |  | -0.059 | 0.493 |  | -0.038 | 0.660 |
|  | *M* | -0.135 | 0.090 |  | 0.045 | 0.577 |  | 0.139 | 0.082 |  | 0.064 | 0.424 |
|  | *O* | -0.033 | 0.701 |  | -0.078 | 0.367 |  | -0.131 | 0.128 |  | -0.097 | 0.264 |
| Stage 2 | *Y* | -0.056 | 0.514 |  | -0.162 | 0.058 |  | -0.024 | 0.779 |  | -0.026 | 0.759 |
|  | *M* | -0.076 | 0.344 |  | 0.101 | 0.206 |  | -0.020 | 0.798 |  | 0.074 | 0.352 |
|  | *O* | -0.041 | 0.638 |  | -0.094 | 0.278 |  | 0.137 | 0.113 |  | -0.145 | 0.092 |
| Stage 4 | *Y* | -0.053 | 0.532 |  | 0.079 | 0.355 |  | 0.044 | 0.609 |  | -0.039 | 0.652 |
|  | *M* | 0.010 | 0.900 |  | -0.194 | 0.015 |  | 0.026 | 0.742 |  | -0.085 | 0.285 |
|  | *O* | 0.069 | 0.421 |  | 0.106 | 0.218 |  | 0.041 | 0.634 |  | 0.162 | 0.060 |
| SWS | *Y* | -0.023 | 0.789 |  | 0.122 | 0.155 |  | 0.030 | 0.722 |  | 0.023 | 0.787 |
|  | *M* | -0.004 | 0.957 |  | -0.208 | 0.009 |  | -0.069 | 0.387 |  | -0.109 | 0.171 |
|  | *O* | 0.075 | 0.386 |  | 0.156 | 0.071 |  | 0.039 | 0.649 |  | 0.158 | 0.066 |
| SWA | *Y* | 0.122 | 0.165 |  | 0.006 | 0.950 |  | 0.016 | 0.852 |  | -0.001 | 0.990 |
|  | *M* | 0.050 | 0.548 |  | -0.207 | 0.013 |  | 0.065 | 0.434 |  | -0.119 | 0.156 |
|  | *O* | -0.018 | 0.852 |  | -0.025 | 0.799 |  | 0.063 | 0.514 |  | -0.015 | 0.880 |
| SWA% | *Y* | -0.031 | 0.724 |  | 0.003 | 0.971 |  | 0.119 | 0.175 |  | 0.015 | 0.867 |
|  | *M* | 0.182 | 0.030 |  | -0.087 | 0.298 |  | 0.052 | 0.533 |  | -0.119 | 0.154 |
|  | *O* | 0.023 | 0.812 |  | -0.057 | 0.552 |  | 0.045 | 0.644 |  | -0.067 | 0.488 |
| SFA | *Y* | 0.058 | 0.512 |  | -0.126 | 0.150 |  | 0.075 | 0.394 |  | 0.068 | 0.437 |
|  | *M* | -0.145 | 0.082 |  | -0.009 | 0.912 |  | 0.113 | 0.178 |  | -0.046 | 0.583 |
|  | *O* | -0.026 | 0.786 |  | -0.007 | 0.938 |  | -0.002 | 0.983 |  | 0.008 | 0.931 |
| SFA% | *Y* | -0.032 | 0.716 |  | -0.151 | 0.086 |  | 0.010 | 0.908 |  | 0.095 | 0.280 |
|  | *M* | -0.204 | 0.015 |  | 0.107 | 0.201 |  | 0.083 | 0.319 |  | 0.057 | 0.498 |
|  | *O* | 0.022 | 0.816 |  | -0.009 | 0.927 |  | 0.049 | 0.614 |  | 0.031 | 0.752 |

**Note.** PSG variables: LPS, latency to persistent sleep (min); TST, total sleep time (min); SE, sleep efficiency (%); NAW, number of awakenings; REM, rapid eye movement; Stage 1, duration of stage 1 sleep (min); Stage 2, duration of stage 2 sleep (min); Stage 4, duration of stage 4 sleep (min); SWS, slow wave sleep; SWA, slow wave activity (µV^2^); SWA%, slow wave activity in percentage of total power; SFA, sigma activity (µV^2^); SFA%, sigma activity in percentage of total power. Number of observations for all four factors is as follows (young group): n = 61 for SWA, SWA%, SFA and SFA%, n = 64 for all remaining variables. Number of observations for all four factors is as follows (middle-aged group): n = 67 for SWA, SWA%, SFA and SFA%, n = 73 for all remaining variables. Number of observations for all four factors is as follows (older group): n = 51 for SWA, SWA%, SFA and SFA%, n = 63 for all remaining variables.
