# Supplementary material for: Rapid Eye Movement Sleep, Sleep Continuity and Slow Wave Sleep as Predictors of Cognition, Mood, and Subjective Sleep Quality in Healthy Men and Women, Aged 20–84 Years
Source: Front Psychiatry. 2018 Jun 22;9:255. doi: 10.3389/fpsyt.2018.00255 (PMC6024010; doi:10.3389/fpsyt.2018.00255)
Supplement: Supplemental Table 14 — Correlation between self-reported sleep variables and cognition factors by sex controlling for age. [file Table_14.DOCX]

**Supplemental Table 14.** Correlation between self-reported sleep variables and cognition factors by sex controlling for age.

|  |  | **Cognition factor, Kendall's Tau and p-values** | | | | | | | | | | |
| --- | --- | --- | --- | --- | --- | --- | --- | --- | --- | --- | --- | --- |
|  |  | negMood/Arousal | |  | Response time | |  | Accuracy | |  | Visual-Perceptual Sensitivity | |
| **Subjective Sleep variable** | **Sex** | *τ* | *p-value* |  | *τ* | *p-value* |  | *τ* | *p-value* |  | *τ* | *p-value* |
| SRuA | *M* | **-0.240** | **0.001** |  | 0.075 | 0.288 |  | 0.147 | 0.038 |  | 0.180 | 0.011 |
|  | *F* | -0.112 | 0.077 |  | 0.153 | 0.016 |  | 0.208 | 0.001 |  | -0.002 | 0.980 |
| sSleep-Lat | *M* | 0.210 | 0.003 |  | 0.038 | 0.592 |  | -0.051 | 0.472 |  | -0.146 | 0.040 |
|  | *F* | 0.092 | 0.147 |  | -0.046 | 0.469 |  | -0.052 | 0.409 |  | -0.011 | 0.867 |
| sNAW | *M* | 0.034 | 0.633 |  | 0.013 | 0.856 |  | -0.052 | 0.464 |  | -0.032 | 0.648 |
|  | *F* | 0.087 | 0.168 |  | -0.019 | 0.759 |  | -0.018 | 0.779 |  | 0.121 | 0.057 |
| sQoS | *M* | **-0.239** | **0.001** |  | 0.004 | 0.950 |  | 0.134 | 0.059 |  | 0.113 | 0.111 |
|  | *F* | -0.076 | 0.230 |  | 0.128 | 0.043 |  | 0.157 | 0.013 |  | -0.034 | 0.592 |

**Note.** Bold values indicate significance levels of 0.05 that remain following FDR (False-Discovery Rate procedure as proposed by Benjamini–Hochberg–Yekutieli) correction. Self-reported sleep variables are described in full in Table 3. Number of observations for all four factors for men is n = 92 for all the variables. Number of observations for all four factors for women is n = 114 for all the variables.
