# Supplementary material for: Rapid Eye Movement Sleep, Sleep Continuity and Slow Wave Sleep as Predictors of Cognition, Mood, and Subjective Sleep Quality in Healthy Men and Women, Aged 20–84 Years
Source: Front Psychiatry. 2018 Jun 22;9:255. doi: 10.3389/fpsyt.2018.00255 (PMC6024010; doi:10.3389/fpsyt.2018.00255)
Supplement: Supplemental Table 15 — Correlation between self-reported sleep variables and cognition factors by age groups controlling for sex and age. [file Table_15.DOCX]

**Supplemental Table 15.** Correlation between self-reported sleep variables and cognition factors by age groups controlling for sex and age.

|  |  |  |  |  |  |  |  |  |  |  |  |  |
| --- | --- | --- | --- | --- | --- | --- | --- | --- | --- | --- | --- | --- |
|  |  | **Cognition factor, Kendall's Tau and p-values** | | | | | | | | | | |
|  |  | negMood/Arousal | |  | Response time | |  | Accuracy | |  | Visual-Perceptual Sensitivity | |
| **Subjective Sleep variable** | **Age group** | *τ* | *p-value* |  | *τ* | *p-value* |  | *τ* | *p-value* |  | *τ* | *p-value* |
| sRuA ^Φ^ | *Y* | -0.157 | 0.062 |  | -0.040 | 0.638 |  | 0.051 | 0.546 |  | 0.010 | 0.904 |
|  | *M* | -0.060 | 0.445 |  | **0.276** | **0.000** |  | 0.169 | 0.031 |  | 0.118 | 0.133 |
|  | *O* | -0.242 | 0.005 |  | 0.076 | 0.376 |  | 0.199 | 0.020 |  | 0.141 | 0.101 |
| sSleep-Lat | *Y* | 0.123 | 0.144 |  | -0.031 | 0.711 |  | -0.105 | 0.214 |  | 0.041 | 0.628 |
|  | *M* | 0.154 | 0.049 |  | 0.088 | 0.262 |  | -0.009 | 0.911 |  | -0.068 | 0.387 |
|  | *O* | 0.117 | 0.173 |  | 0.078 | 0.360 |  | 0.010 | 0.903 |  | -0.217 | 0.011 |
| sNAW | *Y* | -0.076 | 0.368 |  | 0.039 | 0.643 |  | -0.111 | 0.186 |  | 0.140 | 0.095 |
|  | *M* | 0.126 | 0.107 |  | -0.089 | 0.254 |  | -0.059 | 0.451 |  | 0.025 | 0.745 |
|  | *O* | 0.146 | 0.089 |  | 0.131 | 0.127 |  | 0.045 | 0.600 |  | -0.015 | 0.862 |
| sQoS | *Y* | -0.072 | 0.392 |  | 0.041 | 0.624 |  | 0.130 | 0.124 |  | -0.042 | 0.615 |
|  | *M* | -0.127 | 0.104 |  | 0.118 | 0.130 |  | 0.107 | 0.169 |  | 0.028 | 0.724 |
|  | *O* | -0.209 | 0.015 |  | 0.086 | 0.314 |  | 0.156 | 0.069 |  | 0.122 | 0.153 |

**Note.** Bold values indicate significance levels of 0.05 that remain following FDR (False-Discovery Rate procedure as proposed by Benjamini–Hochberg–Yekutieli) correction. Φ indicates significant (p < 0.05) differences between middle-aged and young Kendall’s Tau-values. Self-reported sleep variables are described in full in Table 3. Number of observations for all four factors is (young group) n = 66 for all the variables. Number of observations for all four factors is (middle-age group) n = 76 for all the variables. Number of observations for all four factors is (older group) n = 64 for all the variables.
