# Supplementary figures and images for: Rapid Eye Movement Sleep, Sleep Continuity and Slow Wave Sleep as Predictors of Cognition, Mood, and Subjective Sleep Quality in Healthy Men and Women, Aged 20–84 Years
Source: Front Psychiatry. 2018 Jun 22;9:255. doi: 10.3389/fpsyt.2018.00255 (PMC6024010; doi:10.3389/fpsyt.2018.00255)

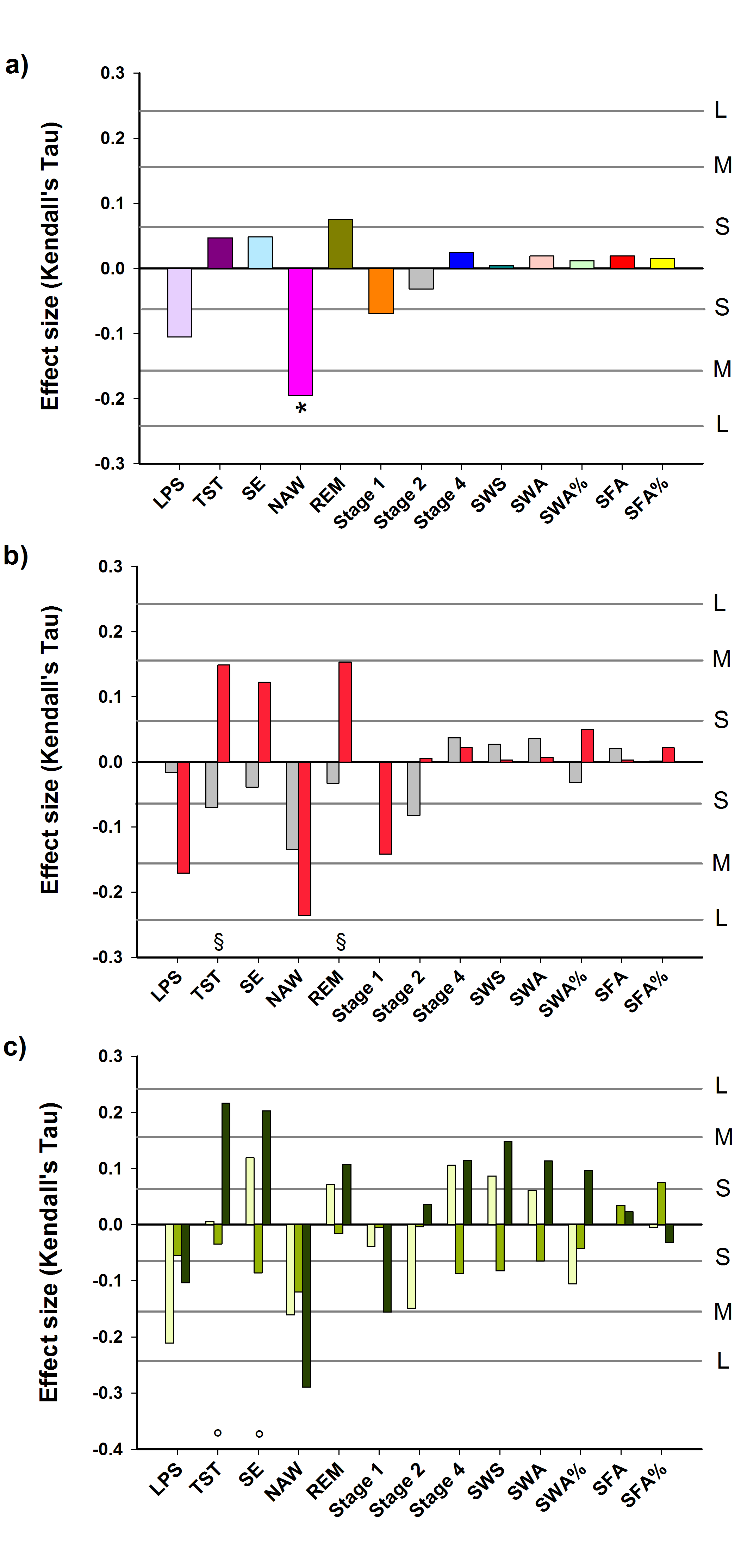

Supplement: Supplemental Figure 1 — Association between EEG sleep parameters and subjective assessment of refreshed upon awakening: (A) data controlled for sex and age; (B) data controlled for age and shown separately for men (gray bars) and women (red bars); (C) data controlled for age and sex and shown separately for three age groups: Young, 20–30 years (yellow bars); Middle-aged, 31–64 years (light green bars); and Older, 65–84 years (dark green bars). For reference, horizontal lines indicate the corresponding Cohen's d effect size: S, small, d = 0.2; M, medium, d = 0.5; H, high, d = 0.8. * indicate significant effects following FDR (False-Discovery Rate) correction (p < 0.05). § indicates a significant (p < 0.05) difference between men and women Kendall's Tau-values. ○ indicates a significant (p < 0.05) difference between middle-aged and older Kendall's Tau-values. LPS, latency to persistent sleep (min); TST, total sleep time (min); SE, sleep efficiency (%); NAW, number of awakenings; REM, rapid eye movement; Stage 1, duration of stage 1 sleep (min); Stage 2, duration of stage 2 sleep (min); Stage 4, duration of stage 4 sleep (min); SWS, slow wave sleep; SWA, slow wave activity (μV2); SWA%, slow wave activity in percentage of total power; SFA, sigma activity (μV2); SFA%, sigma activity in percentage of total power. [file Image_1.TIF]

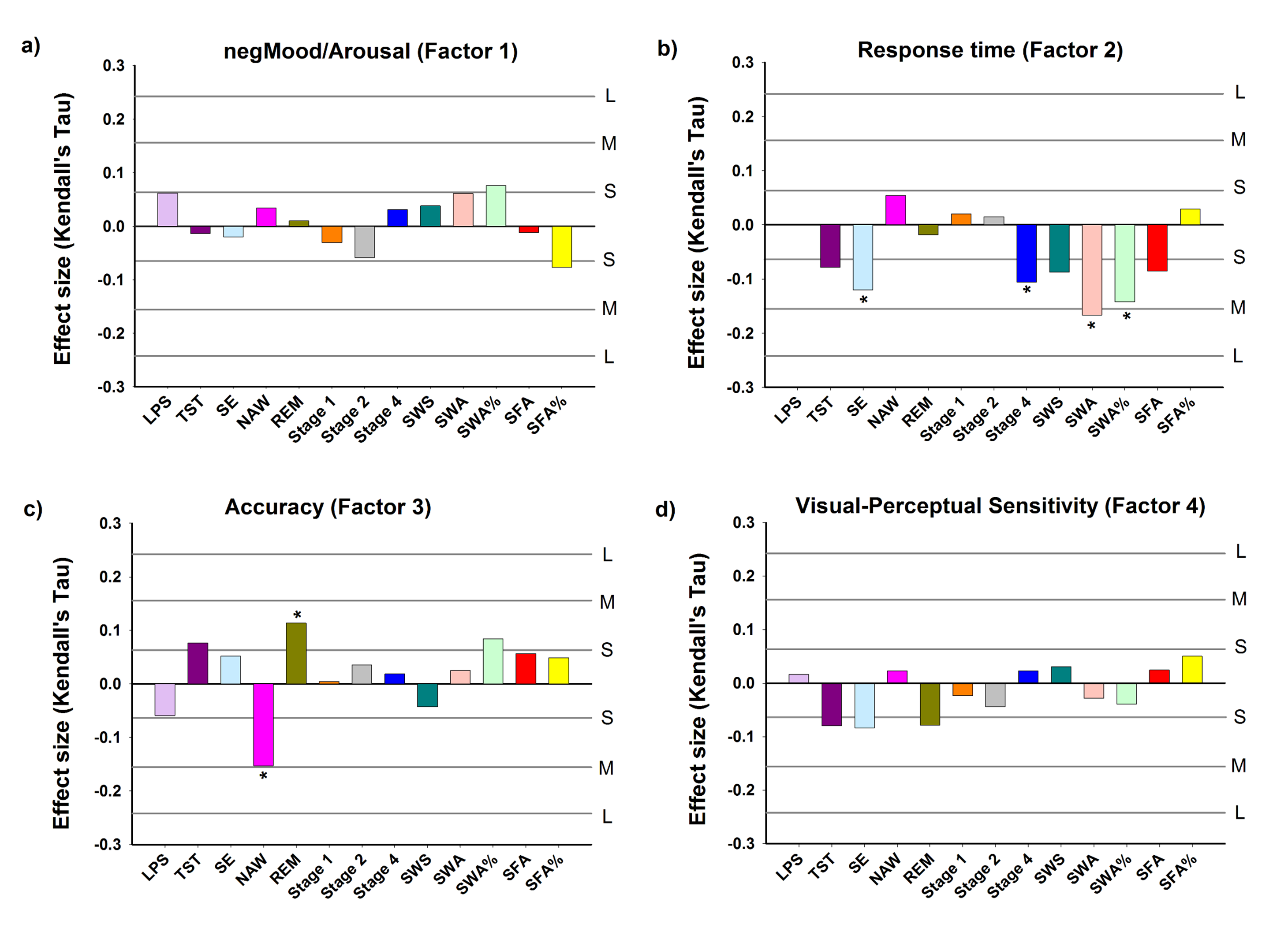

Supplement: Supplemental Figure 2 — Association between EEG sleep parameters and performance factors controlled for age and sex: (A) negMood/Arousal (Factor 1); (B) Response time (Factor 2); (C) Accuracy (Factor 3); (D) Visual-Perceptual Sensitivity (Factor 4). For reference, horizontal lines indicate the corresponding Cohen's d effect size: S, small, d = 0.2; M, medium, d = 0.5; H, high, d = 0.8. * indicate significant effects (p < 0.05). LPS, latency to persistent sleep (min); TST, total sleep time (min); SE, sleep efficiency (%); NAW, number of awakenings; REM, rapid eye movement; Stage 1, duration of stage 1 sleep (min); Stage 2, duration of stage 2 sleep (min); Stage 4, duration of stage 4 sleep (min); SWS, slow wave sleep; SWA, slow wave activity (μV2); SWA%, slow wave activity in percentage of total power; SFA, sigma activity (μV2); SFA%, sigma activity in percentage of total power. [file Image_2.TIF]

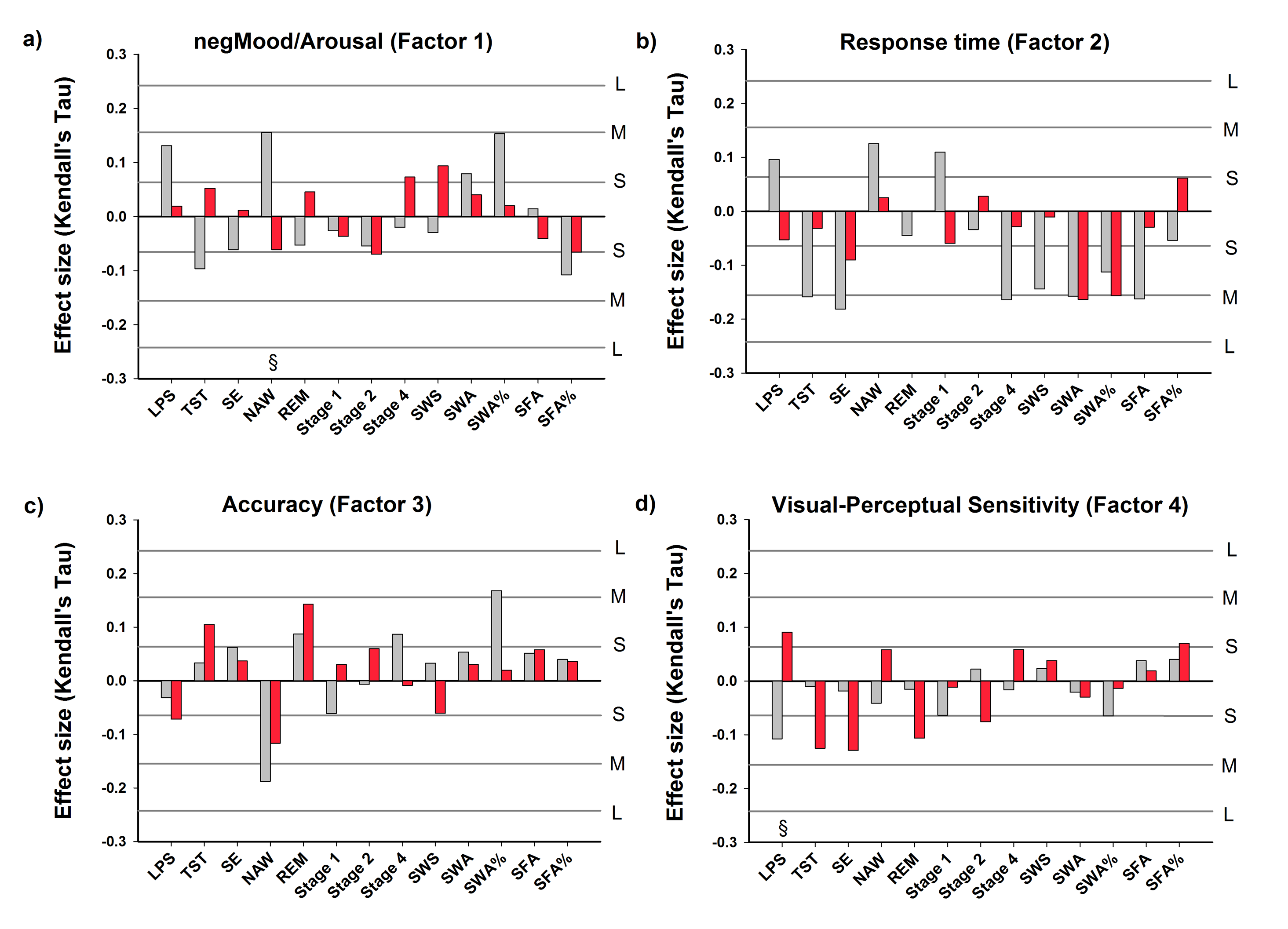

Supplement: Supplemental Figure 3 — Association between EEG sleep parameters and performance factors shown separately for men (gray bars) and women (red bars): (A) negMood/Arousal (Factor 1); (B) Response time (Factor 2); (C) Accuracy (Factor 3); (D) Visual-Perceptual Sensitivity (Factor 4). For reference, horizontal lines indicate the corresponding Cohen's d effect size: S, small, d = 0.2; M, medium, d = 0.5; H, high, d = 0.8. * indicate significant effects (p < 0.05). § indicates a significant (p < 0.05) difference between men and women Kendall's Tau-values. LPS, latency to persistent sleep (min); TST, total sleep time (min); SE, sleep efficiency (%); NAW, number of awakenings; REM, rapid eye movement; Stage 1, duration of stage 1 sleep (min); Stage 2, duration of stage 2 sleep (min); Stage 4, duration of stage 4 sleep (min); SWS, slow wave sleep; SWA, slow wave activity (μV2); SWA%, slow wave activity in percentage of total power; SFA, sigma activity (μV2); SFA%, sigma activity in percentage of total power. [file Image_3.TIF]

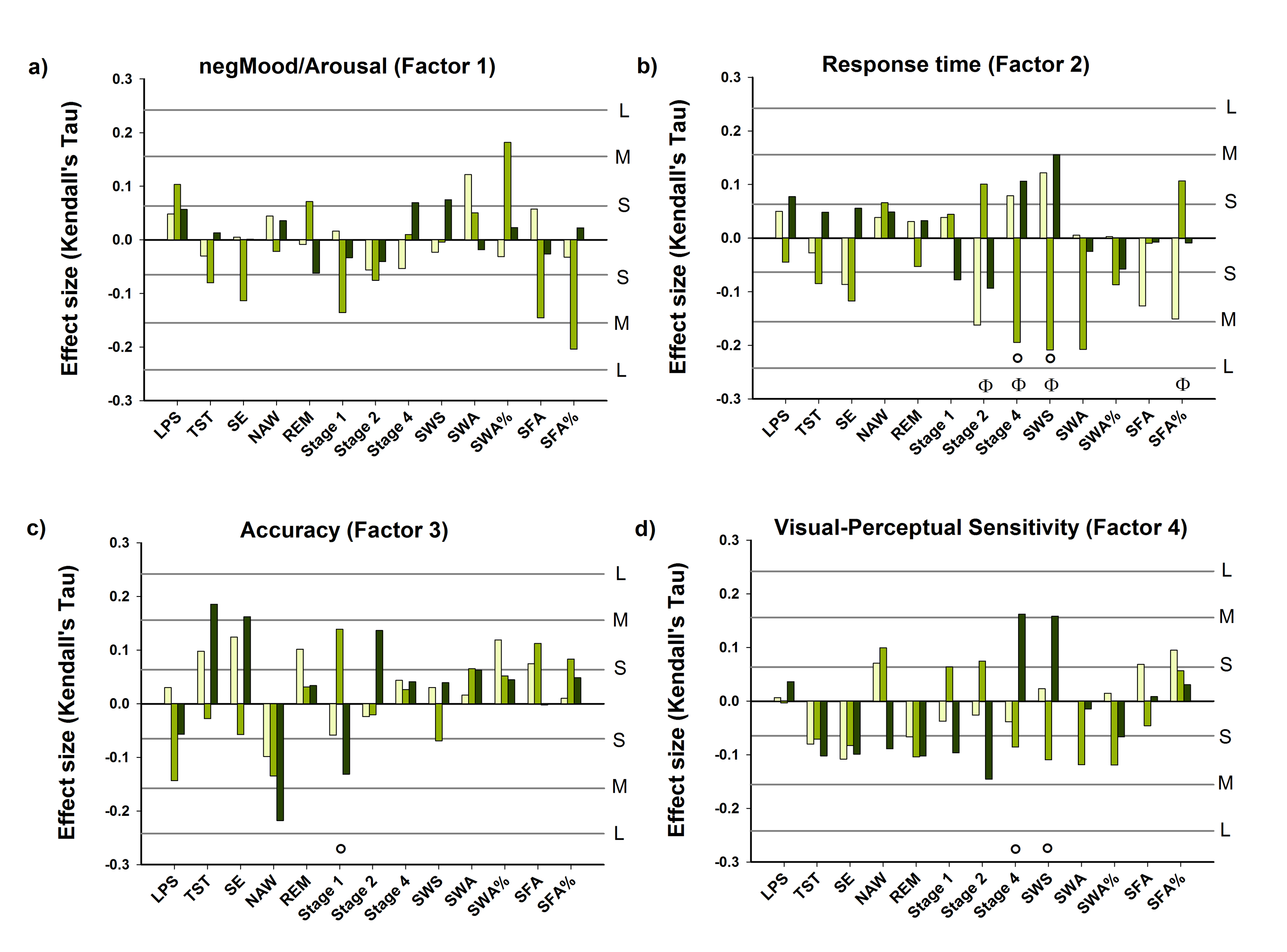

Supplement: Supplemental Figure 4 — Association between EEG sleep parameters and performance factors controlled for age shown separately for three age groups: Young, 20–30 years (yellow bars); Middle-aged, 31–64 years (light green bars); and Older, 65–84 years (dark green bars): (A) negMood/Arousal (Factor 1); (B) Response time (Factor 2); (C) Accuracy (Factor 3); (D) Visual-Perceptual Sensitivity (Factor 4). For reference, horizontal lines indicate the corresponding Cohen's d effect size: S, small, d = 0.2; M, medium, d = 0.5; H, high, d = 0.8. * indicate significant effects (p < 0.05). § indicates significant (p < 0.05) differences between men vs. women Kendall's Tau-values. ∙ indicates significant (p < 0.05) differences between young vs. older Kendall's Tau-values. Indicates significant (p < 0.05) differences between middle-aged vs. older Kendall's Tau-values. Φ indicates significant (p < 0.05) differences between middle-aged and young Kendall's Tau-values. LPS, latency to persistent sleep (min); TST, total sleep time (min); SE, sleep efficiency (%); NAW, number of awakenings; REM, rapid eye movement; Stage 1, duration of stage 1 sleep (min); Stage 2, duration of stage 2 sleep (min); Stage 4, duration of stage 4 sleep (min); SWS, slow wave sleep; SWA, slow wave activity (μV2); SWA%, slow wave activity in percentage of total power; SFA, sigma activity (μV2); SFA%, sigma activity in percentage of total power. [file Image_4.TIF]

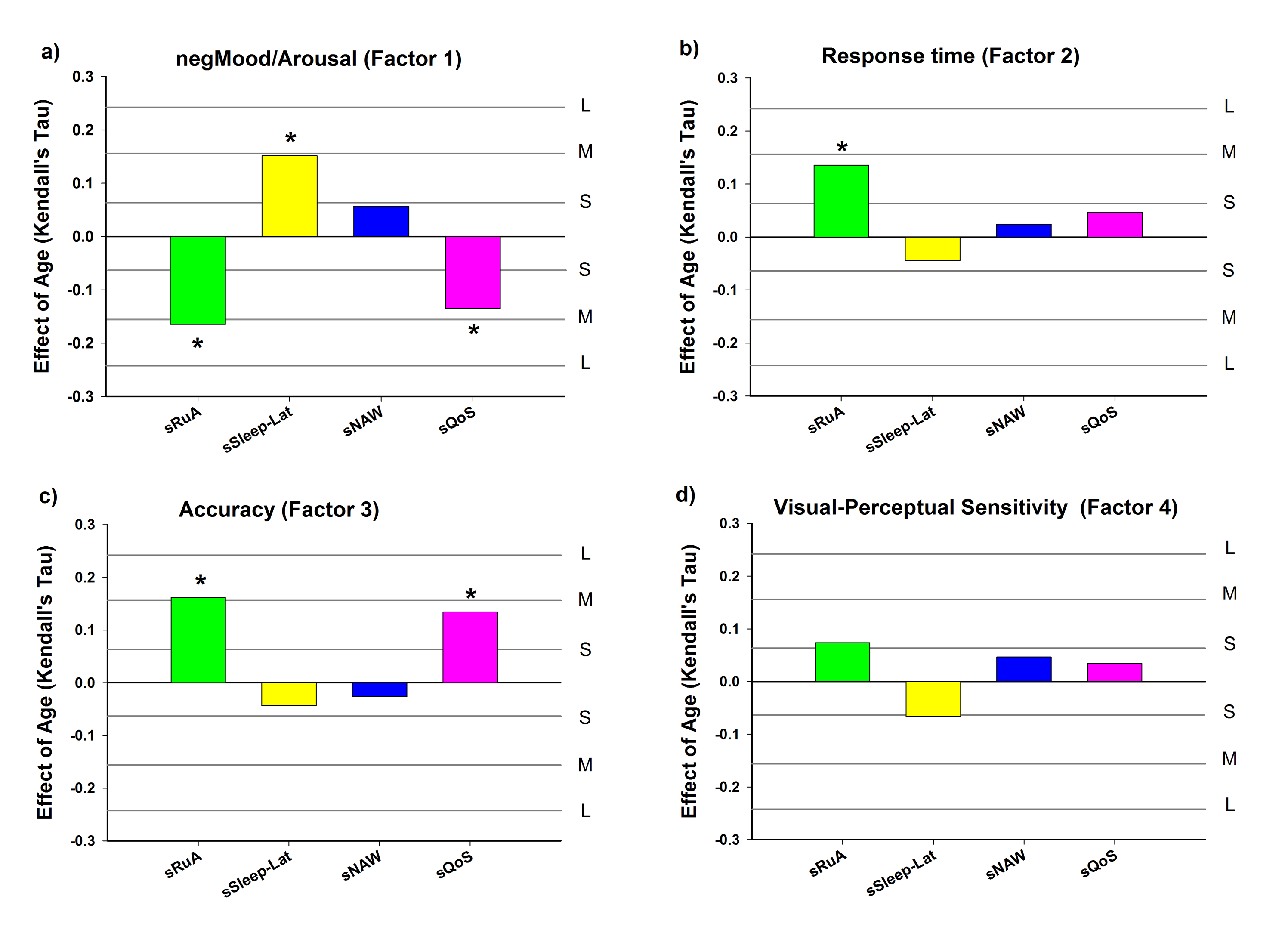

Supplement: Supplemental Figure 5 — Association between self-reported sleep parameters and performance factors controlled for sex: (A) negMood/Arousal (Factor 1); (B) Response time (Factor 2); (C) Accuracy (Factor 3); (D) Visual-Perceptual Sensitivity (Factor 4). For reference, horizontal lines indicate the corresponding Cohen's d effect size: S, small, d = 0.2; M, medium, d = 0.5; H, high, d = 0.8. * indicate significant effects following FDR (False-Discovery Rate) correction (p < 0.05). sRuA, subjective assessment of refreshed upon awakening; sSleep-Lat, subjective sleep latency; sNAW, subjective number of awakenings; sQoS, subjective quality of sleep. [file Image_5.TIF]

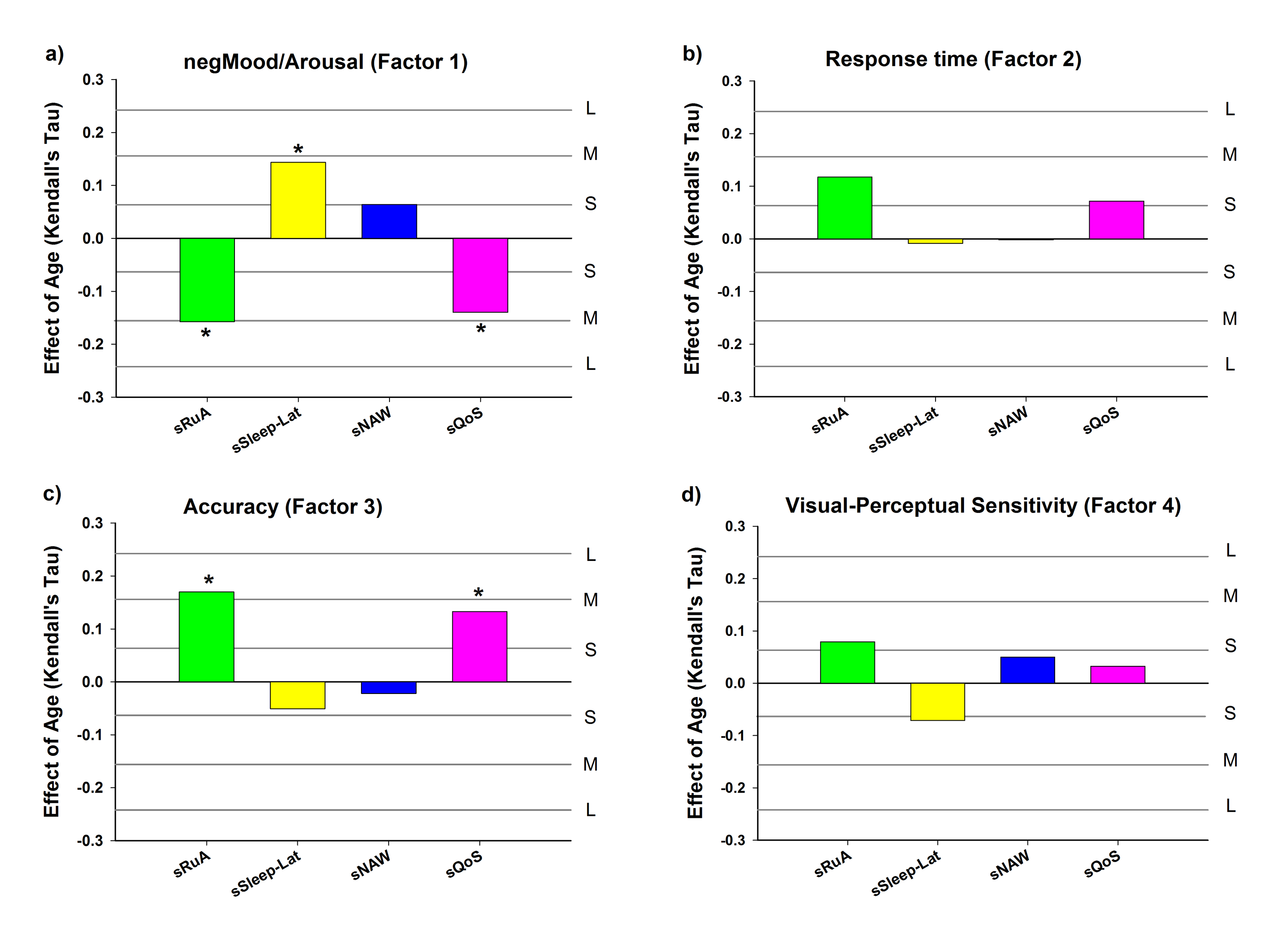

Supplement: Supplemental Figure 6 — Association between self-reported sleep parameters and performance factors controlled for age and sex: (A) negMood/Arousal (Factor 1); (B) Response time (Factor 2); (C) Accuracy (Factor 3); (D) Visual-Perceptual Sensitivity (Factor 4). For reference, horizontal lines indicate the corresponding Cohen's d effect size: S, small, d = 0.2; M, medium, d = 0.5; H, high, d = 0.8. * indicate significant effects following FDR (False-Discovery Rate) correction (p < 0.05). sRuA, subjective assessment of refreshed upon awakening; sSleep-Lat, subjective sleep latency; sNAW, subjective number of awakenings; sQoS, subjective quality of sleep. [file Image_6.TIF]

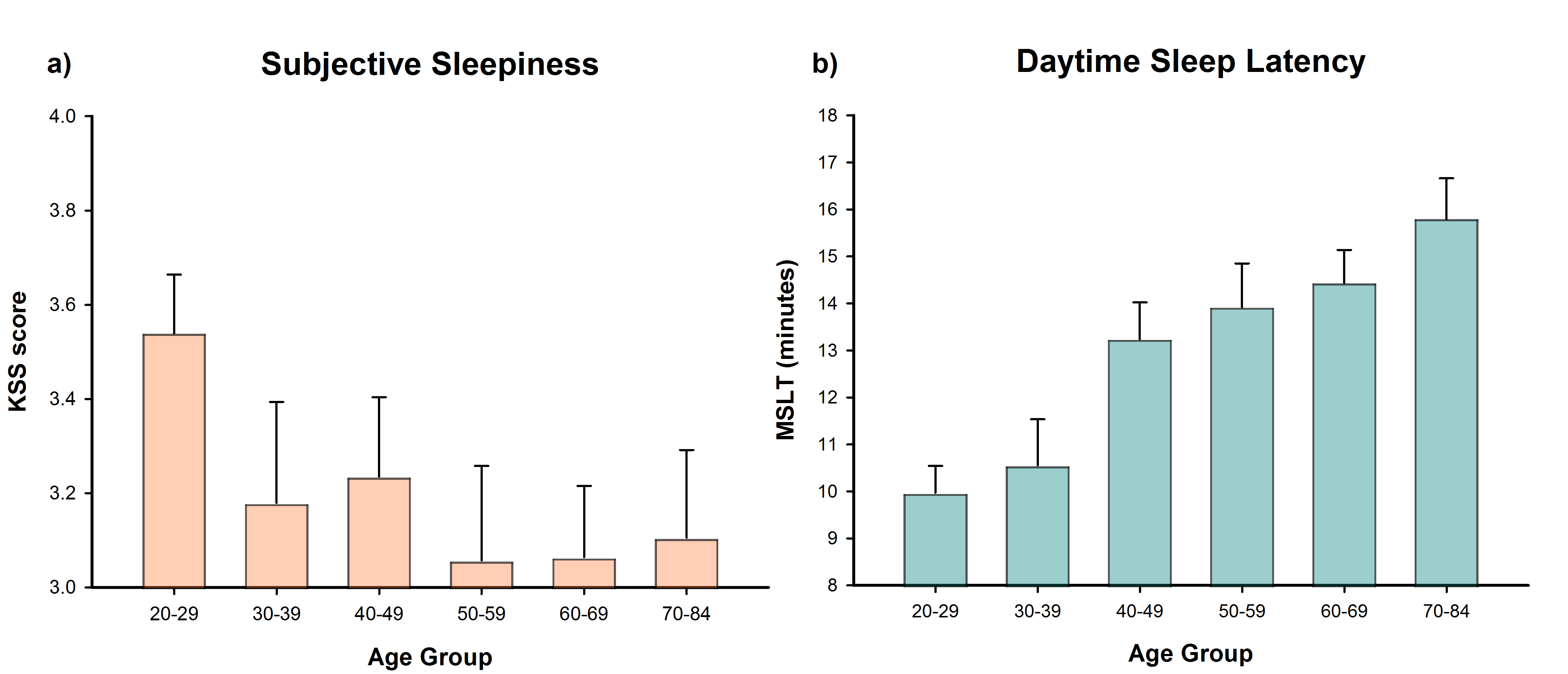

Supplement: Supplemental Figure 7 — KSS & MSLT. Age-related changes combined for males and females (LSmean ± Std Error) in: (A) daytime subjective sleepiness [Karolinska Sleepiness Score (KSS)]; (B) daytime sleep latency [Multiple Sleep Latency Test (MSLT)]. For the KSS there were no statistically significant effects. For MSLT, there was a significant effect of age [F = 9.54, (5, 194), p < 0.0001]; post-hoc tests revealed: 20–29 < 40–49 (p = 0.001), 50–59 (p = 0.0004), 60–69 (p < 0.0001), 70–84 (p < 0.0001), 30–39 < 40–49 (p = 0.0346), 50–59 (p = 0.0142), 60–69 (p = 0.0016), 70–84 (p < 0.0001), 40–49 < 70–84 (p = 0.0309). [file Image_7.TIF]
